# A Digital Framework to Build, Visualize and Analyze a Gene Expression Atlas with Cellular Resolution in Zebrafish Early Embryogenesis

**DOI:** 10.1371/journal.pcbi.1003670

**Published:** 2014-06-19

**Authors:** Carlos Castro-González, Miguel A. Luengo-Oroz, Louise Duloquin, Thierry Savy, Barbara Rizzi, Sophie Desnoulez, René Doursat, Yannick L. Kergosien, María J. Ledesma-Carbayo, Paul Bourgine, Nadine Peyriéras, Andrés Santos

**Affiliations:** 1 Biomedical Image Technologies, ETSIT, Universidad Politécnica de Madrid, CEIMoncloa, Madrid, Spain; 2 Research Center in Bioengineering, Biomaterials and Nanomedicine (CIBER-BBN), Madrid, Spain; 3 Madrid-MIT M+Visión Consortium, Massachusetts Institute of Technology, Cambridge, Massachusetts, United States of America; 4 MDAM UPR3294, Institut de Neurobiologie Alfred Fessard, CNRS, Gif-sur-Yvette, France; 5 Institut des Systèmes Complexes, Paris, France; 6 BioEmergences-IBiSA, Institut de Neurobiologie Alfred Fessard, CNRS, Gif-sur-Yvette, France; 7 School of Biomedical Engineering, Drexel University, Philadelphia, Pennsylvania, United States of America; 8 LIMICS-INSERM UMR 1142, UFR SMBH, Université Paris 13, Bobigny, France; University of Southern California, United States of America

## Abstract

A gene expression atlas is an essential resource to quantify and understand the multiscale processes of embryogenesis in time and space. The automated reconstruction of a prototypic 4D atlas for vertebrate early embryos, using multicolor fluorescence *in situ* hybridization with nuclear counterstain, requires dedicated computational strategies. To this goal, we designed an original methodological framework implemented in a software tool called Match-IT. With only minimal human supervision, our system is able to gather gene expression patterns observed in different *analyzed embryos* with phenotypic variability and map them onto a series of common 3D *templates* over time, creating a 4D atlas. This framework was used to construct an atlas composed of 6 gene expression templates from a cohort of zebrafish early embryos spanning 6 developmental stages from 4 to 6.3 hpf (hours post fertilization). They included 53 specimens, 181,415 detected cell nuclei and the segmentation of 98 gene expression patterns observed in 3D for 9 different genes. In addition, an interactive visualization software, Atlas-IT, was developed to inspect, supervise and analyze the atlas. Match-IT and Atlas-IT, including user manuals, representative datasets and video tutorials, are publicly and freely available online. We also propose computational methods and tools for the quantitative assessment of the gene expression templates at the cellular scale, with the identification, visualization and analysis of coexpression patterns, synexpression groups and their dynamics through developmental stages.

## Author Summary

We propose a workflow to map the expression domains of multiple genes onto a series of 3D templates, or “atlas”, during early embryogenesis. It was applied to the zebrafish at different stages between 4 and 6.3 hpf, generating 6 templates. Our system overcomes the lack of significant morphological landmarks in early development by relying on the expression of a reference gene (goosecoid, *gsc*) and nuclear staining to guide the registration of the analyzed genes. The proposed method also successfully maps gene domains from partially imaged embryos, thus allowing greater microscope magnification and cellular resolution. By using the workflow to construct a spatiotemporal database of zebrafish, we opened the way to a systematic analysis of vertebrate embryogenesis. The atlas database, together with the mapping software (Match-IT), a custom-made visualization platform (Atlas-IT), and step-by-step user guides are available from the Supplementary Material. We expect that this will encourage other laboratories to generate, map, visualize and analyze new gene expression datasets.

This is a *PLOS Computational Biology* Methods article.

## Introduction

Deciphering and integrating the genetic and cellular dynamics underlying morphogenesis and homeostasis in living systems is a major challenge of the post-genomic era. Although full genome sequencing is available for a number of animal model organisms [1], quantitative data for the spatial and temporal expression of genes is still lacking [2].

Remarkable advances in photonic microscopy imaging [3],[4],[5] and labeling techniques [6] allowed gathering data at all levels of a multicellular system's organization with adequate spatial and temporal resolutions. Fluorescent *in situ* hybridization techniques [7], immunocytochemistry and transgenesis, combined with 3D optical sectioning, make it now possible to assess the dynamics of gene expression throughout animal development with precision at the single-cell level. However, moving forward from databases of gene expression that contain average values at low spatiotemporal resolutions—such as those obtained from DNA microarrays available for most model organisms—to a dynamic, cell-based 4D atlas is a major paradigm shift that requires the development of appropriate methods and tools.

In this context, the design and implementation of automated image analysis strategies to build a gene expression atlas with resolution at the cellular scale is an important methodological bottleneck towards greater biological insights [8],[9]. The task of assembling imaging data from cohorts of individuals, or *analyzed embryos*, onto a series of 3D prototypes, or *templates* (one per developmental stage), can be approached by finding a spatial correspondence between individuals based on registration methods, a technique used in medical imaging [10]. Yet, gathering and consolidating into a single prototype multimodal and multiscale features from different specimens that exhibit phenotypic variability remains a difficult challenge.

Recent studies on different model organisms have explored computational strategies for building atlases either by measuring cell positions to create prototypic specimens [11],[12] or by gathering gene expression patterns observed in cohorts of specimens [13],[14],[15],[16]. Yet, very few frameworks have combined both features. Long et al. [11] collected data from 15 *C. elegans* specimens at the earliest larval stage (L1 with 357 cells) to build a statistical 3D atlas of nuclear center positions. *C. elegans* presents a number of advantages facilitating the reconstruction process. The entire organism can be imaged with resolution at the single-cell level and its cell lineage tree is stereotyped enough to allow spatiotemporal matching of different individuals at this level. The same features allowed the reconstruction of a prototypic lineage for a cohort containing six specimens of *Danio rerio* (zebrafish) embryos throughout their first 10 cell division cycles [12]. Peng et al. [15] achieved the spatial matching of 2,945 adult *Drosophila* brains to collect the expression patterns of 470 different genes. Similarly, Lein et al. [13] constructed a comprehensive atlas of the adult mouse brain containing about 20,000 gene patterns. The first gene expression atlas with resolution at the cellular scale was produced by Fowlkes et al. [14]. They integrated 95 gene expression patterns observed at 6 different developmental stages in a total of 1,822 different *Drosophila* embryos within a common 3D stencil.

Applying this approach to vertebrate model organisms is more difficult because of higher cell lineage variability and heterogeneous levels of gene expression within highly dynamic patterns. In addition, the reconstruction of 3D gene expression templates at cellular scale for vertebrate species is likely to require the acquisition of partial volumes recorded at high resolution [15] from single specimens, and their precise mapping onto *in toto* reference specimens. The zebrafish, a vertebrate model organism increasingly used for its relevance to biomedical applications [17], cumulates good properties for investigating the reconstruction of the multiscale dynamics of early embryogenesis. The gene regulatory network (GRN) architecture of the zebrafish early embryonic development is under construction [18] and the embryo is easily accessible and amenable to transgenesis, multiple *in situ* staining and 3D+time imaging. The spatiotemporal data offered by a 4D atlas of gene expression with resolution at the cellular level is expected to provide the necessary measurements for further modeling of the GRN dynamics and possible integration of the genetic and cellular levels of organization [19]. Such data would make the zebrafish the first vertebrate model amenable to a systemic study. However, building 3D templates of gene expression for the zebrafish blastula and gastrula stages is especially problematic due to the lack of morphological landmarks required for the registration of patterns [20],[21].

We provide a methodology to construct, visualize and analyze a gene expression atlas composed of templates at various stages of vertebrate early development. We designed, implemented and now deliver two computational frameworks, Match-IT and Atlas-IT, to support the automatic mapping of 3D gene expression patterns from different individuals (the analyzed embryos) onto common reference specimens (the templates) with resolution at the cellular scale. This “virtual multiplexing” procedure [14] overcomes the limited number of gene products that can be jointly stained and measured in a single specimen.

Match-IT was used to produce the prototypic cartography of 9 gene expression patterns imaged from 3D double fluorescent *in situ* hybridization at 6 developmental stages (**Table S1**, **Movie S1**, **Figs. S1, S2, S3, S4, S5, S6, S7**). Atlas-IT was designed to interactively visualize gene coexpression patterns and their dynamics. We validated our 4D atlas construction methodology by an automated quantitative assessment of gene patterns' similarity and overlap through time. Analytical tools, such as clustering, were designed to identify morphogenetic domains and gene synexpression groups, i.e. groups of genes sharing the same spatiotemporal expression patterns. The proposed spatiotemporal atlas of zebrafish blastula and early gastrula preserves the information of the cell as the gene expressing unit, providing means for the integration of genetic and cellular data unavailable so far.

## Results

### Match-IT: A workflow to build a gene expression atlas

**Figure 1:**
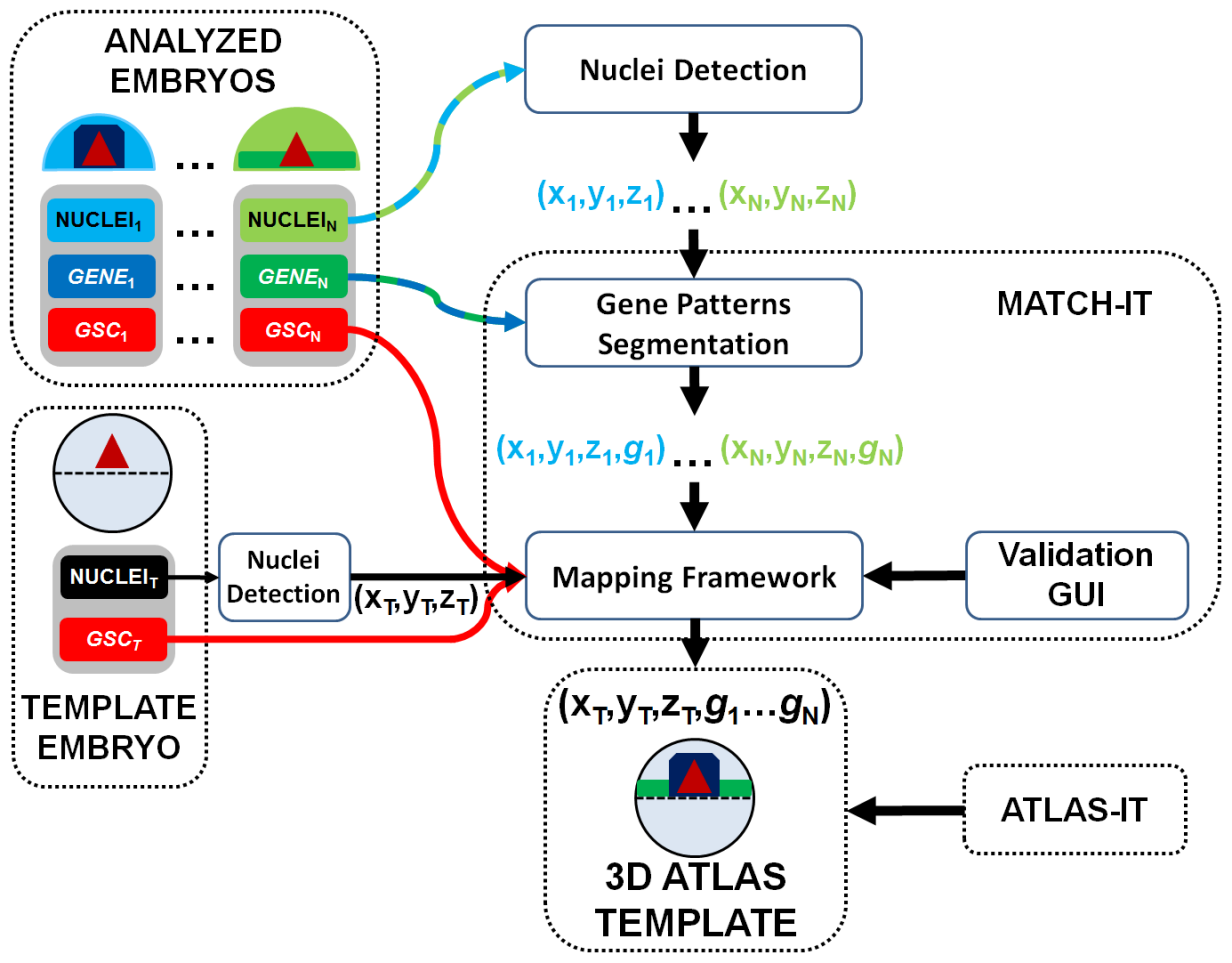
Schematic description of the atlas construction process. For each developmental stage, the partial 3D volumes of the analyzed embryos and the 3D volume of the whole template embryo were processed for nuclear center detection and gene pattern segmentation. Mapping the analyzed embryos onto the corresponding common template was guided by the specimen's shape, revealed by the nuclei, and by the segmented *gsc* expression pattern, chosen to be a common reference. Each step was supervised and, if necessary, corrected via an interactive graphical user interface. The final model, where all the gene patterns coming from different individuals could be jointly compared, constitutes one 3D atlas template. The Match-IT software performs the gene pattern segmentation and the validated mapping. The Atlas-IT software allows interactive visualization of the 3D atlas template.

We designed a computational framework (**Fig. 1**), going from image acquisition to image data analysis, to perform the mapping of different stained gene expression patterns onto a common prototypic model at each developmental stage (**Fig. S8**), thus creating a series of 3D templates of gene expression with resolution at the cellular scale.

**Figure 2:**
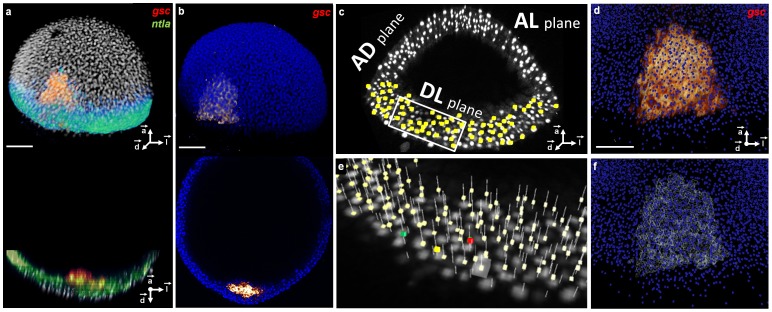
3D raw data, nuclear center detection, gene pattern segmentation and their validation at 6.3 hpf. (**a**) Upper panel: volume rendering, lower panel: axial orthoslice of an analyzed embryo's nuclei (white), reference *gsc* pattern (red) and *ntla* pattern (green). (**b**) Same with template nuclei (blue) and their *gsc* pattern (red). (**c**) Nuclear positions (yellow) superimposed on the raw nucleus images (white) displayed by three orthoslices in the 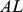, 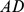 and 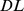 planes. (**d**) Zoom on the template *gsc* raw expression (red) superimposed on the template nuclear positions (blue). (**e**) 3× zoom on the boxed region in (c) with detected nuclei positions (pale yellow), an example of a validated nucleus (green), a false positive (red), a false negative (yellow) and a selected position to be evaluated (white cube). (**f**) Same as (d) with the segmented *gsc* domain (white). Scale bars, 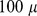
m. Axes point to the animal pole (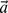), dorsal side (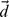) and lateral side (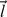) of the embryo respectively.

The processing workflow consisted of embryo staining, image data acquisition (Materials and Methods), nuclear center detection, gene pattern segmentation, mapping of the analyzed embryos onto a template at each stage, and selection of template cells positive for the expression of specific genes. This methodology was designed to document at a sufficient spatial and temporal resolution the gene expression dynamics underlying the formation of the Spemann organizer and the embryonic axis of zebrafish early embryos. To this end, we imaged the dorsal side of fluorescently stained embryos with cellular resolution from fixed specimens about every 30 min from 4 to 6.3 hpf. The resulting 6 templates comprised a stencil of *in toto* 3D images of the template specimens (**Fig. 2a**) at different stages, and mappings of the partial 3D views of the analyzed embryos (**Fig. 2b**).

In order to integrate 3D data into one template, our novel Match-IT tool (**Software S1** and **User Guide S1**) performed the segmentation of gene expression domains, the mapping of analyzed embryos onto a common reference specimen and the identification of positive cells (**Fig. 1** and **Movie S2**), eventually delivering a 3D database that summarized the genetic profile of single cells.

#### Nuclear center detection

Nuclear center detection was an important preliminary step to (1) compute a common referential for all the specimens that will guide the first coarse mapping, (2) keep image registration within the boundaries of a “nuclear mask” around the embryo, and (3) quantitatively analyze gene expression domains from intracellular locations only, without taking into account extracellular space where staining is weaker. Additionally, detecting the nuclei has the advantage that it allows working at the cell level: cell clustering and cell entropy have a biological meaning, whereas working at the voxel level, although theoretically possible, does not have this biological significance.

The detection of cell nuclei was carried out by an algorithm followed by interactive supervision of the parameters through visual inspection (**Fig. 2c,e**). First, nuclear centers were approximately defined at the local maxima of a smoothed, simplified version of the original image. Preprocessing consisted of convolving the image with two Gaussians of different standard deviations ranging from 2 to 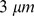 and 8 to 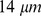 respectively, then calculating their difference and only retaining gray values greater than a threshold, which could vary between 1 and 15%. This procedure smoothed the image while preserving only significant objects. Multiple simulations were automatically run for each combination of parameters in the above ranges of standard deviations and thresholds. Using a visual inspection tool, (**Fig. 2e**) the optimal values were subsequently chosen and validated by an expert through comparison of the raw data with the candidate cell positions from different runs. A quantitative evaluation of this strategy performed on one dataset by comparing the detected centers with 689 manually labeled nuclei produced an error rate of 4% (**Fig. S9a–d**). This error detection rate was considered acceptable to assign positive gene expression at the cellular level and was shown to be robust against possible variations in the parameter choices by the expert (**Fig. S9e**).

#### Gene pattern segmentation

Our segmentation of the gene expression domains first required supervision and selection by a biologist of the lower image intensity values that best defined the domain features. Match-IT then used these parameters to perform a thresholding operation followed by morphological image processing (see Materials and Methods). The result of the expression domain segmentation was validated by visual inspection with Atlas-IT prior to the identification of positive cells within the segmented domain (**Fig. 2d,f**). Alternatively, the amount of fluorescent signal could also be used for relative quantification of gene expression within each specimen at the cellular level (**Fig. S10**). However, a binary expression assignment, such as one provided by segmentation, was also consistent with conventional Boolean GRN modeling [22]. Cells in the analyzed embryos were identified as positive for the expression of a given gene if their approximate nuclear centers were located at less than half the average internuclear distance (**Fig. S11**) from the border of the segmented expression domain.

#### Embryo mapping

**Figure 3:**
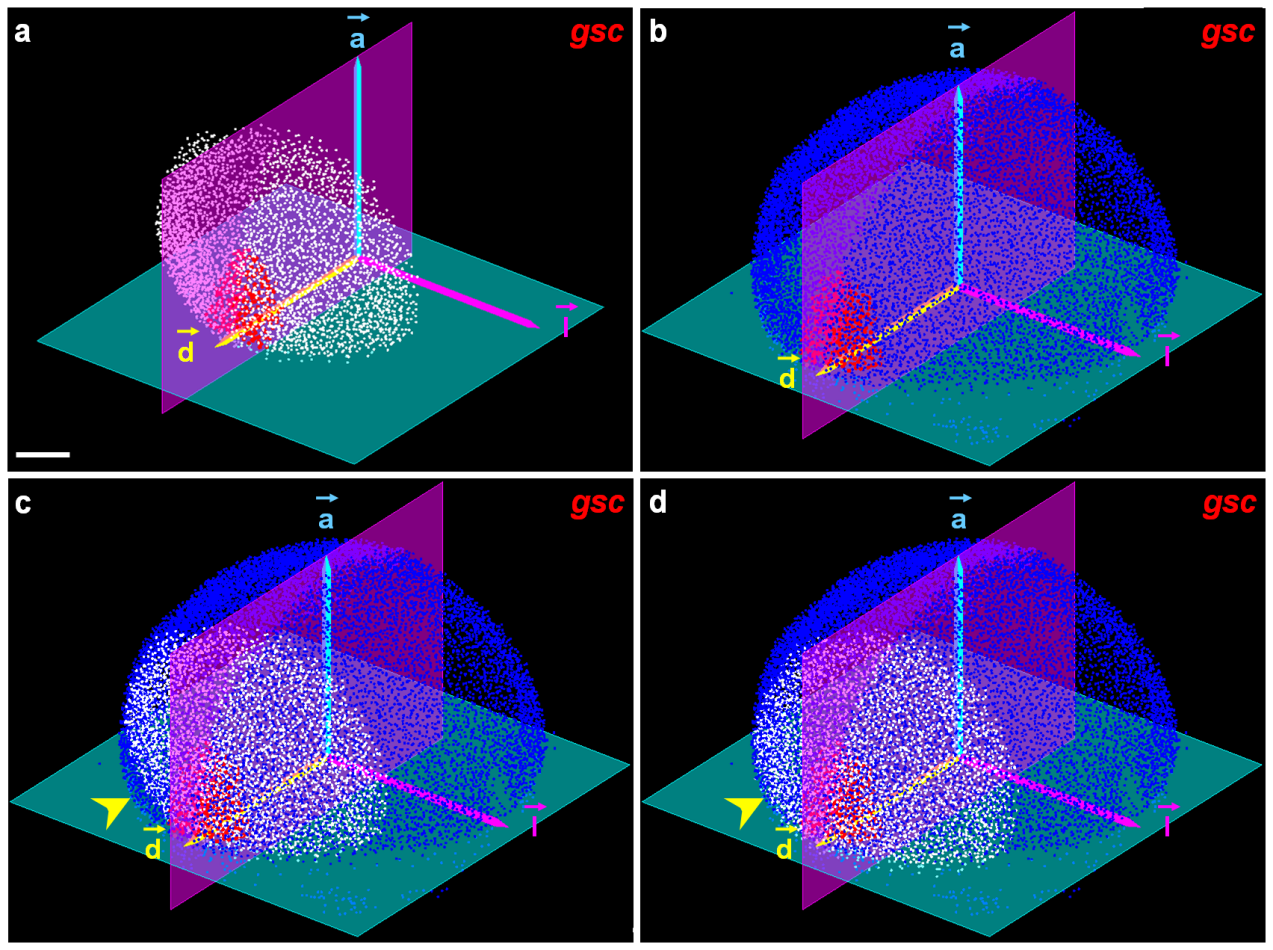
Mapping procedure in the 6.3 hpf atlas template. (**a**) Analyzed embryos: detected nuclei (white), *gsc* positive cells (red), automated initialization scheme extracting the plane passing through the blastoderm margin (green), bilateral symmetry plane (purple), and referential 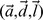. (**b**) Same with the template, detected nuclei in blue. (**c**) Initialization step aligning the 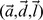 basis of the analyzed embryo and the template; the yellow arrowhead points to a mismatch refined in (**d**) through the registration procedure. Scale bar 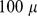
m.

Mapping the partial 3D volumes of the analyzed embryos onto one template involved matching the embryos' common referential, their blastoderm contours and their *gsc*-positive domains (**Fig. 3a–d**). The mapping procedure was a two-step process. First, initialization was based on the automated identification of a common referential (**Fig. 3a,b**) defined by two orthogonal planes 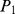 and 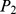. Plane 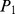 separated the blastoderm from the yolk at the level of the blastoderm margin, while 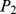 was the bilateral symmetry plane containing both the center of the embryo's spherical approximation and the center of mass of the *gsc*-positive nucleus population, 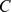 (see Materials and Methods). These two planes unequivocally defined a three-vector basis comprising the animal-vegetal axis (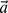), the dorso-ventral axis (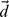) and the perpendicular vector (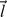) given by the right-handed trihedron. The origin 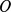 of the reference frame was obtained by projecting 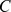 on 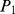 and, with the basis 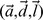, was used to transform the analyzed embryos into the template. The result of this initialization was visually checked and, if necessary, corrected with the Match-IT graphical user interface, designed to minimize the effort of manual supervision (**Fig. 3a–d**).

Second, this coarse initialization step was refined by a pixel-based registration procedure. Considering that zebrafish early embryos largely lacked the distinctive morphological features required to apply landmark-based registration methods [23],[15],[24], and given the partial nature of the volumes to be aligned, we opted for a rigid, pixel-based transformation scheme [25] that searched for an optimal match between dorsal blastoderm surfaces (**Fig. 3c,d**). A preliminary quantification of morphological variability was performed by estimating the embryos' radial size. It showed that 95% of the registered embryos differed by less than 10% from the mean in terms of the radius of the blastoderm plane margin (**Fig. S12**). The rigid transformation preserved original gene patterns, making it possible to go back to the raw data for visualization and validation/correction with the Match-IT software at every step of the processing pipeline. After the final step, the average manual offset needed to adjust the mapping of analyzed embryos onto the template was 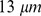 (i.e. approximately one cell row) and a 3° rotation.

#### Positive template cell selection

Finally, the selection of positive template cells (**Fig. S13**) was performed using the same rule described for the identification of positive cells in the analyzed embryos. The number 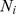 of cells positive for the expression of gene 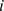 in an analyzed embryo differed from the number 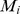 of cells selected as positive in the template after the mapping procedure. Independently from the fluctuations in the mapping procedure mentioned above, this difference was interpreted as resulting from individual variations in terms of internuclear distance and embryo shape, which can reflect staging misalignments (**Fig. S14**).

### Atlas-IT: A visualization tool for a gene expression atlas

Analysis of the 3D templates produced by Match-IT required dedicated visualization tools to test hypotheses and derive biological insights. The available software kits did not fulfill our requirements, either because they were too specific for a given model organism (such as PointCloudXplore [26] for *Drosophila*) or because they were too generic as visualization and processing tools (such as Icy [27], Vaa3D [28], or CellProfiler [29]) and did not allow displaying selections of individual cellular positions or querying a template for coexpression domains with resolution at the cellular scale.

**Figure 4:**
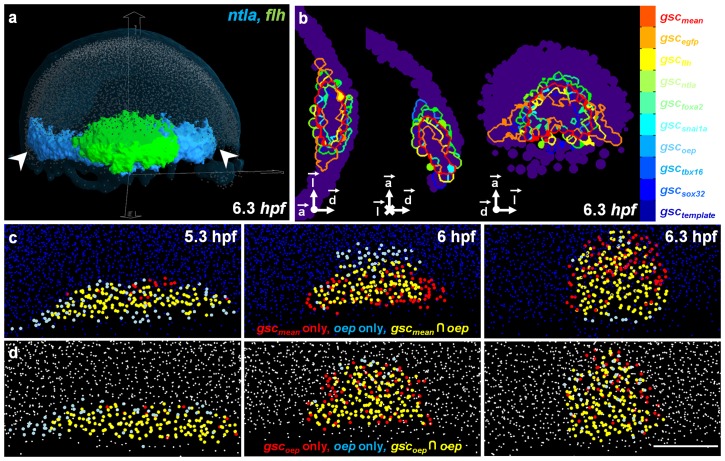
Exploring the 3D atlas with the visualization tool Atlas-IT. (**a**) Atlas-IT interface displaying the template nuclei (light blue), segmented gene expression patterns of *ntla* (blue) and *flh* (green). (**b**) From left to right: equatorial, sagittal and dorsal views of the 9 individual *gsc* boundaries as compared to the mean *gsc* domain (red) at 6.3 hpf. (**c**) Evolution of the *oep*-*gsc* 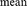 pair over time after being mapped onto the template. (**d**) Evolution of the *oep*-*gsc* 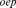 pair over time in the analyzed embryo where they were co-stained. Scale bar 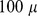
m.

For these reasons, we designed, developed and deliver here the Atlas-IT interactive visualization interface (**Fig. 4a** and **Software S2** and **User Guide S2**) to explore 4D atlas resources. With this tool, we can interact with the complete atlas data, in particular superimpose raw images (either as 3D volumes or orthoslices), segmented patterns, and the whole set of detected template nuclei or selected positive nuclei at any time point (**Movie S3**). Atlas-IT can be used to assess the dynamics of gene coexpression domains or the variability of gene expression patterns.

### A spatiotemporal atlas of the zebrafish early embryo

We used Match-IT and Atlas-IT together to reconstruct a 4D atlas of zebrafish early embryogenesis, which is now released. It comprises 6 developmental stages and 9 gene expression patterns chosen to study a specific embryological question, namely the genetic dynamics underlying the formation of the Spemann organizer at the dorsal midline [1] (a region in the zebrafish containing precursors of the segregation between the prechordal plate and the notochord [30]). The 9 genes are: *gsc*, *sox32*, *tbx16*, *oep*, *snai1a*, *foxa2*, *ntla*, *flh*, and *egfp*, where the latter was was detected in a custom-made transgenic line Tg(−4*gsc*:*egfp*)isc3. These genes appear as nodes in the axial mesendoderm GRN proposed by Chan et al. [18]. In addition, *egfp* allowed us to validate the transgenic line as a faithful reporter of early *gsc* gene expression (**Fig. S15**). The time series of 3D templates was chosen to explore gene expression dynamics from the onset of zygotic activation at 3 hpf until early gastrulation, and encompasses the following developmental stages: sphere (4 hpf), dome (4.3 hpf), 30% epiboly (4.7 hpf), 50% epiboly (5.3 hpf), shield (6 hpf) and late shield (6.3 hpf) according to the staging defined at 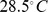. For each new gene expression to be mapped, a cohort of individuals was processed for double *in situ* hybridization and 3 of them were imaged. The atlas construction methodology was established by using one specimen of each cohort (**Table S1**).

### Validation of the atlas to assess the relationships between gene patterns

The atlas was constructed to be able to compare gene expression patterns from different stained specimens. Establishing spatial relationships between gene patterns required assessing gene expression variability and calculating mean expression domains (Materials and Methods). The expectation was that the spatial relationships observed between two genes stained in the same embryo should be maintained between their mean expression domains in a template. The expression of *gsc* was revealed in 9 different specimens, which comprised 8 analyzed embryos and one template, at each developmental stage. It provided a paradigmatic case to calculate a mean expression domain and assess gene variability (**Fig. S16**). At any given stage, we quantified the mean distance from the complete outer surface of each individual *gsc* domain (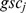) to the closest boundary point of the mean domain (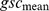), following a leave-one-out protocol (**Fig. 4b**). The measured distance, which reflected both the accuracy of our mapping scheme and the inter-individual variability between the boundaries of the *gsc* expression domains, was on average less than 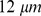, i.e. approximately one cell diameter (**Fig. S17**). This accuracy error remained within the same range independently from the thickness of the three main embryo planes (**Fig. S18**). Additionally, more than 80% of all the individual 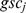 border points were less than one cell row away from the 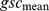 border, indicating that there were no large distance discrepancies along the contours (**Fig. S19**).

To demonstrate that this level of accuracy was maintained in regions far from the *gsc* expression domains, we replicated the same quality measure with another gene, *tbx16*, which spread across a much larger area than *gsc*. With Match-IT, we added two new *tbx16* datasets, *tbx16_b_* and *tbx16_c_*, to the already existing *tbx16_a_* expression in the atlas at 6.3 hpf (**Fig. S20a**). The mean distance from the complete outer surface of each individual *tbx16_j_* domain to the closest boundary point of *tbx16* 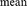 remained under one cell diameter (**Fig. S20b**). Moreover, the histogram of distances between border points of *tbx16_j_* and *tbx16* 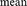 confirmed that most of the expression contours lay within two cell rows from each other (**Fig. S20c**). Note that this quality measure was an upper bound of the registration quality reflecting both the mapping variability and the intrinsic inter-embryo variability.

Additionally, we confirmed that the spatial relationships between every gene and the 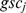 patterns in the analyzed embryos were the same in each template with respect to the 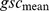 domain. In particular, this was the case for the *oep*-*gsc* pair illustrated in **Fig. 4c,d**.

### Analyzing a gene expression atlas with dedicated tools

Various analysis tools for the quantitative analysis of a spatiotemporal atlas of gene expression were also developed (see Materials and Methods). We performed an automated identification of gene coexpression pattern dynamics in space and time, explored clustering strategies at the cellular level to automatically identify morphogenetic domains or spatiotemporal gene synexpression groups, and introduced an “entropy” analysis for gene expression.

#### Coexpression dynamics

**Figure 5:**
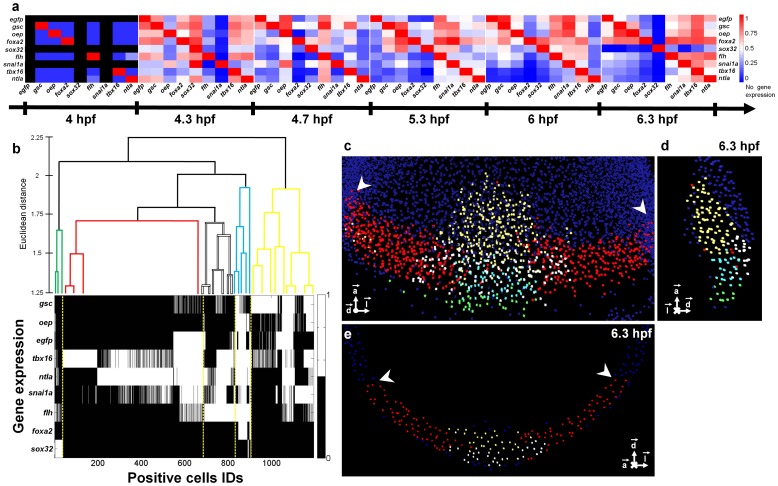
Assessing gene coexpression and cell genetic profiles. (**a**) Matrix displaying the percentage of cells coexpressing any given gene pair at developmental stages from 4 to 6.3 hpf (see also **Fig. S21**). Gene pairs were ordered according to the similarity of the evolution of their patterns over time (**Fig. S23**). (**b**) Spatial WPGMA clustering at 6.3 hpf of the 1,194 positive cells according to the similarity of their gene expression profiles. (**c**) Volume rendering, (**d**) lateral view and (**e**) coronal view of template nuclei at 6.3 hpf. Nuclei were classified according to their gene expression profiles, which revealed 5 distinct morphogenetic domains: dorsal hypoblast (yellow), marginal dorsal epiblast (blue), dorsal epiblast (white), paraxial and lateral blastoderm margin (red), forerunners and dorsal YSL (green). White arrowheads indicate the limits of the imaged analyzed embryos. Upper panel, sagittal section, lower panel equatorial section passing through the embryonic shield. Scale bar 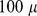
m.

Coexpression between gene patterns was systematically analyzed across the atlas for all 36 possible gene pairs and 6 developmental stages. At each time point, we measured the number of cells that expressed a given pair of genes with respect to the total number of positive cells for each of the pair components. This quantification was used to construct a coexpression matrix (**Fig. 5a**) and document the pairwise evolution of gene coexpression with unprecedented temporal and spatial resolution. Alternatively, the evolution over time of the topological relationships between two gene patterns, which could be identity, inclusion, exclusion or intersection, was displayed as a trajectory in 2D space (**Fig. S2**). For example, the *oep* and *sox32* domains went from inclusion at 4.3 hpf to intersection between 4.7 and 6 hpf to complete exclusion by 6.3 hpf. This representation highlighted as well the similarity of the *gsc* and *egfp* patterns until early gastrulation, a feature also captured by the high values of Dice's coefficient, 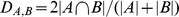 (**Fig. S22**). The *gsc*-*egfp* pair achieved an average 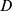 value of 0.77 over time, with a standard deviation of 0.1, validating the transgenic line as an acceptable reporter of the *gsc* activity at these developmental stages.

#### Morphogenetic domain clustering

At any given time step, we searched for potential morphogenetic domains using 3D clustering of the cells 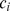 according to their gene expression profile, 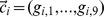, without any a priori assumption about their spatial location (**Fig. 5b**). At 6.3 hpf, the classification of the 1,194 positive cells resulted in the identification of 5 spatial domains with specific morphological locations associated to their particular gene expression profiles (**Fig. 5b–e** and **Movie S4**). This clustering strategy revealed the antero-posterior and medio-lateral patterning of the mesendodermal tissue at the onset of gastrulation.

#### Synexpression groups

The identification of synexpression groups, i.e. genes with potentially the same spatial and temporal regulation of expression, was automated by hierarchically clustering the genes 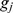 analyzed in the atlas according to the spatiotemporal similarity of their expression pattern at the single cell level, 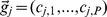. The 9 genes used in our atlas (*egfp*, *gsc*, *oep*, *foxa2*, *sox32*, *flh*, *snai1a*, *tbx16*, *ntla*) were clustered into 5 synexpression groups compatible with previous biological descriptions [31] (**Fig. S23**).

#### Gene expression entropy

We also measured the Shannon entropy of gene expression. In our atlas of 9 genes, where each cell expresses one of 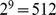 possible gene expression profiles, the entropy cannot be greater than 9 bits per cell (bpc). The entropy that we measured increased rapidly from 2.2 bpc at 4 hpf to a maximum of 5.7 bpc at 4.7 hpf, then slightly decreased to 5.1 bpc at 6.3 hpf (**Fig. S24a**). The increase in entropy may be related with the progressive increase in the number of non coexpressed genes (**Fig. S24b**). In addition, we measured the contribution of each gene expression profile to the global entropy. During the analyzed period, regardless of the time step, only around 100 different gene profiles were expressed out of the possible 512, and only a small number of gene profiles, between 30 and 50, were found to be responsible for 75% of the whole entropy (**Fig. S24c–d**).

Finally, we demonstrated that the proposed clustering and entropy schemes were robust against changes in the threshold values used to segment the gene expression patterns in the atlas (**Fig. S25**). In particular, we chose two genes in the atlas at 6.3 hpf: *oep* (coexpressed with *gsc*) and *ntla* (expressed in a larger area than *gsc*). For these two expression patterns, we varied the thresholds chosen by the biologist expert by 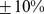 and computed the new segmented patterns, which modified accordingly the number of positive cells found in the atlas. The resulting entropy was almost the same as before.

## Discussion

We have designed, developed and delivered the Match-IT and Atlas-IT software tools dedicated to the reconstruction, analysis and visualization of a 4D atlas of gene expression in zebrafish early embryogenesis. The atlas comprises 6 different time points between 4 and 6.3 hpf, gathering data for 9 gene patterns into 6 different 3D templates.

So far, the only known method delivered for the reconstruction of gene expression atlases in the zebrafish was designed by Ronneberger et al. [21] for the brain and at late developmental stages, when a large number of morphological landmarks could already be recognized. Given the complexity of building a zebrafish brain atlas at late stages, the authors imposed strong constraints on the data in terms of staining protocols and imaging. Our own atlasing strategy was designed to map partial 3D volumes onto whole embryos chosen as templates. Specimens were only required to display, in addition to any pattern of interest, nuclear staining for single-cell counterstain and a common gene expression pattern, *gsc* in the present version of the atlas, used for the registration step. This gene was chosen as a relevant marker, with early, strong and well-regionalized expression, to serve as a reference for constructing the dorsal side's gastrulation atlas. Thus we have minimal prerequisites for data format and specimen preparation, which should facilitate the introduction of new data into the atlas. In addition, our scheme could be easily adapted to other vertebrate organisms, e.g. xenopus, dogfish or lamprey, at early stages of development, when too few morphological features are available to use landmark-based registration methods in the mapping process. The possibility of visual inspection and, if necessary, manual correction using our Match-IT graphical interface contributes to flexibility and accuracy when integrating new data into the atlas and validating the results.

### Resolution at the cellular scale

Our choice to work with a hybrid automated/supervised method of nuclear center detection proved to be suitable for quantifying certain features of gene expression pattern dynamics at the cellular level. This opens the possibility to discuss, in terms of cell number, the overlap between gene expression patterns and their evolution in time. It also allows studying whether cell proliferation alone is enough to account for the expansion of gene expression patterns, by correlating internuclear distance and cell division, which, in zebrafish early development, happens at constant global cell volume (**Fig. S26**). On the other hand, the resolution of the atlas at the cellular scale is a requirement to exploit the correlation between gene expression dynamics and cell lineage. Cellular resolution enables further mapping of the atlas onto digital specimens reconstructed from live *in toto* imaging, starting with our transgenic line.

Working at the cellular resolution was also intended to tackle the problem of gene expression quantification. Current strategies for *in situ* hybridization could at best provide relative measurements suitable for quantifying graded patterns and fuzzy borders within each analyzed embryo. Such a relative quantification would be readily available from our atlas (**Fig. S10**). We expect future developments of the programmable *in situ* amplification technique [7] to help achieve quantification of gene expression comparable among different analyzed embryos at the cellular level.

### Individual variability and the atlas

The relevance of the atlas relies on its ability to represent and integrate the same information as would be obtained by inspecting different patterns in the same specimen. This depends on the accuracy of the registration strategies but most importantly on how the atlas construction scheme deals with individual variability. Every step of the mapping strategy has to cope with individual variations in terms of shape, cell number, cell density, and variability of the reference gene pattern. In this context, the choice of the template is crucial. The template should be closest to the mean of the population, based on geometric parameters and gene expression. Ideally, a multiscale model of individual variability should drive the choice of the atlas template as well as representative reference patterns or features to guide the mapping. In our case, the *gsc* pattern served as a guide for the registration step, based on the hypothesis that its expression is symmetric with respect to the bilateral plane. Although this is a reasonable assumption, it is an approximation that might be confronted to other features such as other reference gene patterns or additional morphological traits. The templates used in this paper were visually chosen to be the closest to the mean. Although this choice may not be fully representative of the average morphology, the concept of average is also not completely relevant for the released proof-of-principle atlas that comprises 9 specimens per developmental stage. The tools released here open the way for a broader population that could ideally produce a more representative template.

In this context, we calculated a mean *gsc* expression pattern after registering the domains from 9 different specimens. The resulting 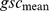 domain could be subsequently used as a new reference to refine the global mappings. Moreover, all the genes gathered in the atlas could be averaged, thus preventing potentially misleading conclusions based on single specimens that might be outliers. The increase in size of the cohorts will allow exploring the possible convergence of the averaging strategy toward a single or multiple prototypical specimens.

### Tools for analyzing the atlas data

Atlas resources will only be fully exploited with the development and use of automated analysis methods and dedicated visualization tools. Toward this objective, we designed Atlas-IT to provide a number of functionalities not available in any of the visualization tools that we examined: augment/visualize/analyze raw data and segmented data, calculate mean gene expression domains, gene coexpression patterns, synexpression groups, and morphogenetic domains by cell clustering. Interactive visualization and data display are essential to reveal biologically relevant information. The exploration of analytical methods to highlight spatial and temporal correlations is also a major endeavor. Typically, clustering methods have been used to establish the gene expression profiles of cells and tissues from microarray data, and more recently to group anatomical regions according to their gene expression profile [13],[32],[33]. Although clustering of spatial gene expression patterns has been described elsewhere [34], it is the first time that this method is applied to gene expression profiles at the cellular level, 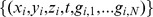, providing the means to reveal morphogenetic domains and synexpression groups. Additionally, whereas the Shannon entropy has been used to measure gene expression complexity [35], it is also the first time that this measure is applied to spatially mapped data. Introducing the concept of “genetic entropy” in the analysis of atlas data offers a new systematic way to assess cell diversification and its underlying genetic complexity. This analysis proved to be robust against the noise due to errors in the segmentation and/or spatial mapping. Although a relatively high proportion (100 out of 512) of all possible gene expression profiles were found in the atlas, only 30 of them (i.e. 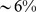) produced 75% of all the atlas genetic information (**Fig. S24**).

### Conclusion

Making a gene expression atlas is a necessary step toward the integration of multiscale and multimodal data, which should be organized, displayed and annotated to provide and share as much relevant information as possible. Developmental biology remains far behind the biomedical field in the construction and sharing of this type of resources. Thus, before reaching a consensus and establishing standards in the field, a lot remains to be explored in terms of different schemes, their flexibility, their potential and limitations. The atlas construction process presented here allowed us to address some of the most difficult biological questions linked to individual variability, its components and characteristic scales. A gene expression atlas often comprises hundreds or even thousands of genes [36]. On the other hand, resources can grow and diffuse only if deployed together with appropriate algorithms and analytical tools. Our novel construction and manipulation methods, which led to the first release of the zebrafish blastula and early gastrula atlas, are meant as a contribution toward the complete reconstruction of the zebrafish embryonic physiome (or “embryome”) under different genetic and environmental conditions.

## Materials and Methods

### In situ hybridization

*In vitro* fertilization was used to synchronize the spawn from wild type (wt) or transgenic crosses from the custom made fish line Tg(−4gsc:egfp)isc3. Embryos, staged according to Kimmel et al. [37], were fixed 24 h at 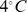 in PFA 4% then rinsed 3 times in PBS 0.1% Tween and stored at 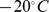 in ethanol. Double fluorescent in situ hybridization (FISH) was carried out as described in Brend et al. [38] using antisense RNA probes labeled with fluorescein or digoxygenin. Probes were detected with an anti-digoxigenin-POD Fab fragment and anti-fluorescein-POD Fab fragment (Roche) used at 1∶250 in a blocking reagent solution (Roche). Probe detection was done with Cy3 or Cy5 mono NHS ester (Amersham) or NHSFluoresceine (Pierce) tyramides as POD substrates. Nuclei were stained in DAPI (Invitrogen D3571).

### Image data acquisition

As an input, our methodology used 3D images acquired by confocal laser scanning microscopy from fixed zebrafish embryos with fluorescent staining of gene expression patterns and DAPI counterstain to highlight cell nuclei. Image acquisition was performed with a Leica SP2 two-photon (for DAPI) and confocal laser scanning upright microscope with a Leica objective HCX APO 20X/0,5W U-V-I or HCX APO 10X/0,3. Embryos were mounted in a teflon mold at the bottom of a 3 cm Petri dish filled with 1×PBS, 01% twin 20, and maintained properly oriented with 1% agarose.

The nuclei and *gsc* expression domains were systematically revealed in all the analyzed embryos and templates, and used to compute the gene expression mappings. In addition to the reference gene, *gsc*, each analyzed embryo was stained for the expression of another gene of interest. The template data was obtained by imaging the whole embryo with a 10× objective while the analyzed specimens were imaged with a 20× objective providing a 3D view limited to the dorsal side of the embryo with a better spatial resolution (**Fig. 2a,b** and **Movie S1**).

The fluorescent *in situ* hybridization used a state-of-the-art protocol [38] and reproduced standard data (zfin.orgzfin.org). More details about data acquisition parameters and specimen features can be found in **Table S1** and **Fig. S1, S2, S3, S4, S5, S6, S7**.

### Algorithmic details of Match-IT and Atlas-IT

The Match-IT custom-made code was implemented in ITK and Matlab, including the MathWorks package “geom3D” redistributed under a BSD license. A public release of this software, together with sample datasets and a user guide, accompanies the publication of this article, http://bioemergences.iscpif.fr/documents/MatchIT.zip. The segmentation of the gene expression patterns in each analyzed embryo was carried out by a thresholding operation supervised by a biologist to best define the domain features. This operation was followed by “morphological closing” [39], a mathematical transformation based on a spherical structuring element the size of a typical cell diameter (i.e. internuclear distance). Finally, a converse “morphological opening” operation left only the largest connected pattern. The common referential extraction started by applying a spherical fit to the outer cell nuclei in all analyzed embryos and templates. The blastoderm margin was identified with a plane, 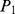, fitted to the 5% southernmost nuclei. The bilateral symmetry plane, 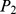, was found by connecting the spherical model center and the center of mass of the *gsc* segmented domain perpendicular to the blastoderm margin. The origin of the triplet was placed at the latitude of the blastoderm margin, and the longitude was defined by the center of mass of the *gsc* domain. The registration ([10],[40]) of the analyzed embryo images on the template employed the ITK registration toolkit to optimize the cross-correlation metric between the embryo shape of the template and that of the analyzed embryos according to a step gradient optimizer. The embryo shapes were weighted by the inverse distance function to the external blastoderm contour (i.e. half the average internuclear distance away from the outermost nuclear layer).

The Atlas-IT custom-made visualization platform was implemented in Processing. A public release of this software, together with sample datasets and a user guide, accompanies the publication of this article, http://bioemergences.iscpif.fr/documents/AtlasIT.zip

### Analysis tools for spatiotemporal gene expression atlases

#### 3D clustering of cells according to their gene expression profiles

Template cells at one given developmental stage were grouped according to the similarity of their gene expression profiles using a hierarchical clustering scheme. For a given observed time 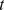, we associated a gene expression vector to each of the detected 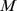 cells: 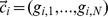, where 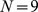 is the number of genes under study, 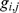 is 1 if the 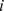-th cell expressed the 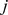-th gene, and is 0 otherwise. Then, a weighted pair group method with averaging (WPGMA) was performed on these vectors based on the Euclidean distance (**Fig. 5**). This method was implemented with the Statistics toolbox of Matlab.

#### 3D+time clustering of genes according to their spatiotemporal regions of expression

Here the analysis involved the totality of cells across all acquisition times. For each of the 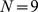 genes under study, we associated a spatiotemporal expression vector: 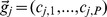, where 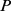 is the total number of cells observed across all the observation times: 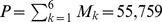, with 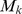 being the number of cells observed at the 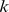-th time of acquisition. For the 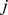-th gene, the 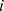-th coordinate of that vector, 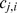, was set to 1 if the 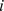-th cell expressed that gene, and 0 otherwise. Clustering the genes according to their associated region of expression vectors allowed identifying synexpression groups [41]. We used the same WPGMA algorithm based on the Euclidean distance and Matlab implementation (**Fig. S23**).

#### Shannon entropy of gene expression

We used information theory to measure an “entropy” for gene expression. We consider that the gene expression 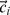 observed in the 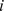-th cell (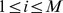, where 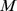 is the number of cells) is the value taken by a discrete random variable 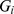 among all possible N-uples 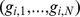 of 0's and 1's, i.e. all the integers in the interval 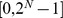. Assuming that the random variables 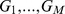 are independent and identically distributed, e.g. with the same law as a given random variable 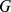, their common entropy is: 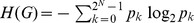, with the usual conventions that 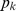 denotes the probability of event 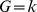 and we set 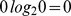, where 2 was chosen as the base in order to express the result in bits. Each 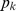 can be estimated from the observed sample 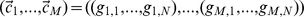 by setting 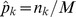, where 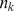 is the number of template cells showing the 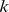-th expression N-uple. Replacing each 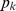 in the formula by the corresponding 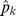 gives an estimate for 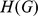. Under the same hypotheses the total entropy for the population of 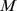 cells is equal to 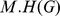, but if the 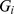 are not independent, the total entropy of the population (defined using a single random variable to generate the combined expressions of all the cells in a population, and requiring the observation of several populations to permit estimation) can be less than 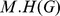.

### Computation of the mean *gsc* expression domain

At each developmental time point, a total of 9 different analyzed embryos with 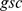 staining were mapped onto the template where 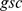 expression was also revealed. Consequently, every nucleus, 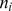, in the template was assigned a value, 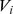, ranging from 0 to 9, depending on the number of analyzed patterns that led to its selection as positive for the expression of 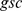. We used a Voronoi diagram to model the cell around each nucleus and assigned these cells their corresponding value 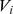. In order to measure the variability of the resulting mean 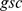 expression, we studied the profile of 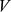 across 3 cutting lines centered on the mean 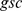 centroid and following the specimen anatomy along the lateral, radial and sagittal directions respectively (**Fig. S16**).

### Evaluation of the entropy and clustering robustness with respect to gene segmentation thresholds

We demonstrated that the proposed clustering and entropy schemes are robust against changes in the thresholds employed to segment the gene expression patterns in the atlas. In particular, we chose two gene expressions in the atlas at 6.3 hpf: 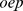, which co-expresses with 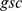, and 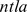, which spreads through a much larger area than 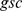. For the expression of these two genes, we modified by 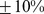 the thresholds chosen by the biologist expert, computed the new segmented patterns and modified accordingly the number of positive cells found in the atlas. The entropy and clustering resulting from these modified atlases were compared to the original atlas and showed to be robust against these threshold changes (**Fig. S25**). To compare the modified vs. the original clustering (**Fig. 5b–c**) we used two metrics previously employed in literature: a) the correlation between the distance matrix that generate the modified and the original clustering hierarchical trees [42], b) the cophenetic correlation, a measure of how faithfully a hierarchical tree preserves the pairwise distances between the original data points [43], [44]. In this later case, the cophenetic coefficient was extracted by comparing the original hierarchical tree to the new pairwise distances generated by the modified atlases. To compare the modified vs. the original entropy we computed the difference in number of bits. The biggest difference between all the modified and the original atlas was 0.15 bits in entropy and a 0.03 decrease for both the cophenetic coefficient and the correlation between distance matrix (**Fig. S25**). To put these values in perspective, the minimal possible variation to the atlas (changing the value of one gene expression for one cell only) had an impact of 0.0003 bits of entropy and 0.005 in cophenetic correlation, whereas a substantial variation to the atlas (e.g. changing one third of the atlas values or substituting it by a random atlas) had an impact of 0.83 and 3.6 bits of entropy and 0.52 and 0.79 in cophenetic correlation respectively.

## Supporting Information

Figure S1 **Raw data rendering of** ***sox32*** **expression pattern.** For every developmental stage, four panels are displayed: Top right: *gsc* expression (red), bottom right: *sox32* (green), top left: *gsc* and *sox32* expressions viewed from the dorsal side, bottom left: *gsc* and *sox32* expressions viewed from the ventral side. The analyzed embryo's nuclei are shown in gray.
(TIF)

Figure S2 **Raw data rendering of** ***tbx16*** **expression pattern.** For every developmental stage, except at 4 hpf when there is no *tbx16* expression, four panels are displayed: Top right: *gsc* expression (red), bottom right: *tbx16* (green), top left: *gsc* and *tbx16* expressions viewed from the dorsal side, bottom left: *gsc* and *tbx16* expressions viewed from the ventral side. The analyzed embryo's nuclei are shown in gray.
(TIF)

Figure S3 **Raw data rendering of** ***oep*** **expression pattern.** For every developmental stage, four panels are displayed: Top right: *gsc* expression (red), bottom right: *oep* (green), top left: *gsc* and *oep* expressions viewed from the dorsal side, bottom left: *gsc* and *oep* expressions viewed from the ventral side. The analyzed embryo's nuclei are shown in gray.
(TIF)

Figure S4 **Raw data rendering of** ***snai1a*** **expression pattern.** For every developmental stage, except at 4 hpf when there is no *snai1a* expression, four panels are displayed: Top right: *gsc* expression (red), bottom right: *snai1a* (green), top left: *gsc* and *snai1a* expressions viewed from the dorsal side, bottom left: *gsc* and *snai1a* expressions viewed from the ventral side. The analyzed embryo's nuclei are shown in gray.
(TIF)

Figure S5 **Raw data rendering of** ***foxa2*** **expression pattern.** For every developmental stage, four panels are displayed: Top right: *gsc* expression (red), bottom right: *foxa2* (green), top left: *gsc* and *foxa2* expressions viewed from the dorsal side, bottom left: *gsc* and *foxa2* expressions viewed from the ventral side. The analyzed embryo's nuclei are shown in gray.
(TIF)

Figure S6 **Raw data rendering of** ***ntla*** **expression pattern.** For every developmental stage, four panels are displayed: Top right: *gsc* (red) and *ntla* (green) expressions viewed from the dorsal side, bottom right: *gsc* and *ntla* expressions viewed from the ventral side, top left: *gsc* expression, bottom left: *ntla* expression. The analyzed embryo's nuclei are shown in gray.
(TIF)

Figure S7 **Raw data rendering of** ***flh*** **expression pattern.** For every developmental stage, four panels are displayed: Top right: *gsc* (red) and *flh* (green) expressions viewed from the dorsal side, bottom right: *gsc* and *flh* expressions viewed from the ventral side, top left: *gsc* expression, bottom left: *flh* expression. The analyzed embryo's nuclei are shown in gray.
(TIF)

Figure S8 **Raw data of one analyzed embryo mapped onto the template at 6.3 hpf.** (**a**) Raw data rendering of one analyzed embryo showing the nuclei (gray), *gsc* expression (red) and *tbx16* expression at 6.3 hpf. (**b**) Raw data rendering of the 3D template showing the nuclei (blue) and *gsc* expression (orange) at 6.3 hpf. (**c**) Analyzed embryo's nuclei (gray) and *tbx16* expression (green) are showed superimposed on the template's nuclei (blue) after they were mapped with Match-IT.
(TIF)

Figure S9
**Evaluation of nuclear center detection.** (**a**) Volume rendering of nuclear raw data (blue) together with the manually labeled “ground truth” (GT, red) for one analyzed embryo dataset. (**b**) Centers produced by our methodology (visually interactive choice of the optimal parameters by an expert) compared to the manually labeled GT. Out of the 689 cells in GT, there were 664 correct detections (blue), 4 false positives (FP, red), and 25 false negatives (FN, yellow), with a resulting error rate of 4.2% (
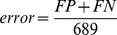
). The chosen parameters were a 7% threshold, and 2.8 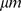 and 12 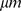 standard deviations for the two Gaussian kernels. A detection was considered correct when lying less than 4.2 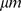 (i.e. the approximate radius of the smallest nucleus in the dataset) from GT. (**c**) Centers produced by decreasing the detection threshold to 6%. As a consequence, the number of FN (yellow) is reduced to 20 at the cost of raising the number of FP (red) to 14 with a resulting error rate of 4.9%. (**d**) Centers yielded by increasing the detection threshold chosen by the expert to 8%. As a consequence, the number of FP (red) is reduced to 1 at the cost of raising the number of FN (yellow) to 40 with a resulting error rate of 5.9%. Scale bar 50 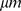. (**e**) Variations of the error rate with respect to changes in the threshold and standard deviations. Blind results obtained by an expert following our methodology (red square) showed to be robust against possible variations around the selected parameters.(TIF)

Figure S10
**Gene expression quantification.** 3D rendering of the relative gene expression levels measured at the cellular scale for *tbx16* at 6.3 hpf. The gene expression levels range from 0 (dark blue) to 1 (red). Centered in each nucleus, a sphere with a radius equal to the average internuclear distance was used to measure the mean intensity values of the raw *tbx16* expression. The mean background intensity, measured in the image regions outside the embryo, was subtracted from these values, which were also compensated by a depth penetration factor computed from the attenuation observed on the nucleus channel. Scale bar 50 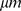.(TIF)

Figure S11
**Evolution of the internuclear distance over time.** The mean internuclear distance (in 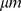) is calculated for 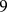 different specimens at each stage. The observed decrease fits with an average of approximately 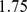 divisions per cell between 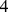 and 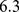 *hpf* and an exponential decrease in the cell cycle length. This is in agreement with previous observations in literature and validates the accuracy of the center detection procedure. Standard deviation is interpreted as reflecting individual variations.(TIF)

Figure S12 **Quantification of the morphological variability among individual embryos.** In 95% of the matched individuals, the radial size differs by less than 10% from the mean radius at each developmental stage.
(TIF)

Figure S13
**Positive cell selection in the template.** (**a**) Raw *tbx16* expression pattern (green) from the analyzed embryo mapped onto the template's raw nuclei (blue). (**b**) Template nuclei (blue) falling into the analyzed gene expression domain are considered positive (green). White arrowheads indicate the limits of the imaged analyzed embryo. Scale bar 100 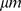.(TIF)

Figure S14
**Variation in the number of positive cells between analyzed embryos and template as a function of their relative internuclear distance.** More than 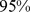 of the analyzed embryos fall within a 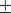 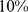 deviation from the identity function, yielding a statistical p-value of 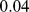. The two specimens deviating from this norm in the plot come from a very early developmental stage, 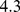 *hpf*, when staging is more difficult due to the lack of morphological traits.(TIF)

Figure S15
**Evolution of the** ***egfp*** **-** ***gsc*** **pair through time after being mapped onto the template.** Atlas-IT interface displaying the template nuclei (dark blue) and the coexpression (yellow) between the *gsc* 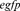 and *egfp* expressions. Scale bar 100 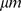.(TIF)

Figure S16
**Variability of the** ***gsc*** **gene expression pattern.** (**a**) Volume rendering of the aggregated *gsc* expressions, *gsc* 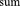, together with the three cutting lines along which variability is measured: *lateral line* (red), *radial line* (blue) and *sagittal line* (green). The black arrowhead indicates the *gsc* 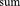 centroid. (**b–f**) *Left panel:* From left to right: equatorial, sagittal and dorsal orthoslices passing through the *gsc* 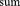 expression domain at the level of its centroid. The color code indicates the number of *gsc* expression repetitions in the template cells based on the analysis of the 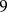 available specimens. Right panel: Profile showing how many embryos (out of the 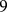 mapped individuals) expressed *gsc* along the three cutting lines centered at the *gsc* 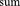 centroid as displayed in (a). Expression variability appears as additional rows of cells around a core domain (where cells are positive for all the observed specimens). The dotted lines indicate the borders of the *gsc* 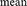 pattern.(TIF)

Figure S17
**Quantification of pattern differences: evaluation of** ***gsc*** **expression variance after mapping.** Each of the individual *gsc* segmented domains mapped onto the atlas were compared to the mean *gsc* following a leave-one-out strategy at every developmental stage between 4.3 and 6.3 *hpf*. The error bars represent the mean and the standard deviation of the distance, in 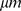, between the individuals' *gsc* borders and their corresponding *gsc* 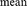. The average internuclear distance (dotted red line) ranges from 14 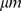 at 4.3 *hpf* down to 10 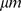 at 6.3 *hpf* (see **Fig. S11a**). As discussed for *Drosophila* embryos, individual variations in gene expression patterns, in terms of positive cell numbers or domain topology, could arise from gene expression regulation itself, and from geometric variations such as embryo size and cell proliferation rate variability. In the zebrafish early embryo, overall size, internuclear distance, and cell proliferation rate are dependent parameters (**Fig. S11** and **Fig. S26b**). Internuclear distance, expected to decrease through cell divisions until the end of gastrulation (10 hpf), was indeed variable among specimens, but converged toward similar values (**Fig. S11a**). There was however no clear correlation between embryo size and internuclear distance, possibly indicating variability in the proliferation rate and/or developmental speed of our batches of embryos. Because of the difficulty to separate the different components of variability, our atlasing strategy did not attempt to minimize it but introduced the calculation of mean expression domains (**Fig. S16**).(TIF)

Figure S18
**Quantification of pattern differences on the main embryo planes.** Mean and standard deviation of the distance, in 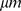, between the individuals' *gsc* borders and their corresponding *gsc* 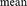 at 6.3 hpf. Distances were obtained using the expression contours in 3D (as performed in **Fig. 17**) and restricting them to the three main embryo planes: equatorial, sagittal and dorsal (see **Fig. 4b** and **Fig. S16a**).(TIF)

Figure S19
**Quantification of pattern differences along the domain borders.** For each developmental stage: histogram of the Hausdorff distances from the all the points placed at the complete outer border of each individual's *gsc* expression to the closest boundary point of the *gsc* 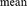 pattern. The vast majority of these boundaries is within two cell rows from *gsc* 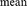 (cell diameter is 12 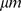). This constitutes an upper bound for the registration quality, as it reflects the variability in the mapping procedure plus the intrinsic interembryo variability.(TIF)

Figure S20
**Quantification of pattern differences: evaluation of** ***tbx16*** **expression variance after mapping.** (**a**) Each of the individual *tbx16* segmented domains mapped onto the atlas were compared to the mean *tbx16* following a leave-one-out strategy at 6.3 *hpf*. (**b**) Mean and standard deviation of the distance, in 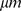, between the individuals' *tbx16* borders and the *tbx16* 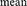 pattern. The average internuclear distance (dotted red line) is 10.3 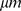 at 6.3 *hpf* (see **Fig. S11a**). (**c**) Histograms of the distance, in 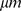, between the points located at individual *tbx16* borders to the closest point at the *tbx16* 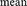 border.(TIF)

Figure S21
**A synthetic view of gene coexpression pairs and their evolution through time.** (**a**) Gene coexpression pairs fell into 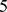 possible categories defined by gene pattern similarity: 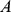 and 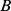 expression domains exclude each other (bottom left), 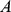 is included in 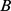 (bottom right), 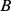 is included in 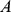 (top left), 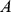 is identical to 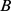 (top right), 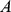 and 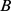 domains partially overlap (center). (**b**) This chart allows a visualization of the segregation of *oep*-*sox32* coexpression through time. (**c**) Gene pattern relationships and their evolution in time for the 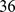 possible pairs. Coherence with a priori knowledge has been checked and demonstrates the power of the atlas construction strategy and further analysis tools.(TIF)

Figure S22
**Evolution of similarity coefficient for all possible gene pairs.** Dice's similarity coefficient: 
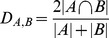
 was calculated for the 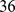 gene pairs, which were then arranged in descending order according to 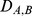 at 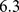 *hpf*.(TIF)

Figure S23 **Gene synexpression groups defined by their spatiotemporal clustering patterns.** (**a**) A hierarchical clustering of genes according to the similarity of their spatiotemporal regions of expression defined 5 different groups with characteristic spatiotemporal behaviors. For each group, a color code (column to the right of the panel) was displayed to indicate whether cells expressed 0, 1, 2 or 3 genes. The 8 analyzed genes fell into the following synexpression groups: *gsc-oep-foxa2*, *sox32*, *flh*, *snail1a*, *ntla-tbx16*. (**b**) Visualization of the synexpression groups identified in (a). Arrowheads indicate the limits of the imaged volume in the analyzed embryos.
(TIF)

Figure S24
**Gene expression entropy.** (**a**) Gene expression entropy as a function of time: the Shannon entropy provides a measurement of the complexity of a cell's gene expression profile. (**b**) Percentage of positive cells for each gene expression as a function of time. A gene expression (inhibited until a certain time step) that would suddenly start expressing would make the entropy increase by 1 bit at most. (**c**) Quantity of information (in bits) contributed by each gene expression profile at each time step. Expression profiles are sorted by decreasing contribution to the information. Only the first 150 profiles are plotted. We can observe that many of the possible 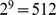 gene expression profiles are actually never used, and most of the information is conveyed by a small number (around 100) of representative combinations. (**d**) Number of gene expression profiles required to convey 60% (red line), 75% (green line) and 90% (blue line) of the total entropy at each time step. The ascending slopes from 4.0 to 5.3 hpf are compatible with the time trend toward more equidistribution visible in (c).(TIF)

Figure S25
**Robustness of entropy and clustering with respect to gene segmentation thresholds.** (**a**) From left to right: correlation between the distance matrix, cophenetic coefficients and entropy of the original and modified atlases. Threshold modifications in the expressions of *oep* and *ntla* (labeled ‘*oep*-10%’, ‘*oep*+10%’, ‘*ntla*-10%’, ‘*ntla*+10%’) showed metrics similar to the original atlas (labeled ‘atlas’) or a minimally modified atlas (labeled ‘one-cell’), and are all grouped around one value (green rectangle). They are clearly distinct from other, severe modifications in the atlas, such as substituting one third of its values (labeled ‘one-third’) or using a randomly generated atlas (labeled ‘random’). (**b**) Original cell values for *ntla* (left) and *oep* (right) in the atlas at 6.3 hpf (as seen in **Fig. 5b**) are compared to the values obtained after modifying by 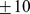
% the thresholds originally chosen by the biologist expert.(TIF)

Figure S26
**Correlation between cell proliferation and the expansion of gene expression domains.** (**a**) Evolution of the number of positive cells for each of the 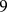 considered gene products. (**b**) Temporal evolution of the number of cells in the region of interest (ROI) centered on the dorsal side of each analyzed embryo as shown in (c). The cell proliferation rate extracted from this experiment matched previous observations from the literature. (**c**) Dorsal region of interest (ROI) used to measure the cell proliferation rate in each analyzed embryo. (**d**) Ratio between the increase rate of positive cells for a given gene and the estimated overall cell proliferation rate. This ratio indicates whether the dynamics of gene expression patterns can be explained by sustained expression in proliferating cells or requires upregulation (such as *egfp* by 6.3 *hpf*) or downregulation (such as *sox32* by 6.3 *hpf*).(TIF)

Movie S1
**3D rendering of the raw data from one analyzed embryo and the template used to construct the atlas model at 6.3 hpf.** Both analyzed embryo and template were imaged from the dorsal side but otherwise randomly oriented. After registration with Match-IT, the expression patterns stained in the analyzed embryo, *gsc* 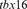 (red) and *tbx16* (green) are gathered in the atlas together with the *gsc* 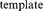 (orange). This movie is available as Supplementary Material to this paper and at http://bioemergences.iscpif.fr/documents/MovieS1-RawData.wmv.(MP4)

Movie S2 **Step-by-step procedure to map an** ***analyzed embryo*** **onto the atlas model with Match-IT.** This movie is available as Supplementary Material to this paper and at http://bioemergences.iscpif.fr/documents/MovieS2-MatchIT.wmv.
(MP4)

Movie S3 **Visualization and analysis of the final atlas model with Atlas-IT.** This movie is available as Supplementary Material to this paper and at http://bioemergences.iscpif.fr/documents/MovieS3-AtlasIT.wmv.
(MP4)

Movie S4 **Clustering of cells according to their gene expression profile.** See also **Fig. 3**. This movie is available as Supplementary Material to this paper and at http://bioemergences.iscpif.fr/documents/MovieS4-CellClusters.wmv.
(MP4)

Software S1 **Match-IT: A software package to map gene expression data at the cellular-scale onto an atlas model.** (**a**) Main window. **b**) Validation Graphical User Interface (GUI). A tutorial on using Match-IT can be found as an annex to this document. The Match-IT software package together with its tutorial and representative datasets can be downloaded from the Bioemergences website http://bioemergences.iscpif.fr/documents/MatchIT.zip.
(TIF)

Software S2 **Atlas-IT: A software package to visualize and analyze an atlas of gene expression at the cellular scale.** A tutorial on using Atlas-IT can be found as an annex to this document. The Atlas-IT software package together with its tutorial and representative datasets can be downloaded from the Bioemergences website http://bioemergences.iscpif.fr/documents/AtlasIT.zip.
(TIF)

Table S1 **Acquisition details of the early zebrafish microscopy datasets included in the atlas.** “Usage” column: a = included in the atlas, t = template. “Genotype” column: WT = wild type. “Detection” column: ISH = *in situ* hybridization.
(TIF)

User Guide S1 **Match-IT User Guide: A step-by-step protocol.** This user guide is available as Supplementary Material to this paper and at http://bioemergences.iscpif.fr/documents/MatchIT.zip.
(PDF)

User Guide S2 **Atlas-IT User Guide: A step-by-step protocol.** This user guide is available as Supplementary Material to this paper and at http://bioemergences.iscpif.fr/documents/AtlasIT.zip.
(PDF)
