# Supplementary figures and images for: A Digital Framework to Build, Visualize and Analyze a Gene Expression Atlas with Cellular Resolution in Zebrafish Early Embryogenesis
Source: PLoS Comput Biol. 2014 Jun 19;10(6):e1003670. doi: 10.1371/journal.pcbi.1003670 (PMC4063669; doi:10.1371/journal.pcbi.1003670)

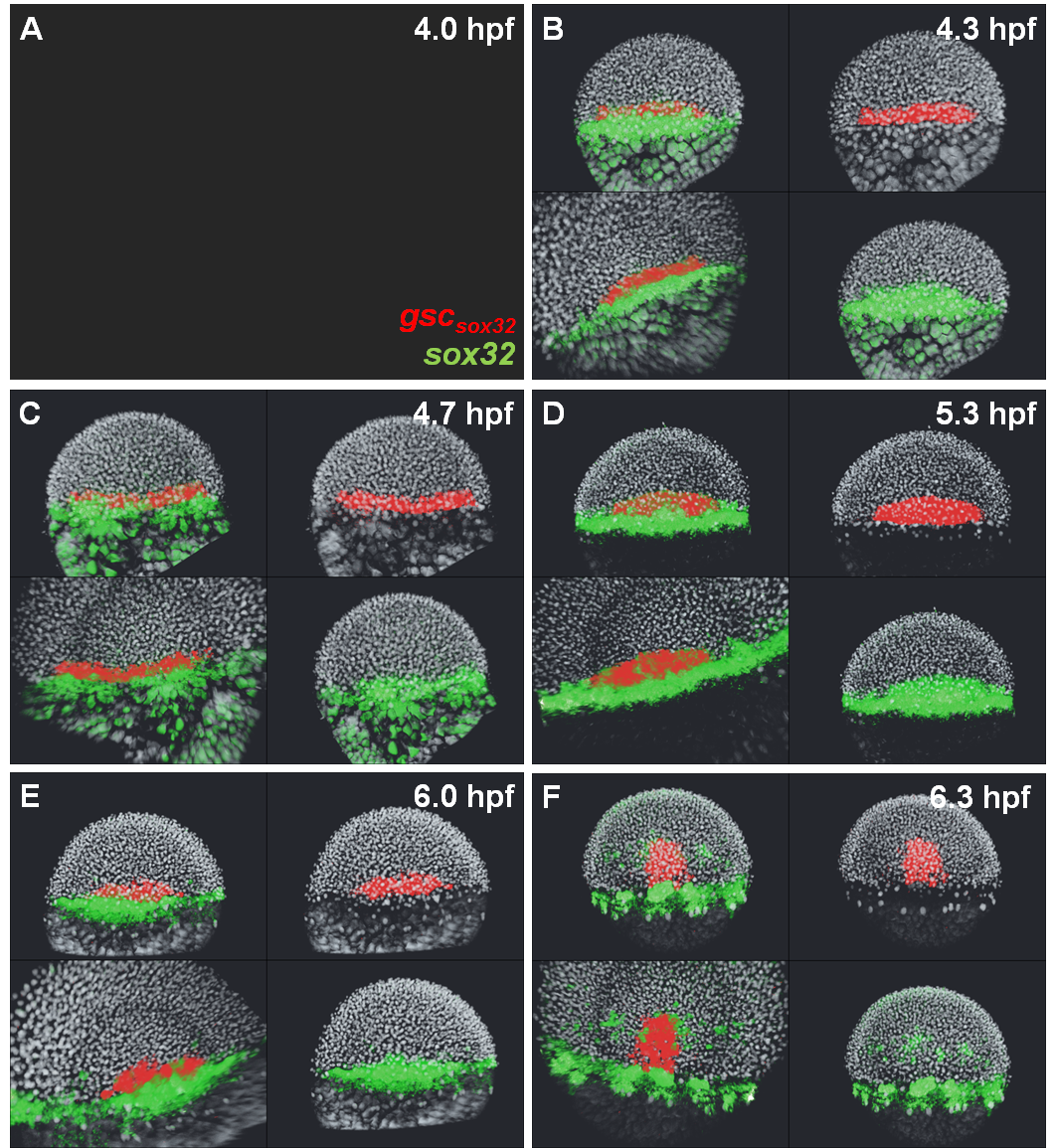

Supplement: Figure S1 — Raw data rendering of sox32 expression pattern. For every developmental stage, four panels are displayed: Top right: gsc expression (red), bottom right: sox32 (green), top left: gsc and sox32 expressions viewed from the dorsal side, bottom left: gsc and sox32 expressions viewed from the ventral side. The analyzed embryo's nuclei are shown in gray. (TIF) [file pcbi.1003670.s001.tif]

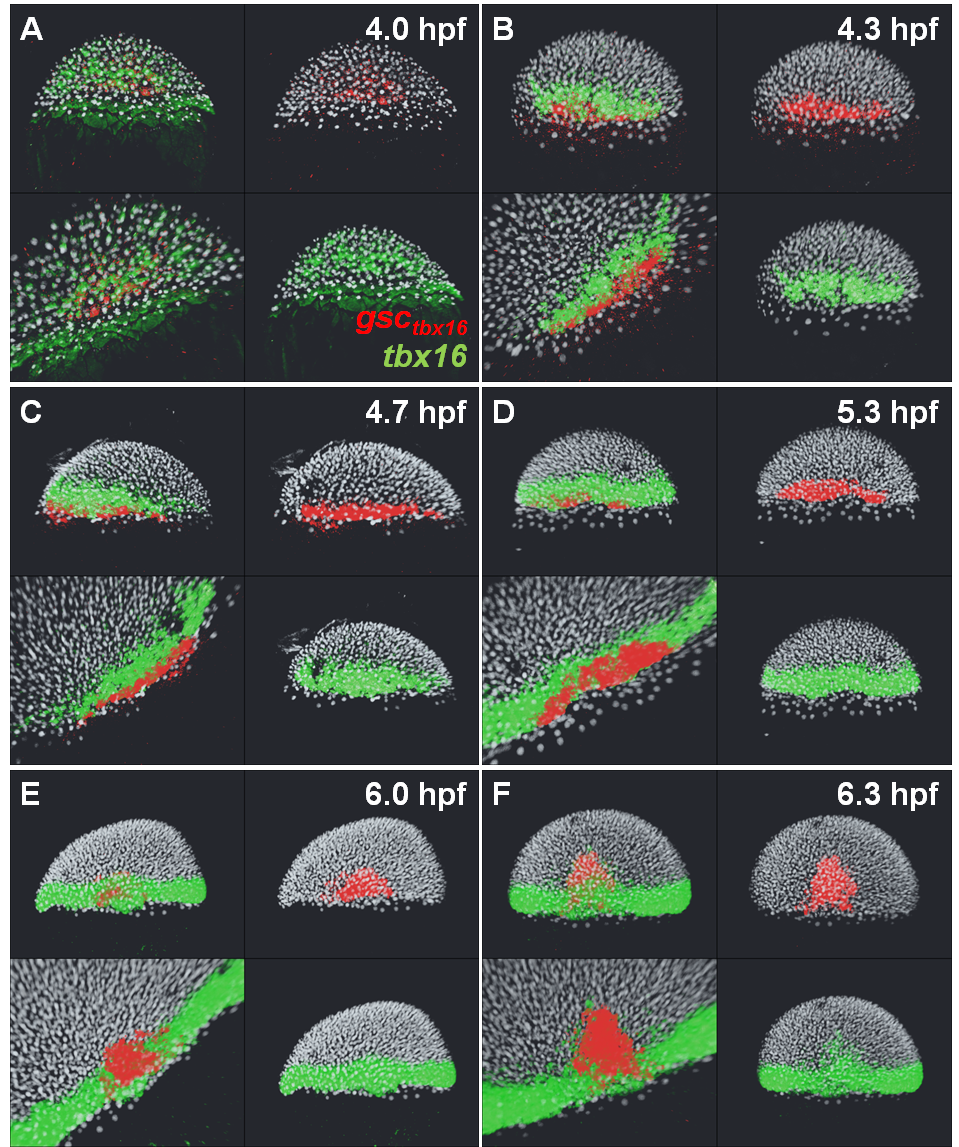

Supplement: Figure S2 — Raw data rendering of tbx16 expression pattern. For every developmental stage, except at 4 hpf when there is no tbx16 expression, four panels are displayed: Top right: gsc expression (red), bottom right: tbx16 (green), top left: gsc and tbx16 expressions viewed from the dorsal side, bottom left: gsc and tbx16 expressions viewed from the ventral side. The analyzed embryo's nuclei are shown in gray. (TIF) [file pcbi.1003670.s002.tif]

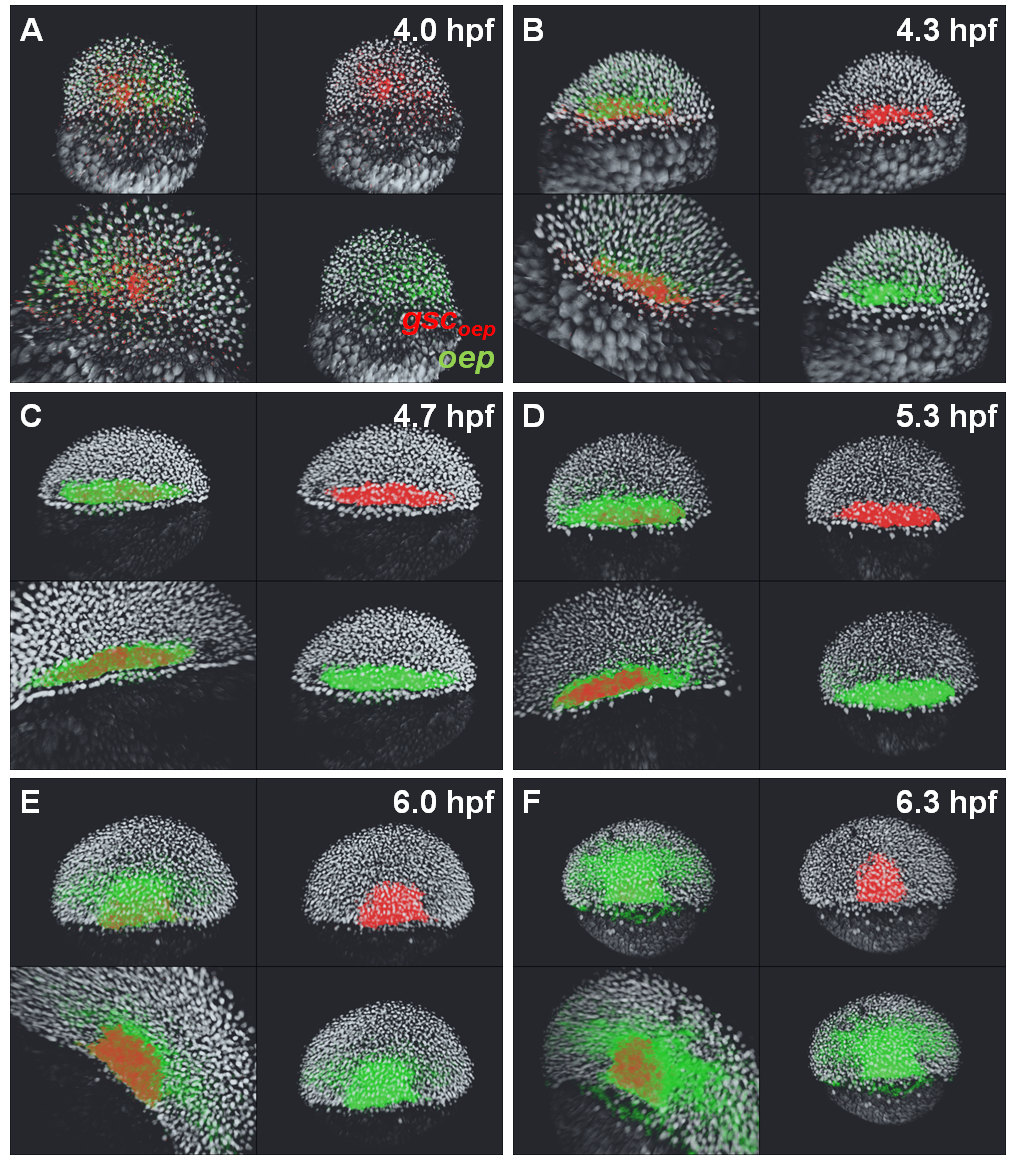

Supplement: Figure S3 — Raw data rendering of oep expression pattern. For every developmental stage, four panels are displayed: Top right: gsc expression (red), bottom right: oep (green), top left: gsc and oep expressions viewed from the dorsal side, bottom left: gsc and oep expressions viewed from the ventral side. The analyzed embryo's nuclei are shown in gray. (TIF) [file pcbi.1003670.s003.tif]

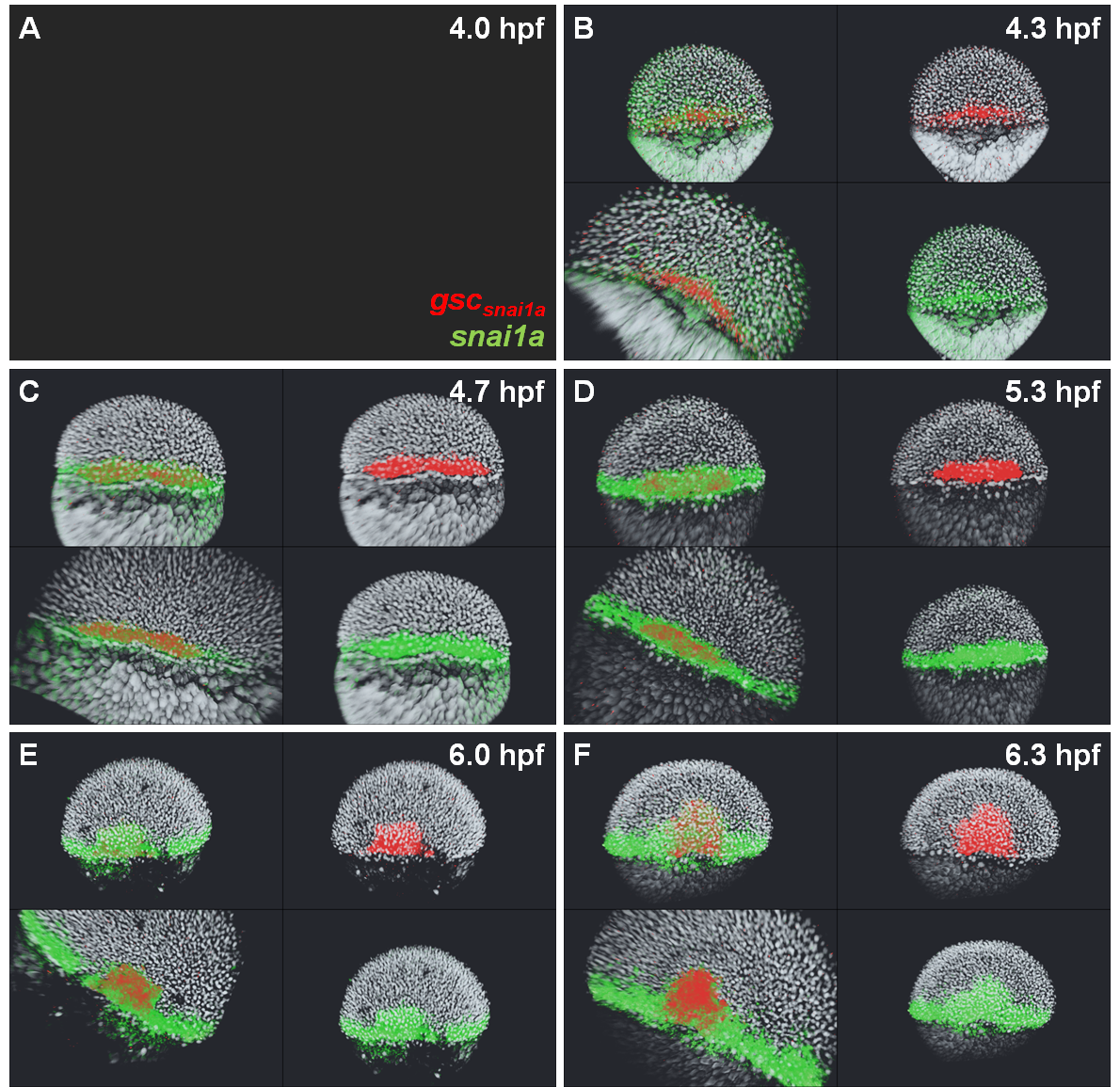

Supplement: Figure S4 — Raw data rendering of snai1a expression pattern. For every developmental stage, except at 4 hpf when there is no snai1a expression, four panels are displayed: Top right: gsc expression (red), bottom right: snai1a (green), top left: gsc and snai1a expressions viewed from the dorsal side, bottom left: gsc and snai1a expressions viewed from the ventral side. The analyzed embryo's nuclei are shown in gray. (TIF) [file pcbi.1003670.s004.tif]

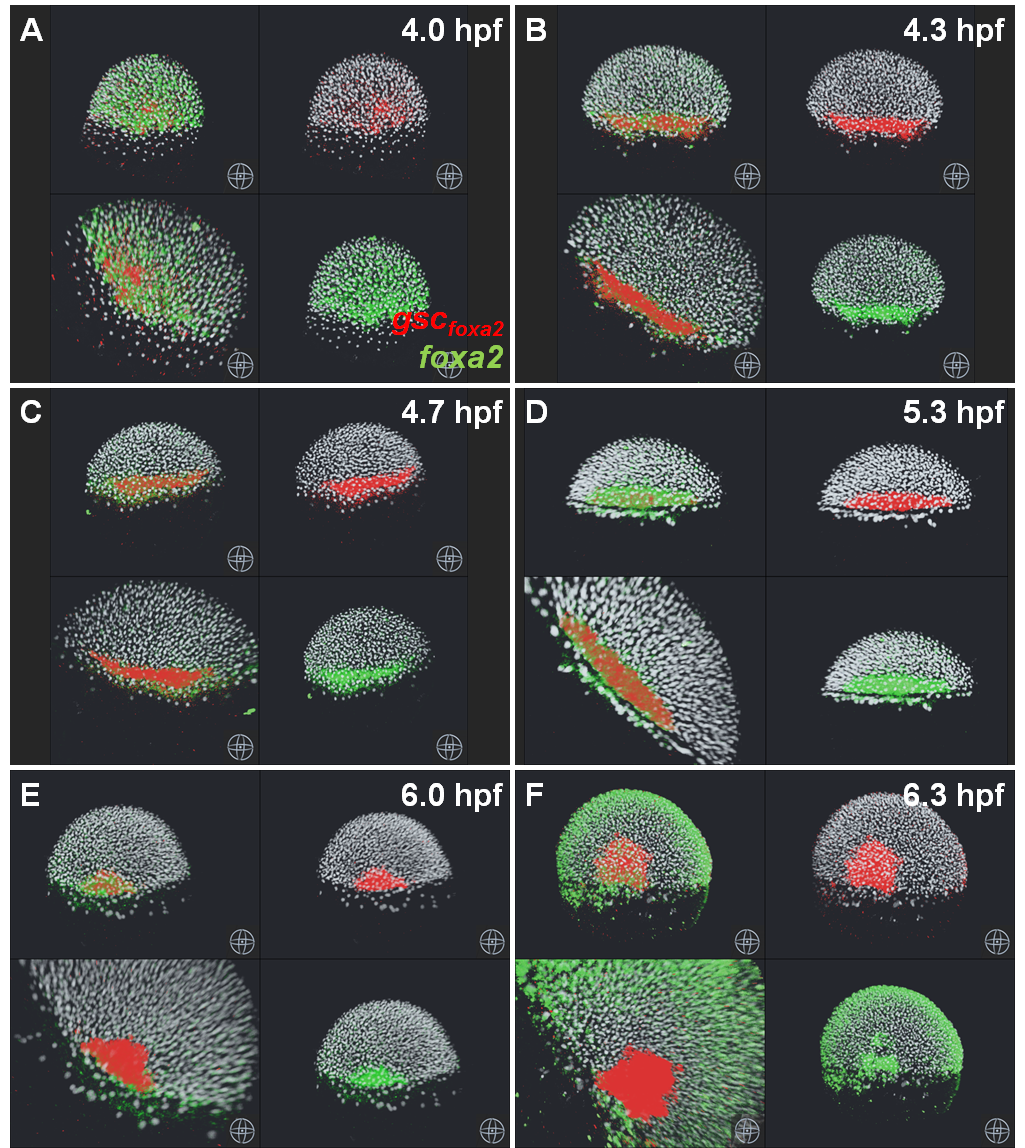

Supplement: Figure S5 — Raw data rendering of foxa2 expression pattern. For every developmental stage, four panels are displayed: Top right: gsc expression (red), bottom right: foxa2 (green), top left: gsc and foxa2 expressions viewed from the dorsal side, bottom left: gsc and foxa2 expressions viewed from the ventral side. The analyzed embryo's nuclei are shown in gray. (TIF) [file pcbi.1003670.s005.tif]

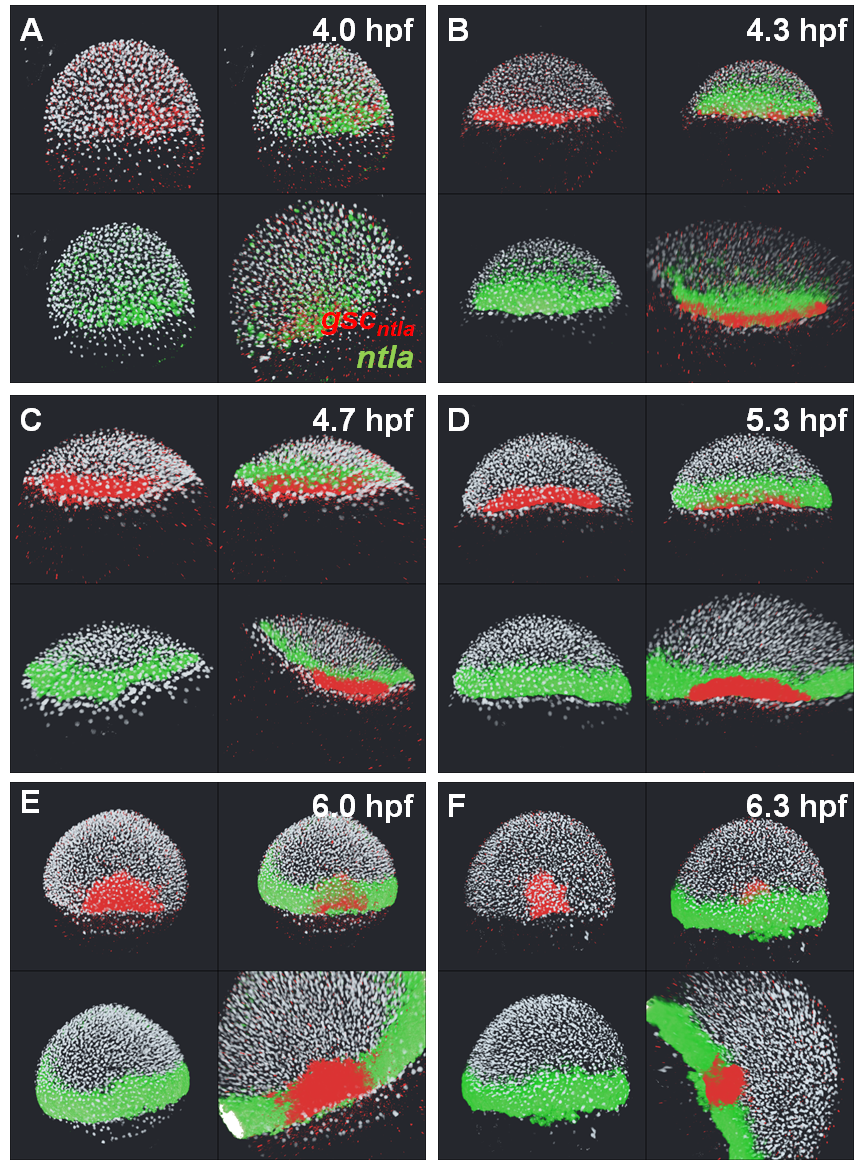

Supplement: Figure S6 — Raw data rendering of ntla expression pattern. For every developmental stage, four panels are displayed: Top right: gsc (red) and ntla (green) expressions viewed from the dorsal side, bottom right: gsc and ntla expressions viewed from the ventral side, top left: gsc expression, bottom left: ntla expression. The analyzed embryo's nuclei are shown in gray. (TIF) [file pcbi.1003670.s006.tif]

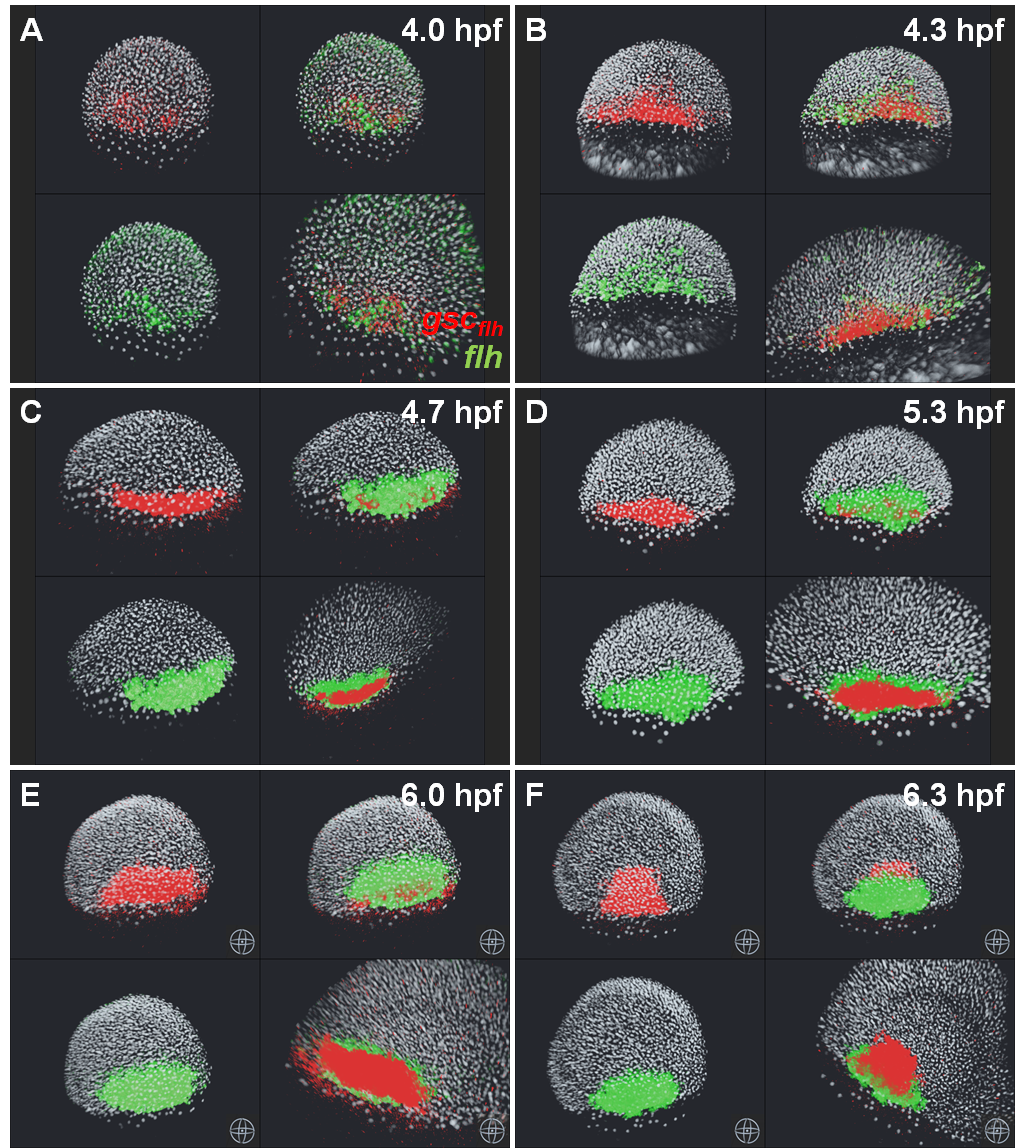

Supplement: Figure S7 — Raw data rendering of flh expression pattern. For every developmental stage, four panels are displayed: Top right: gsc (red) and flh (green) expressions viewed from the dorsal side, bottom right: gsc and flh expressions viewed from the ventral side, top left: gsc expression, bottom left: flh expression. The analyzed embryo's nuclei are shown in gray. (TIF) [file pcbi.1003670.s007.tif]

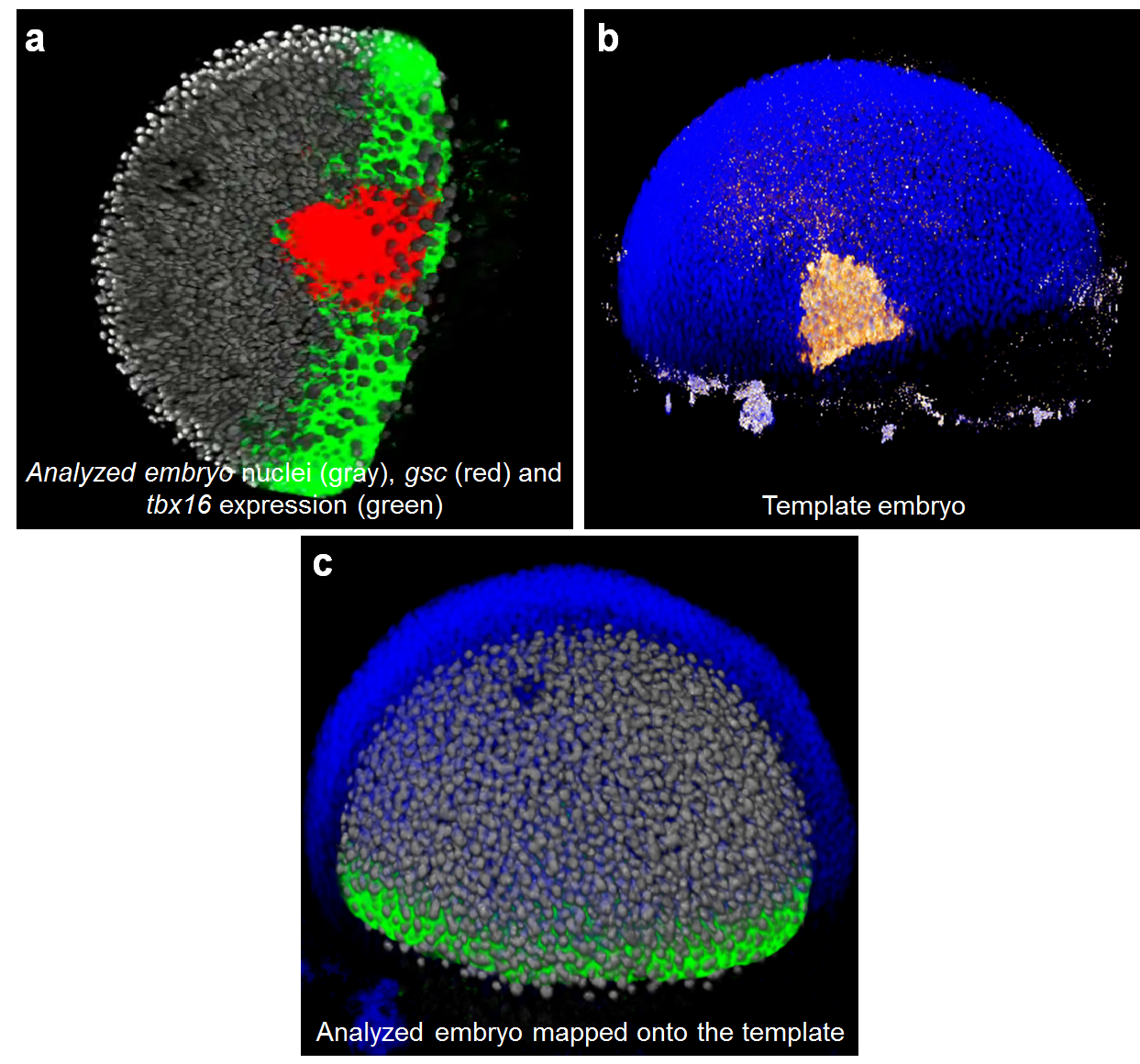

Supplement: Figure S8 — Raw data of one analyzed embryo mapped onto the template at 6.3 hpf. (a) Raw data rendering of one analyzed embryo showing the nuclei (gray), gsc expression (red) and tbx16 expression at 6.3 hpf. (b) Raw data rendering of the 3D template showing the nuclei (blue) and gsc expression (orange) at 6.3 hpf. (c) Analyzed embryo's nuclei (gray) and tbx16 expression (green) are showed superimposed on the template's nuclei (blue) after they were mapped with Match-IT. (TIF) [file pcbi.1003670.s008.tif]

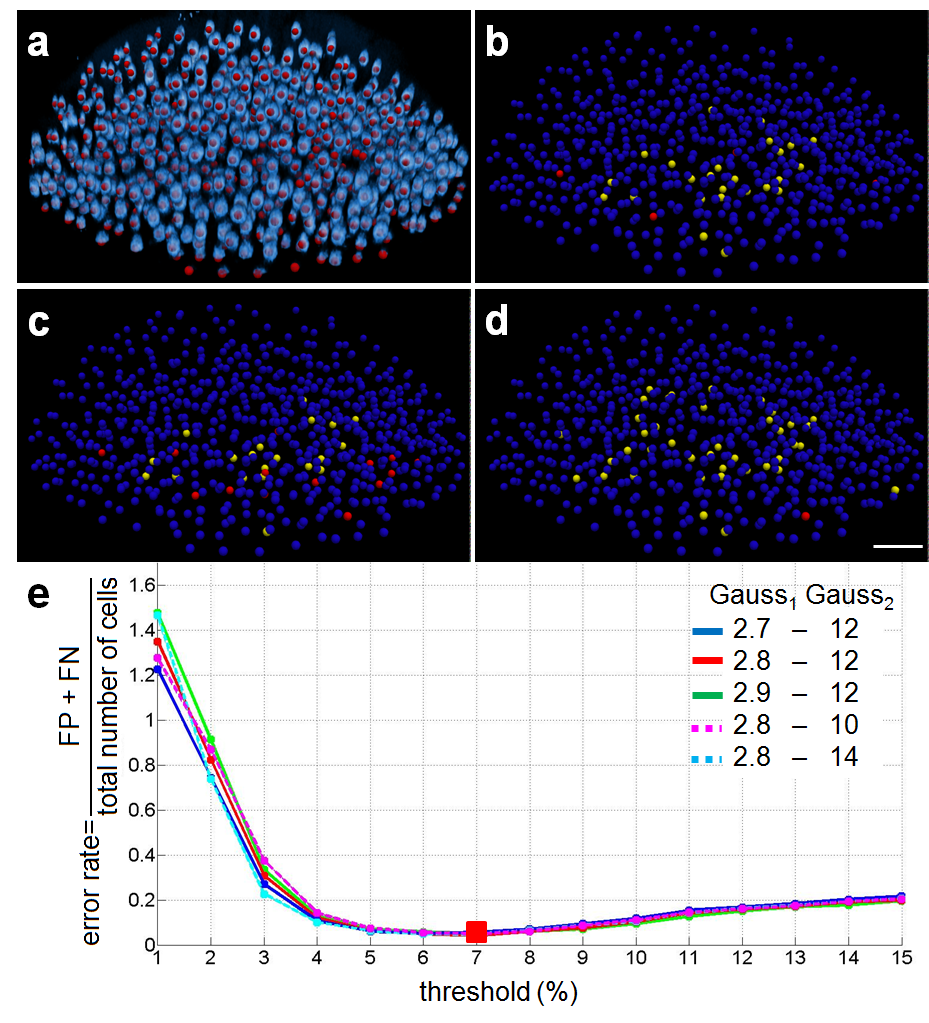

Supplement: Figure S9 — Evaluation of nuclear center detection. (a) Volume rendering of nuclear raw data (blue) together with the manually labeled “ground truth” (GT, red) for one analyzed embryo dataset. (b) Centers produced by our methodology (visually interactive choice of the optimal parameters by an expert) compared to the manually labeled GT. Out of the 689 cells in GT, there were 664 correct detections (blue), 4 false positives (FP, red), and 25 false negatives (FN, yellow), with a resulting error rate of 4.2% (). The chosen parameters were a 7% threshold, and 2.8 and 12 standard deviations for the two Gaussian kernels. A detection was considered correct when lying less than 4.2 (i.e. the approximate radius of the smallest nucleus in the dataset) from GT. (c) Centers produced by decreasing the detection threshold to 6%. As a consequence, the number of FN (yellow) is reduced to 20 at the cost of raising the number of FP (red) to 14 with a resulting error rate of 4.9%. (d) Centers yielded by increasing the detection threshold chosen by the expert to 8%. As a consequence, the number of FP (red) is reduced to 1 at the cost of raising the number of FN (yellow) to 40 with a resulting error rate of 5.9%. Scale bar 50 . (e) Variations of the error rate with respect to changes in the threshold and standard deviations. Blind results obtained by an expert following our methodology (red square) showed to be robust against possible variations around the selected parameters. (TIF) [file pcbi.1003670.s009.tif]

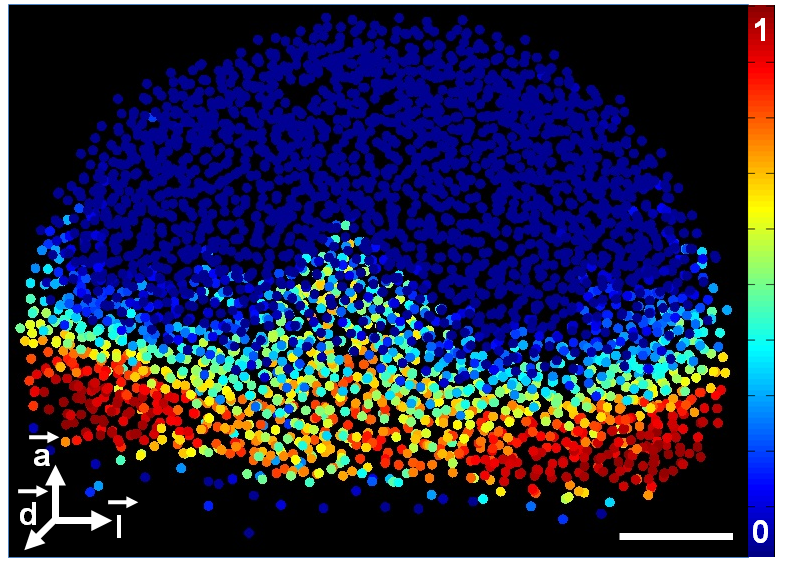

Supplement: Figure S10 — Gene expression quantification. 3D rendering of the relative gene expression levels measured at the cellular scale for tbx16 at 6.3 hpf. The gene expression levels range from 0 (dark blue) to 1 (red). Centered in each nucleus, a sphere with a radius equal to the average internuclear distance was used to measure the mean intensity values of the raw tbx16 expression. The mean background intensity, measured in the image regions outside the embryo, was subtracted from these values, which were also compensated by a depth penetration factor computed from the attenuation observed on the nucleus channel. Scale bar 50 . (TIF) [file pcbi.1003670.s010.tif]

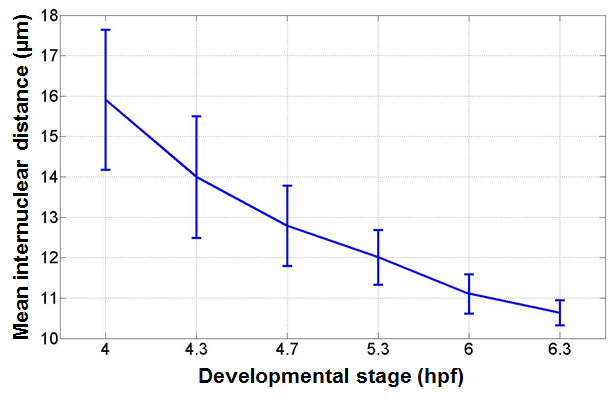

Supplement: Figure S11 — Evolution of the internuclear distance over time. The mean internuclear distance (in ) is calculated for different specimens at each stage. The observed decrease fits with an average of approximately divisions per cell between and hpf and an exponential decrease in the cell cycle length. This is in agreement with previous observations in literature and validates the accuracy of the center detection procedure. Standard deviation is interpreted as reflecting individual variations. (TIF) [file pcbi.1003670.s011.tif]

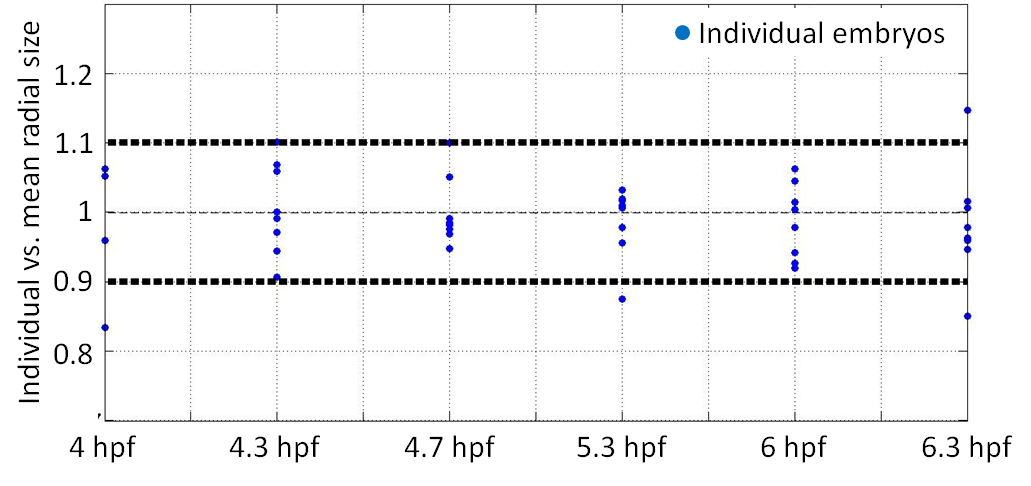

Supplement: Figure S12 — Quantification of the morphological variability among individual embryos. In 95% of the matched individuals, the radial size differs by less than 10% from the mean radius at each developmental stage. (TIF) [file pcbi.1003670.s012.tif]

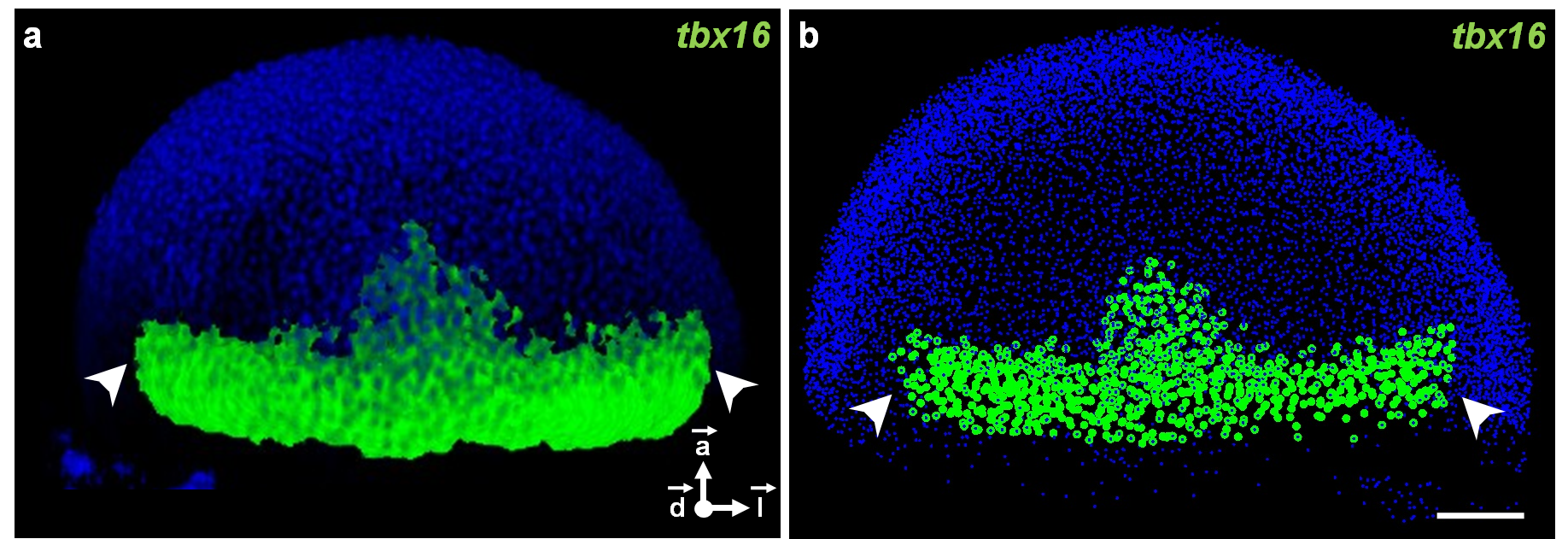

Supplement: Figure S13 — Positive cell selection in the template. (a) Raw tbx16 expression pattern (green) from the analyzed embryo mapped onto the template's raw nuclei (blue). (b) Template nuclei (blue) falling into the analyzed gene expression domain are considered positive (green). White arrowheads indicate the limits of the imaged analyzed embryo. Scale bar 100 . (TIF) [file pcbi.1003670.s013.tif]

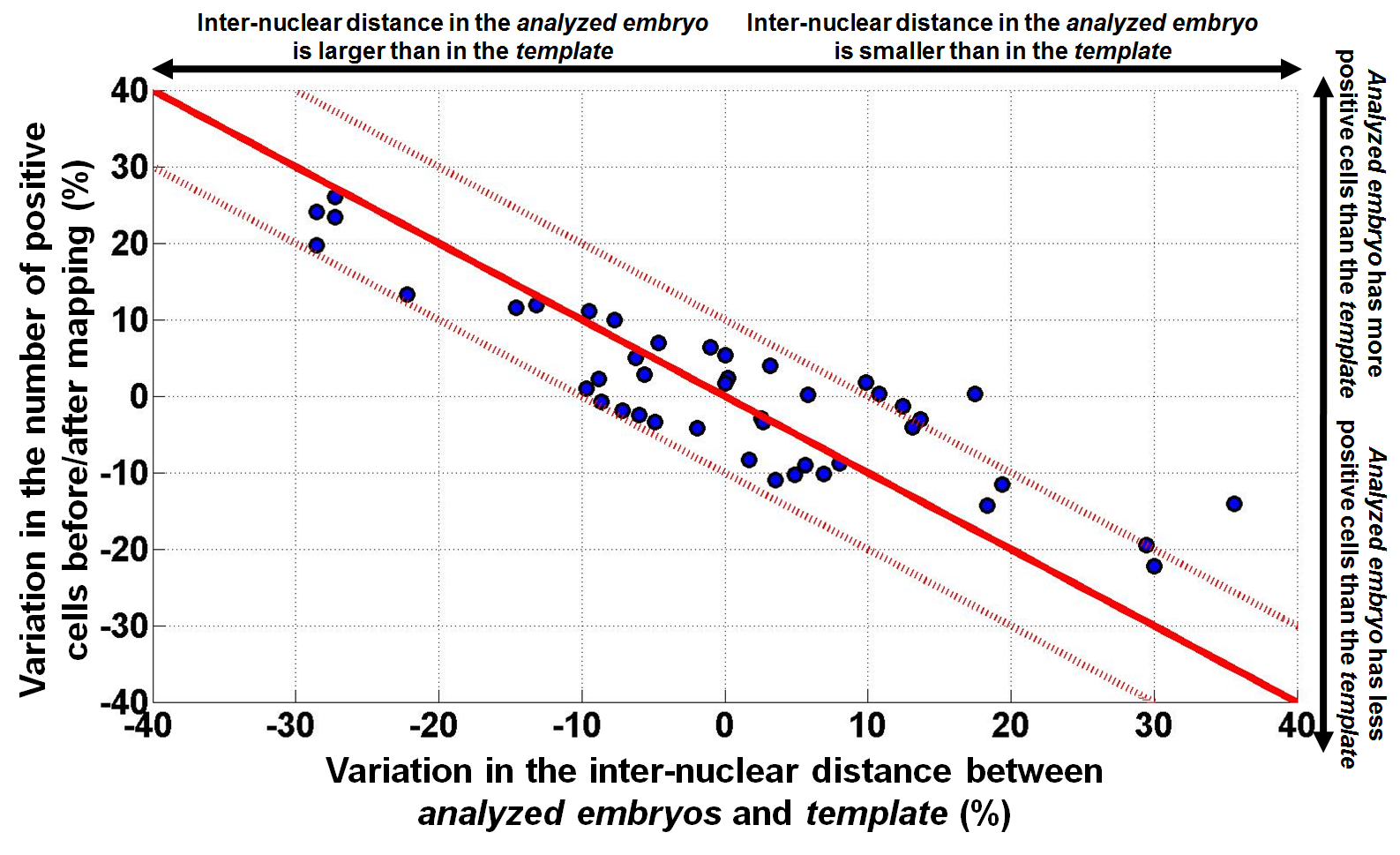

Supplement: Figure S14 — Variation in the number of positive cells between analyzed embryos and template as a function of their relative internuclear distance. More than of the analyzed embryos fall within a deviation from the identity function, yielding a statistical p-value of . The two specimens deviating from this norm in the plot come from a very early developmental stage, hpf, when staging is more difficult due to the lack of morphological traits. (TIF) [file pcbi.1003670.s014.tif]

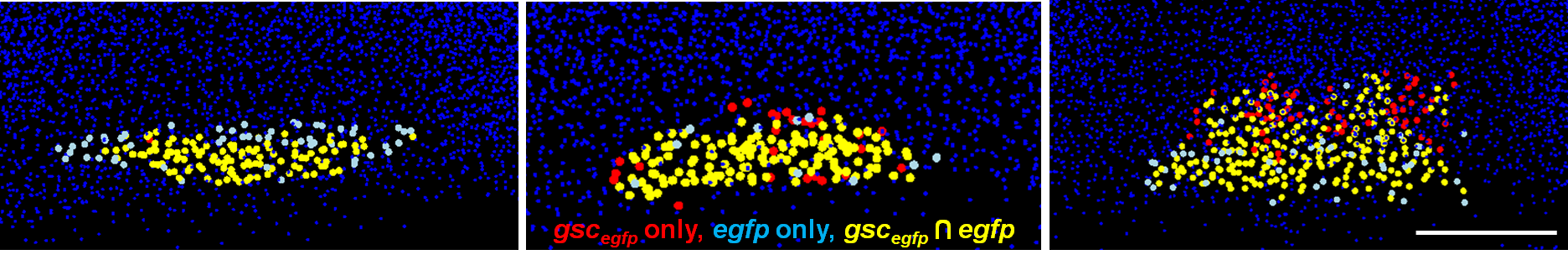

Supplement: Figure S15 — Evolution of the egfp - gsc pair through time after being mapped onto the template. Atlas-IT interface displaying the template nuclei (dark blue) and the coexpression (yellow) between the gsc and egfp expressions. Scale bar 100 . (TIF) [file pcbi.1003670.s015.tif]

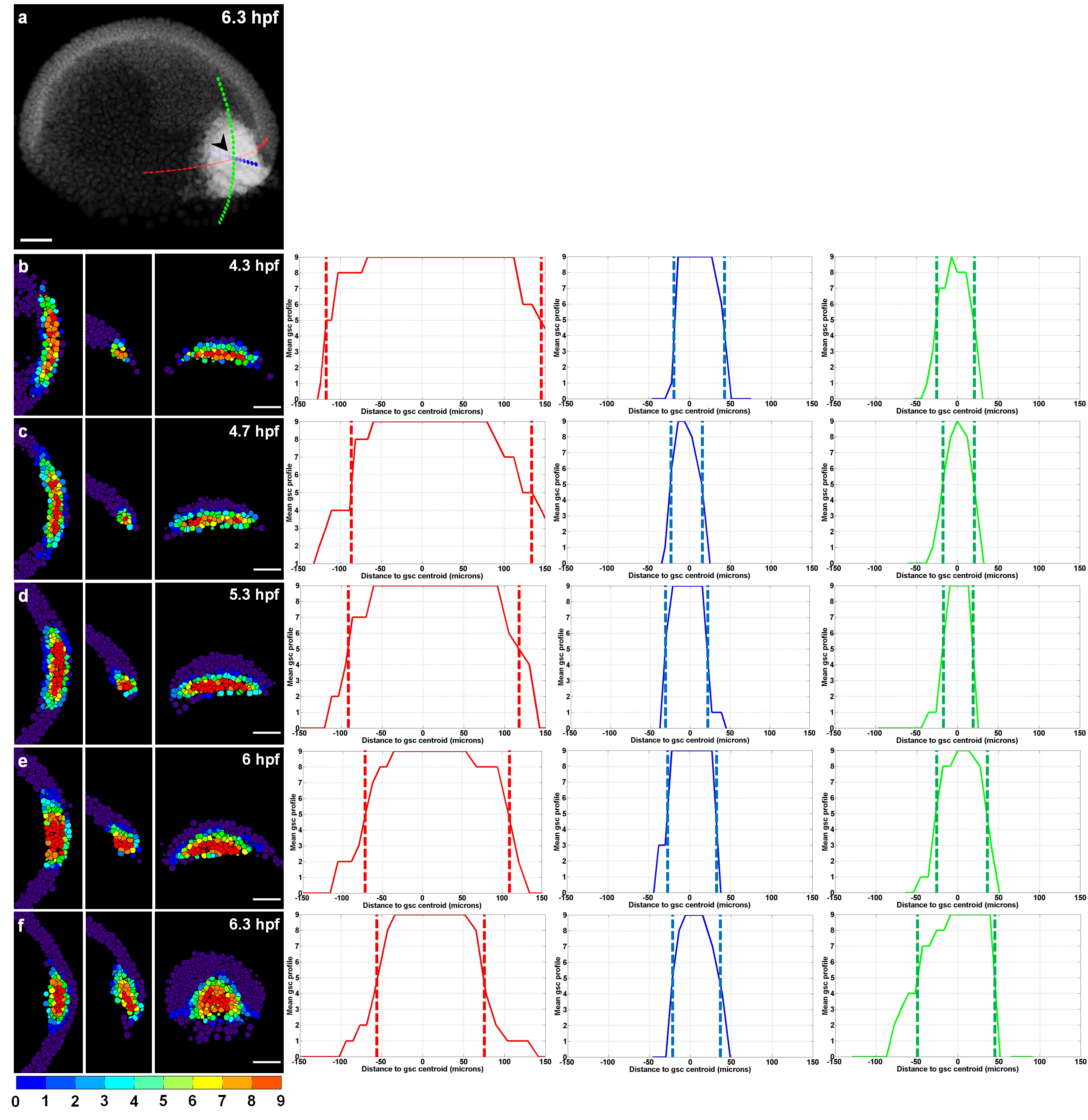

Supplement: Figure S16 — Variability of the gsc gene expression pattern. (a) Volume rendering of the aggregated gsc expressions, gsc , together with the three cutting lines along which variability is measured: lateral line (red), radial line (blue) and sagittal line (green). The black arrowhead indicates the gsc centroid. (b–f) Left panel: From left to right: equatorial, sagittal and dorsal orthoslices passing through the gsc expression domain at the level of its centroid. The color code indicates the number of gsc expression repetitions in the template cells based on the analysis of the available specimens. Right panel: Profile showing how many embryos (out of the mapped individuals) expressed gsc along the three cutting lines centered at the gsc centroid as displayed in (a). Expression variability appears as additional rows of cells around a core domain (where cells are positive for all the observed specimens). The dotted lines indicate the borders of the gsc pattern. (TIF) [file pcbi.1003670.s016.tif]

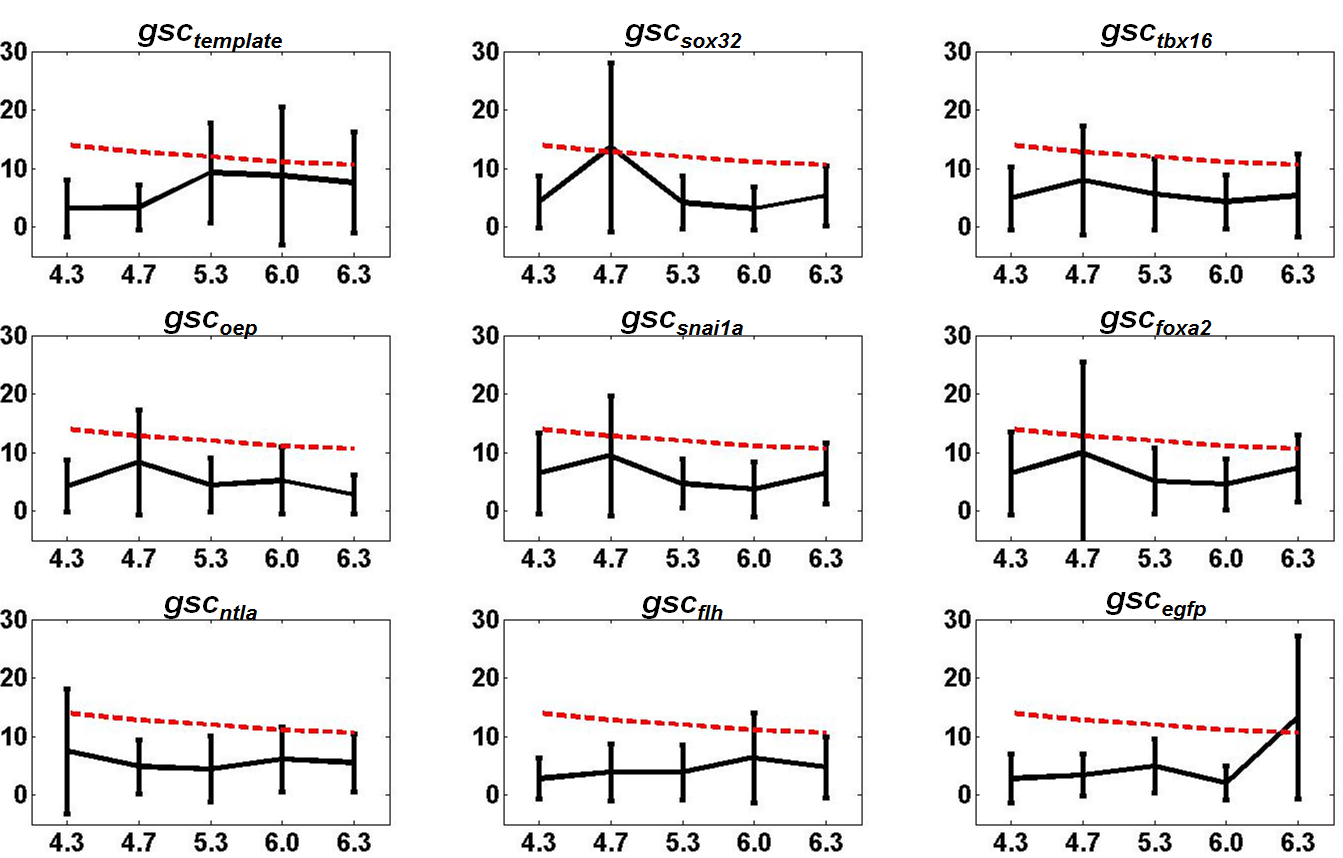

Supplement: Figure S17 — Quantification of pattern differences: evaluation of gsc expression variance after mapping. Each of the individual gsc segmented domains mapped onto the atlas were compared to the mean gsc following a leave-one-out strategy at every developmental stage between 4.3 and 6.3 hpf. The error bars represent the mean and the standard deviation of the distance, in , between the individuals' gsc borders and their corresponding gsc . The average internuclear distance (dotted red line) ranges from 14 at 4.3 hpf down to 10 at 6.3 hpf (see Fig. S11a). As discussed for Drosophila embryos, individual variations in gene expression patterns, in terms of positive cell numbers or domain topology, could arise from gene expression regulation itself, and from geometric variations such as embryo size and cell proliferation rate variability. In the zebrafish early embryo, overall size, internuclear distance, and cell proliferation rate are dependent parameters (Fig. S11 and Fig. S26b). Internuclear distance, expected to decrease through cell divisions until the end of gastrulation (10 hpf), was indeed variable among specimens, but converged toward similar values (Fig. S11a). There was however no clear correlation between embryo size and internuclear distance, possibly indicating variability in the proliferation rate and/or developmental speed of our batches of embryos. Because of the difficulty to separate the different components of variability, our atlasing strategy did not attempt to minimize it but introduced the calculation of mean expression domains (Fig. S16). (TIF) [file pcbi.1003670.s017.tif]

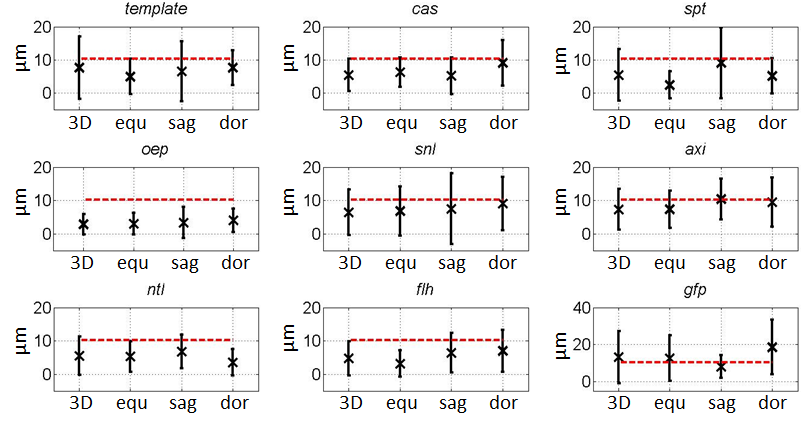

Supplement: Figure S18 — Quantification of pattern differences on the main embryo planes. Mean and standard deviation of the distance, in , between the individuals' gsc borders and their corresponding gsc at 6.3 hpf. Distances were obtained using the expression contours in 3D (as performed in Fig. 17) and restricting them to the three main embryo planes: equatorial, sagittal and dorsal (see Fig. 4b and Fig. S16a). (TIF) [file pcbi.1003670.s018.tif]

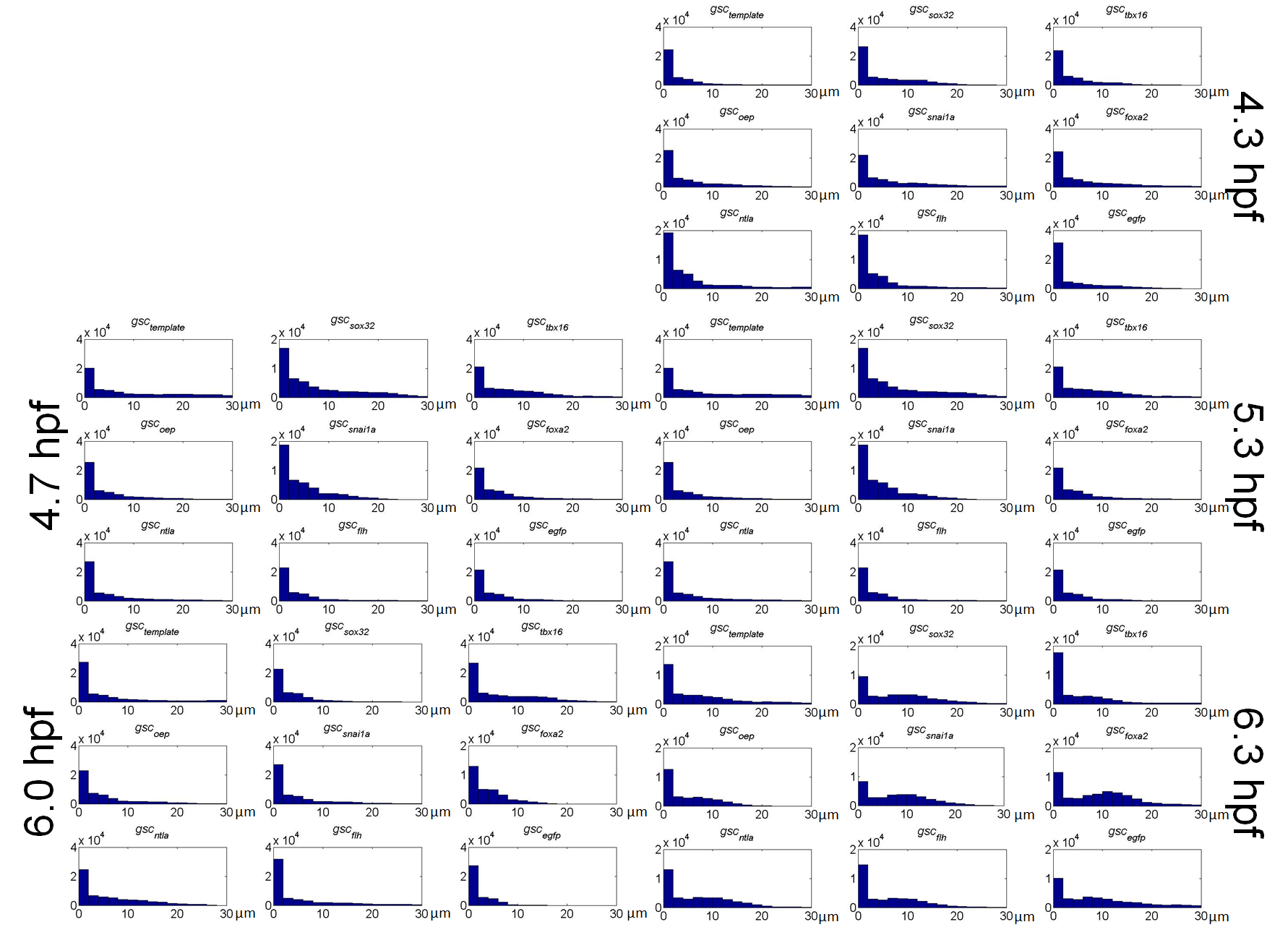

Supplement: Figure S19 — Quantification of pattern differences along the domain borders. For each developmental stage: histogram of the Hausdorff distances from the all the points placed at the complete outer border of each individual's gsc expression to the closest boundary point of the gsc pattern. The vast majority of these boundaries is within two cell rows from gsc (cell diameter is 12 ). This constitutes an upper bound for the registration quality, as it reflects the variability in the mapping procedure plus the intrinsic interembryo variability. (TIF) [file pcbi.1003670.s019.tif]

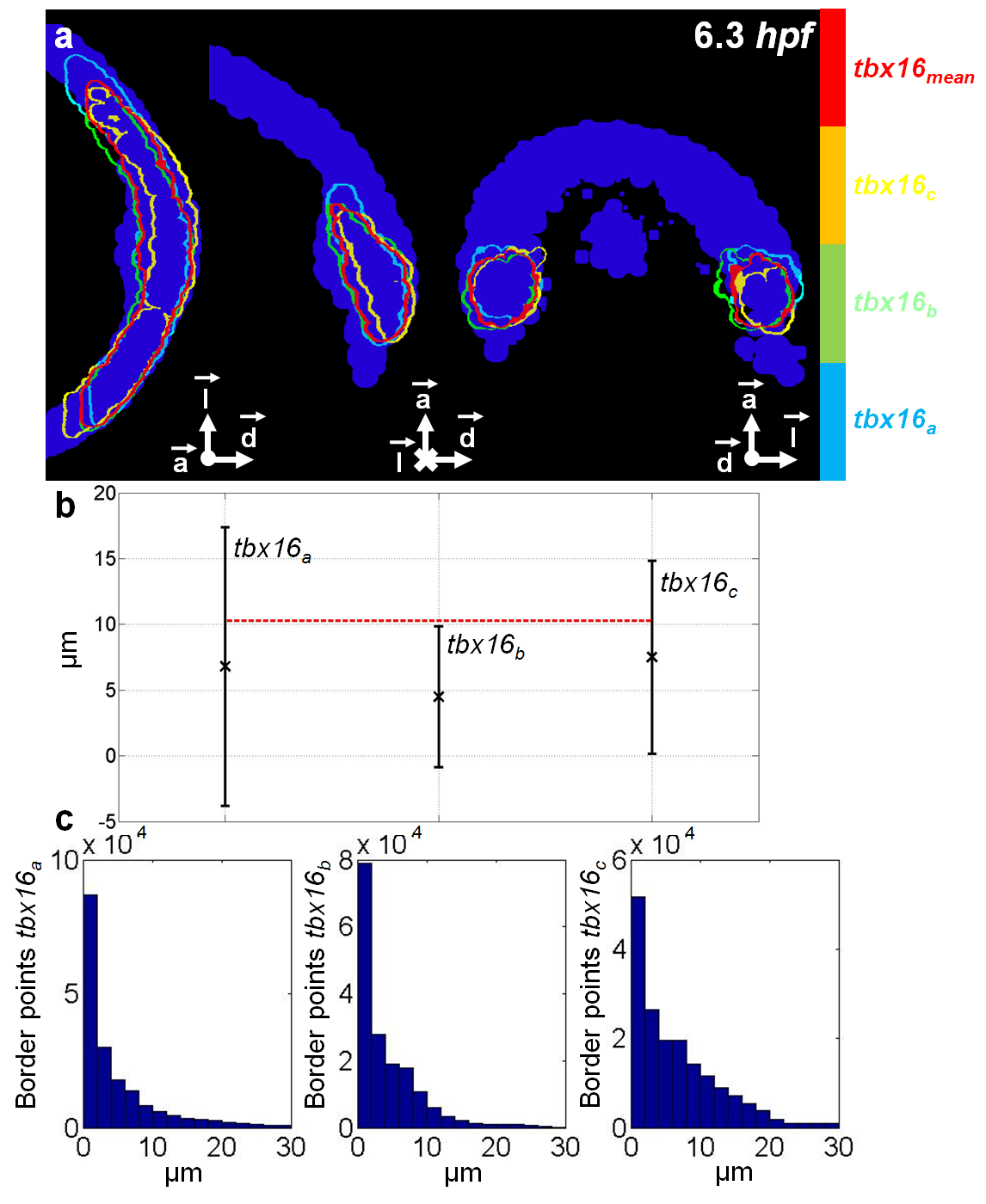

Supplement: Figure S20 — Quantification of pattern differences: evaluation of tbx16 expression variance after mapping. (a) Each of the individual tbx16 segmented domains mapped onto the atlas were compared to the mean tbx16 following a leave-one-out strategy at 6.3 hpf. (b) Mean and standard deviation of the distance, in , between the individuals' tbx16 borders and the tbx16 pattern. The average internuclear distance (dotted red line) is 10.3 at 6.3 hpf (see Fig. S11a). (c) Histograms of the distance, in , between the points located at individual tbx16 borders to the closest point at the tbx16 border. (TIF) [file pcbi.1003670.s020.tif]

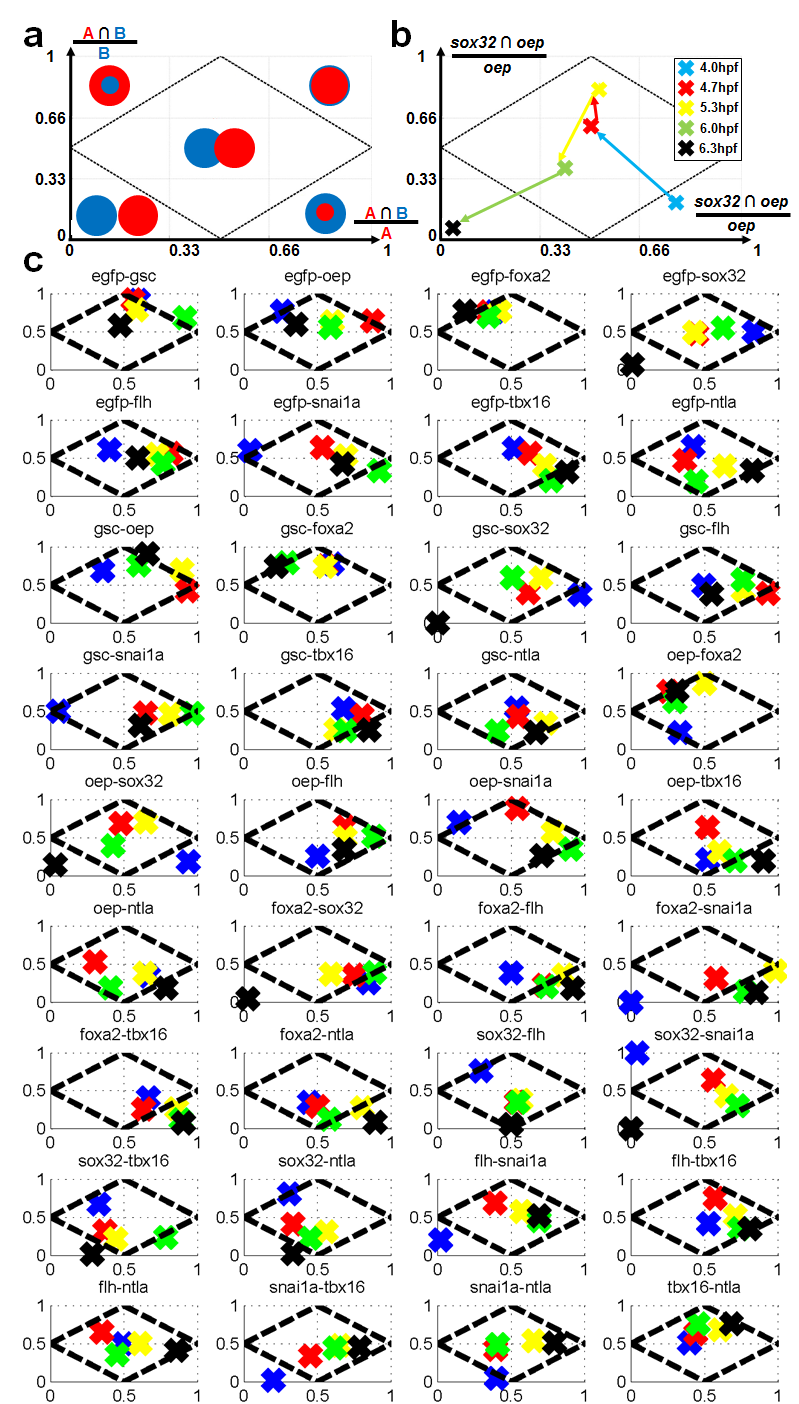

Supplement: Figure S21 — A synthetic view of gene coexpression pairs and their evolution through time. (a) Gene coexpression pairs fell into possible categories defined by gene pattern similarity: and expression domains exclude each other (bottom left), is included in (bottom right), is included in (top left), is identical to (top right), and domains partially overlap (center). (b) This chart allows a visualization of the segregation of oep-sox32 coexpression through time. (c) Gene pattern relationships and their evolution in time for the possible pairs. Coherence with a priori knowledge has been checked and demonstrates the power of the atlas construction strategy and further analysis tools. (TIF) [file pcbi.1003670.s021.tif]

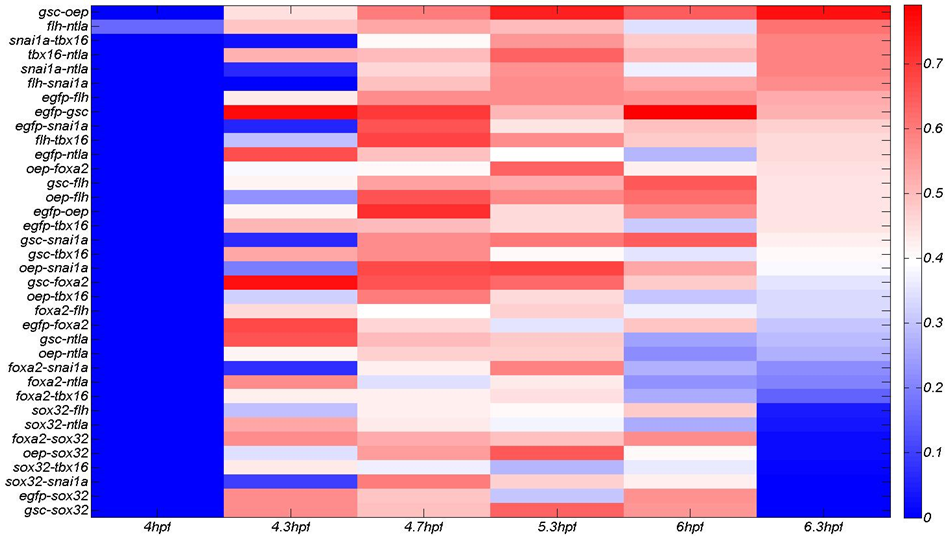

Supplement: Figure S22 — Evolution of similarity coefficient for all possible gene pairs. Dice's similarity coefficient: was calculated for the gene pairs, which were then arranged in descending order according to at hpf. (TIF) [file pcbi.1003670.s022.tif]

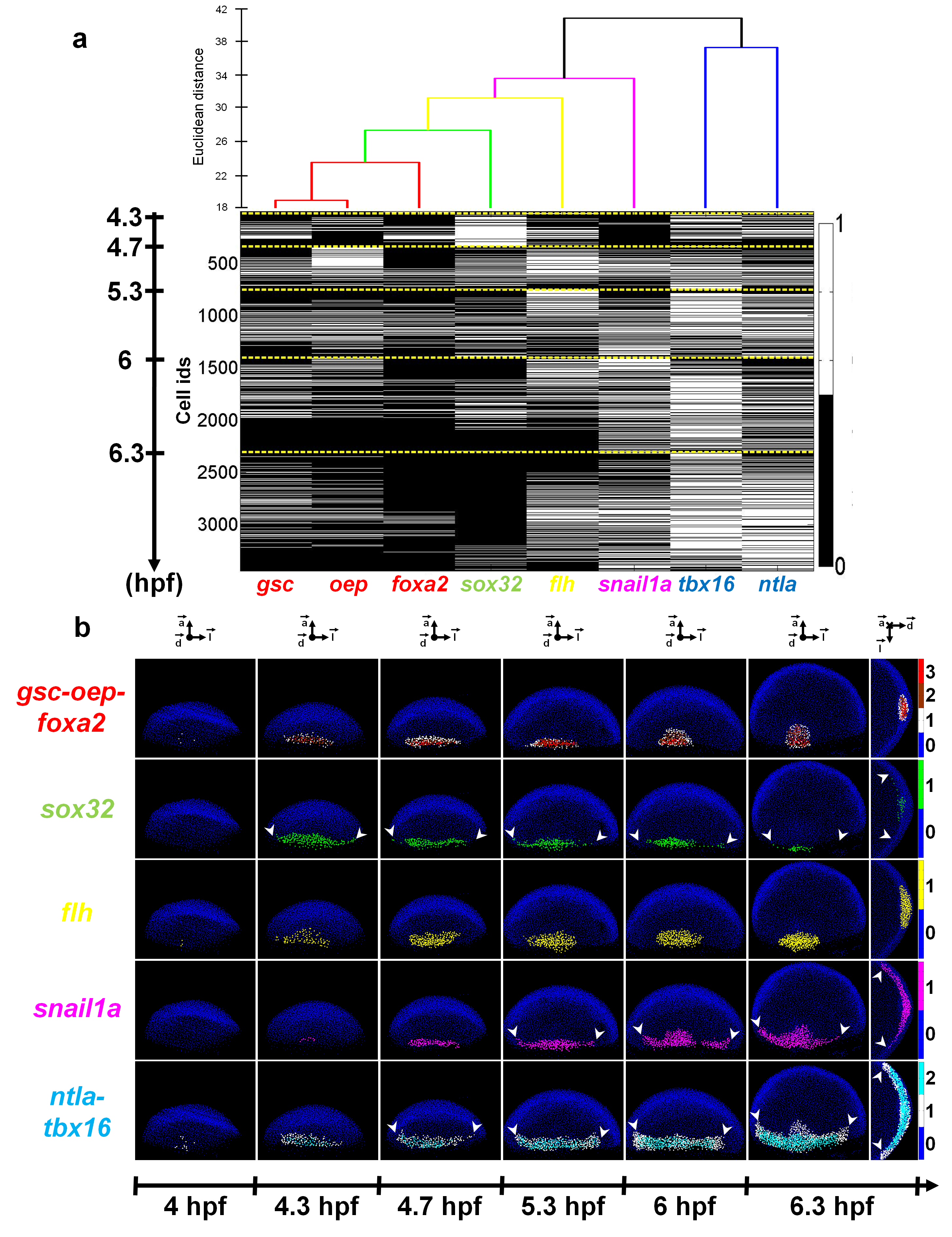

Supplement: Figure S23 — Gene synexpression groups defined by their spatiotemporal clustering patterns. (a) A hierarchical clustering of genes according to the similarity of their spatiotemporal regions of expression defined 5 different groups with characteristic spatiotemporal behaviors. For each group, a color code (column to the right of the panel) was displayed to indicate whether cells expressed 0, 1, 2 or 3 genes. The 8 analyzed genes fell into the following synexpression groups: gsc-oep-foxa2, sox32, flh, snail1a, ntla-tbx16. (b) Visualization of the synexpression groups identified in (a). Arrowheads indicate the limits of the imaged volume in the analyzed embryos. (TIF) [file pcbi.1003670.s023.tif]

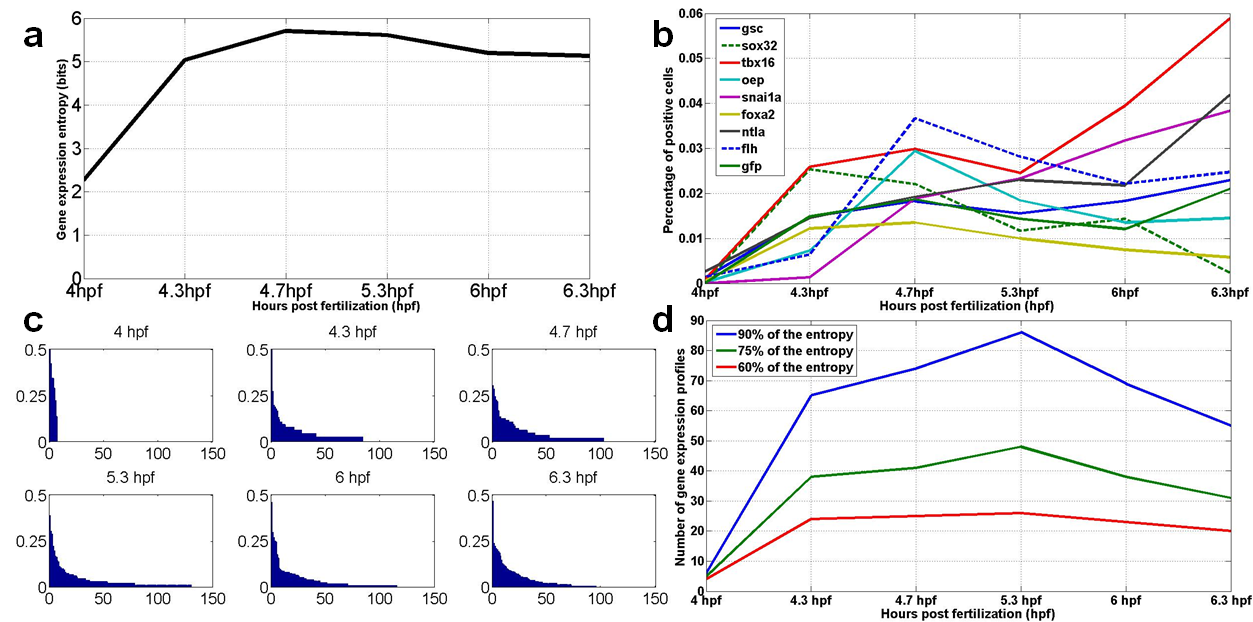

Supplement: Figure S24 — Gene expression entropy. (a) Gene expression entropy as a function of time: the Shannon entropy provides a measurement of the complexity of a cell's gene expression profile. (b) Percentage of positive cells for each gene expression as a function of time. A gene expression (inhibited until a certain time step) that would suddenly start expressing would make the entropy increase by 1 bit at most. (c) Quantity of information (in bits) contributed by each gene expression profile at each time step. Expression profiles are sorted by decreasing contribution to the information. Only the first 150 profiles are plotted. We can observe that many of the possible gene expression profiles are actually never used, and most of the information is conveyed by a small number (around 100) of representative combinations. (d) Number of gene expression profiles required to convey 60% (red line), 75% (green line) and 90% (blue line) of the total entropy at each time step. The ascending slopes from 4.0 to 5.3 hpf are compatible with the time trend toward more equidistribution visible in (c). (TIF) [file pcbi.1003670.s024.tif]

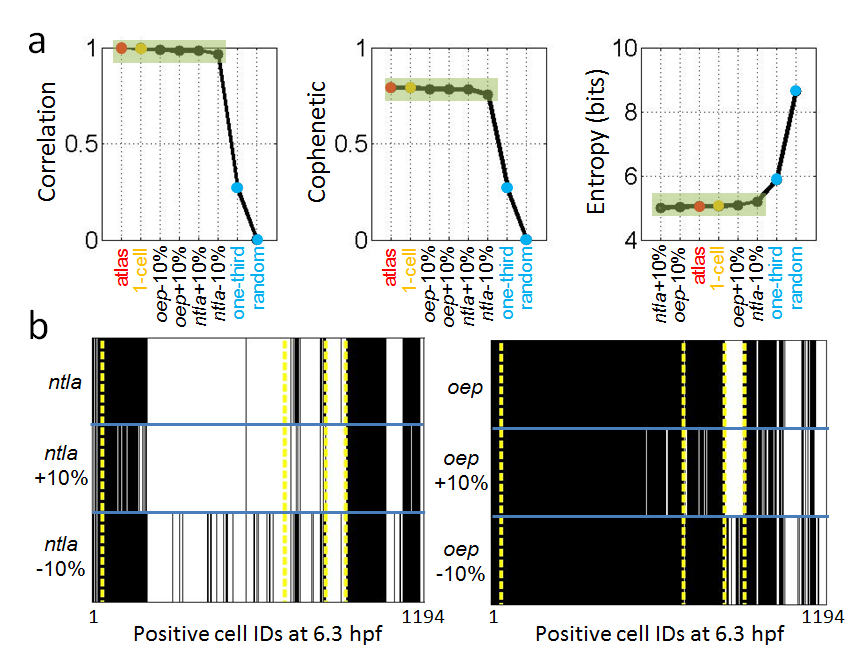

Supplement: Figure S25 — Robustness of entropy and clustering with respect to gene segmentation thresholds. (a) From left to right: correlation between the distance matrix, cophenetic coefficients and entropy of the original and modified atlases. Threshold modifications in the expressions of oep and ntla (labeled ‘oep-10%’, ‘oep+10%’, ‘ntla-10%’, ‘ntla+10%’) showed metrics similar to the original atlas (labeled ‘atlas’) or a minimally modified atlas (labeled ‘one-cell’), and are all grouped around one value (green rectangle). They are clearly distinct from other, severe modifications in the atlas, such as substituting one third of its values (labeled ‘one-third’) or using a randomly generated atlas (labeled ‘random’). (b) Original cell values for ntla (left) and oep (right) in the atlas at 6.3 hpf (as seen in Fig. 5b ) are compared to the values obtained after modifying by % the thresholds originally chosen by the biologist expert. (TIF) [file pcbi.1003670.s025.tif]

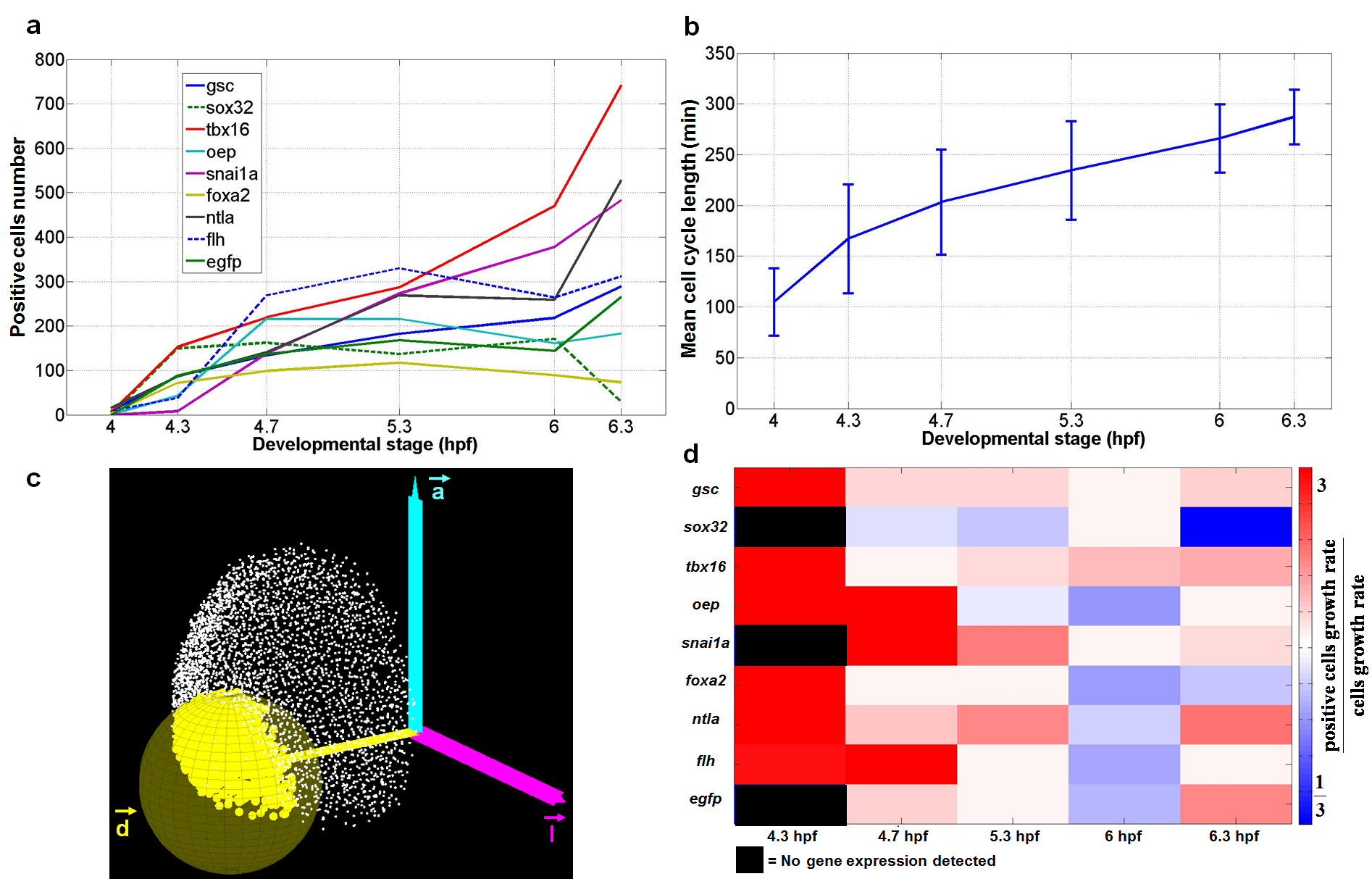

Supplement: Figure S26 — Correlation between cell proliferation and the expansion of gene expression domains. (a) Evolution of the number of positive cells for each of the considered gene products. (b) Temporal evolution of the number of cells in the region of interest (ROI) centered on the dorsal side of each analyzed embryo as shown in (c). The cell proliferation rate extracted from this experiment matched previous observations from the literature. (c) Dorsal region of interest (ROI) used to measure the cell proliferation rate in each analyzed embryo. (d) Ratio between the increase rate of positive cells for a given gene and the estimated overall cell proliferation rate. This ratio indicates whether the dynamics of gene expression patterns can be explained by sustained expression in proliferating cells or requires upregulation (such as egfp by 6.3 hpf) or downregulation (such as sox32 by 6.3 hpf). (TIF) [file pcbi.1003670.s026.tif]

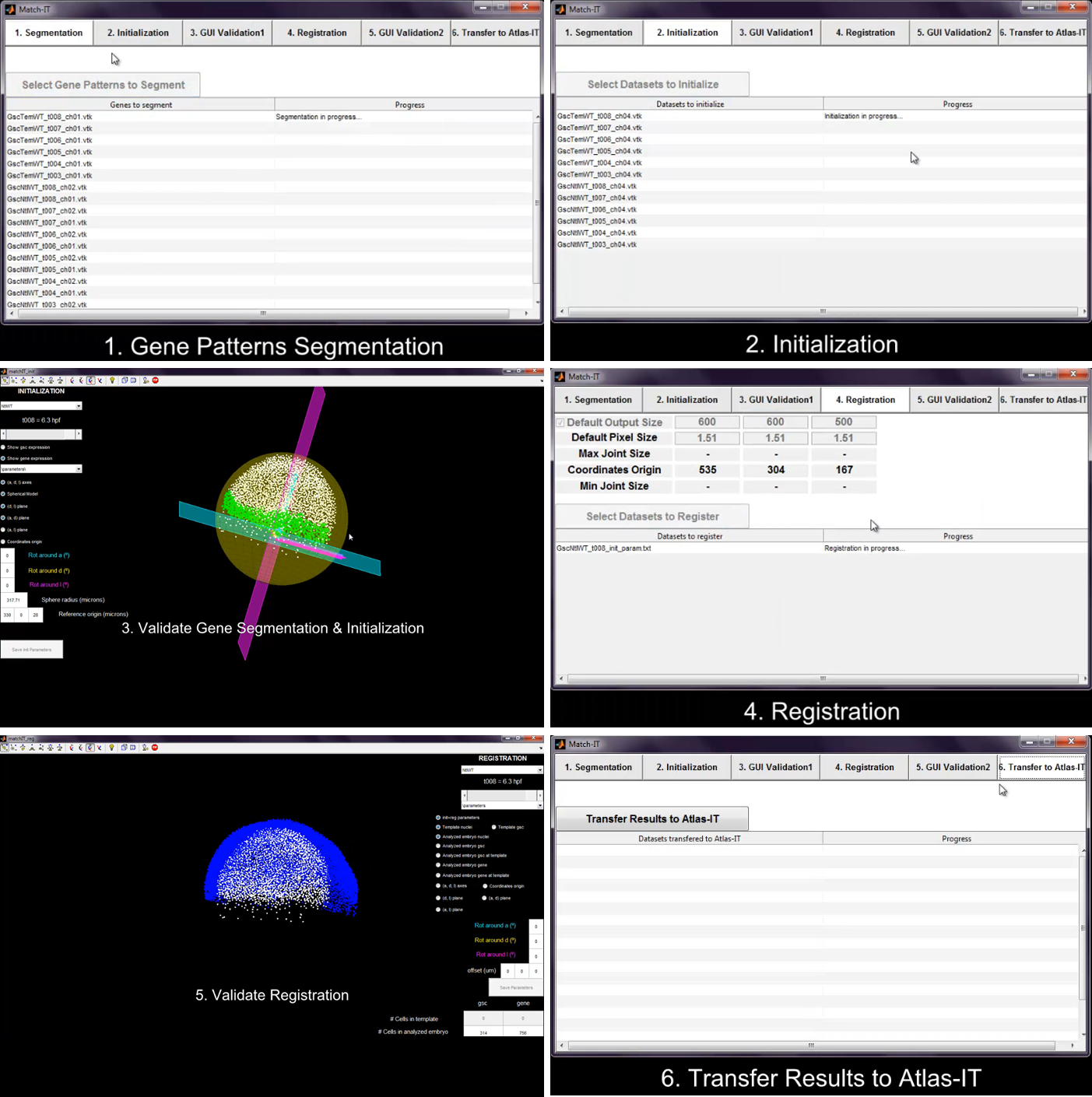

Supplement: Software S1 — Match-IT: A software package to map gene expression data at the cellular-scale onto an atlas model. (a) Main window. b) Validation Graphical User Interface (GUI). A tutorial on using Match-IT can be found as an annex to this document. The Match-IT software package together with its tutorial and representative datasets can be downloaded from the Bioemergences website http://bioemergences.iscpif.fr/documents/MatchIT.zip. (TIF) [file pcbi.1003670.s031.tif]

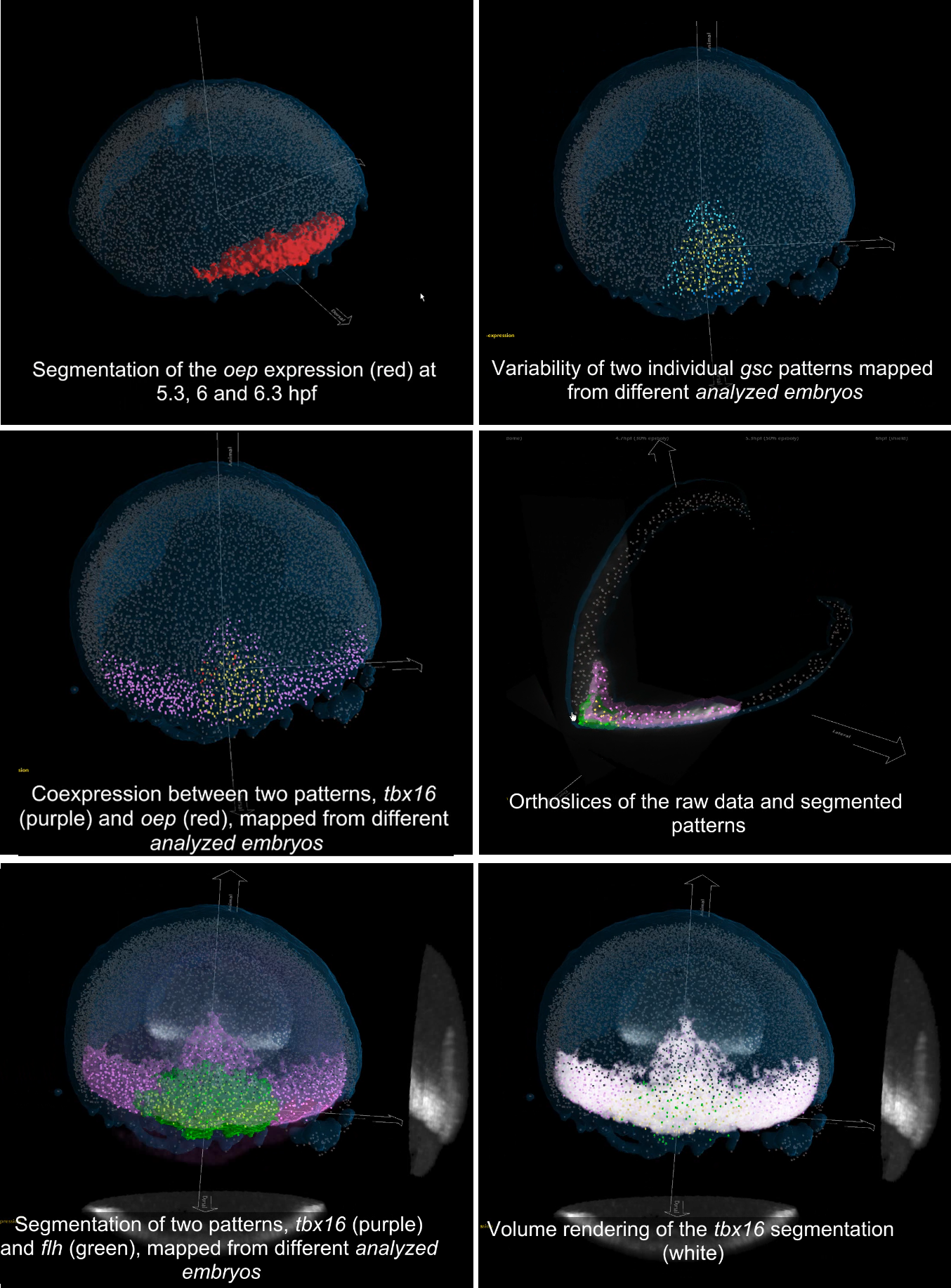

Supplement: Software S2 — Atlas-IT: A software package to visualize and analyze an atlas of gene expression at the cellular scale. A tutorial on using Atlas-IT can be found as an annex to this document. The Atlas-IT software package together with its tutorial and representative datasets can be downloaded from the Bioemergences website http://bioemergences.iscpif.fr/documents/AtlasIT.zip. (TIF) [file pcbi.1003670.s032.tif]

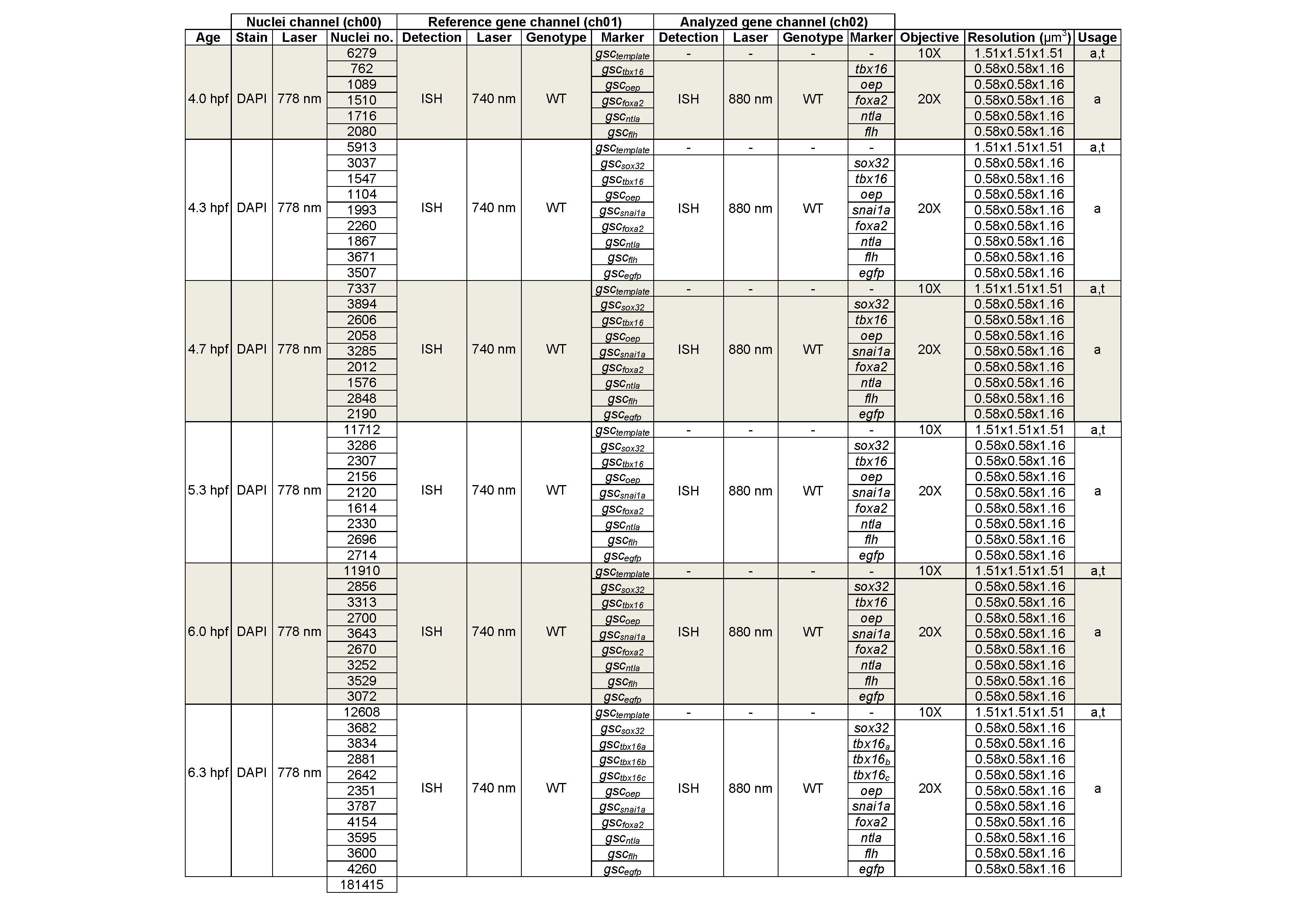

Supplement: Table S1 — Acquisition details of the early zebrafish microscopy datasets included in the atlas. “Usage” column: a = included in the atlas, t = template. “Genotype” column: WT = wild type. “Detection” column: ISH = in situ hybridization. (TIF) [file pcbi.1003670.s033.tif]
