# Supplementary material for: A Digital Framework to Build, Visualize and Analyze a Gene Expression Atlas with Cellular Resolution in Zebrafish Early Embryogenesis
Source: PLoS Comput Biol. 2014 Jun 19;10(6):e1003670. doi: 10.1371/journal.pcbi.1003670 (PMC4063669; doi:10.1371/journal.pcbi.1003670)
Supplement: User Guide S1 — Match-IT User Guide: A step-by-step protocol. This user guide is available as Supplementary Material to this paper and at http://bioemergences.iscpif.fr/documents/MatchIT.zip. (PDF) [file pcbi.1003670.s034.pdf]

A digital framework to build, visualize and analyze a gene expression atlas with cellular resolution in zebrafish early embryogenesis

Carlos Castro-González[1,2,3], Miguel A. Luengo-Oroz[1,2], Louise Duloquin[4,5,6], Thierry Savy[4,5,6], Barbara Rizzi[4,5,6], Sophie Desnoulez[4,6], René Doursat[5,6,7], Yannick L. Kergosien[4,6,8], María J. Ledesma-Carbayo[1,2], Paul Bourgine[4,5,6], Nadine Peyriéras[4,5,6,*,†], Andrés Santos[1,2,*,†]

1 User Guide S1 - Match-IT step-by-step protocol

What is Match-IT?

Match-IT is a software package to build cellular-level atlases of gene expression in early embryogenesis. It gathers gene expression acquired by in-situ hybridization from multiple, partial analyzed embryos into one single, complete template. It also performs segmentation of the analyzed gene expression patterns and offers the possibility to supervise and correct the final results through a graphical user interface. Match-IT is delivered as part of the following publication: C. Castro-Gonzlez, M.A. Luengo-Oroz, L. Duloquin, T. Savy, B. Rizzi, S. Desnoulez, R. Doursat, Y. Kergosien, M.J. Ledesma-Carbayo, P. Bourgine, N. Peyriras and A. Santos. "A digital framework to build, visualize and analyze gene expression atlases with cellular resolution in zebrafish early embryogenesis".

A public release of Match-IT together with the present tutorial, a readme.txt file and representative datasets are available as Supplementary Material to this paper:

http://bioemergences.iscpif.fr/documents/MatchIT.zip

System requirements

- Windows 64 bits (Xp or higher)
- Having either Matlab R2010b (64 bits) or Matlab Compiler Runtime v714 (64 bits) installed in your computer. In case you have neither of them installed, the Matlab Compiler Runtime installer ("MCRInstaller.exe") is provided within the Match-IT package
- 8GB RAM (minimum)
- The disk space needed to load the provided example data and to run Match-IT on it is approximately 2GB

Installing Match-IT

To run the program, simply unzip the software package and double-click on "Match-IT.exe". Representative confocal datasets (found within the "data" folder of the Match-IT package) must be uncompressed before running the program.

Using Match-IT

In order to map one analyzed embryo onto the common template, follow the 6 processing steps described below.

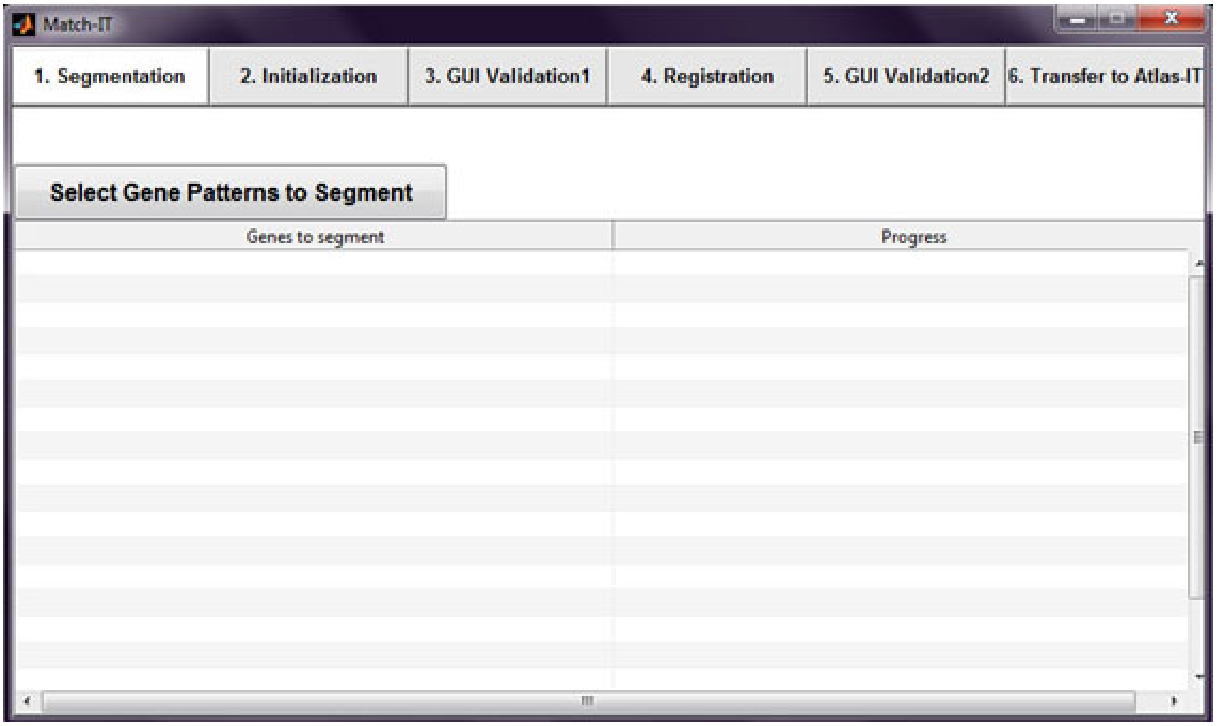

Gene Segmentation Tab:

This tab performs the segmentation of the specified raw gene patterns and identifies the selection of cells expressing those genes in the analyzed embryos.

Press the "Select Gene Patterns to Segment" button. This will open a file selection dialog that will let you choose, within the local "data" folder, the files that hold the raw gene expression patterns:

- gsc_G_YY_tTTT_ch01.vtk = *gsc* channel
- gsc_G_YY_tTTT_ch02.vtk = gene channel

where:

- "gsc" stands for *goosecoid*, the gene pattern used for reference in our atlas which was tagged for all our acquired datasets in ch01
- "G" is the variable-length name designating the specific gene expression, G, which accompanies gsc in ch02 (e.g. "ntla"). Files whose "G" name is "tem" denote the template specimen.
- "YY" is a 2-letter code which can take any free value (e.g. "WT" in our sample datasets)
- "TTT" is a 3-digit number specifying the time stage of the dataset (e.g. "008" designates developmental stage called late shield)

NOTE: To successfully perform gene pattern segmentation, Match-IT requires the lower value that best define each raw gene expression. This value must be specified in the "config.txt" file in the root folder. After performing the gene pattern segmentation ($\tilde{5}$ min), Match-IT identifies the selection of cells in the analyzed embryo that express that gene pattern. To this end, it requires the specification of the analyzed embryo nuclei coordinates held in a text file in the "data" folder:

- gsc_G_YY_tTTT_ch00.emb = nuclei coordinates

The outputs of this first processing step are:

- gsc_G_YY_tTTT_ch01.csv = selection of cells expressing gsc
- gsc_G_YY_tTTT_ch02.csv = selection of cells expressing gene G
- gsc_G_YY_tTTT_ch03.vtk = nuclei shape segmentation
- gsc_G_YY_tTTT_ch04.vtk = gsc segmentation
- gsc_G_YY_tTTT_ch05.vtk = gene G segmentation

which can be found in the local "data" folder. And:

- gsc_G_YY_tTTT_distance.txt = mean internuclei distance

which can be found in the local "parameters" folder.

Initialization Tab:

This tab performs the initial coarse alignment between the analyzed embryos and the template by extracting common orientation planes.

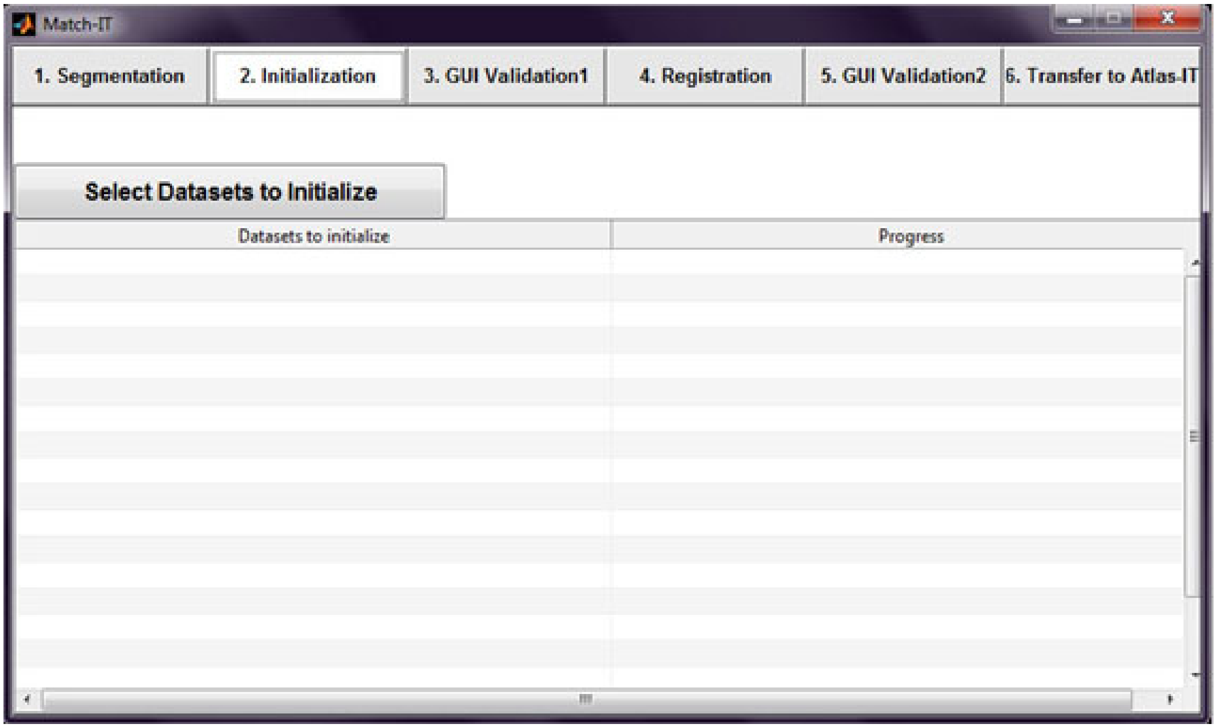

Initialization of one analyzed embryo at one given developmental stage requires the previous segmentation, in step 1, of the gsc pattern of both that particular analyzed embryo and its corresponding template.

Consequently, pressing the "Select Datasets to Initialize" button will only let you choose among those datasets having already a gsc segmentation file:

- gsc_G_YY_tTTT_ch04.vtk = gsc segmentation

in the local ”data” folder.

Apart from the gsc segmentation image, this initialization step also requires to read the corresponding analyzed embryo nuclei coordinates from the local ”data” folder:

- gsc_G_YY_tTTT_ch00.emb = nuclei coordinates

The output of this second processing step is a text file in the local ”parameters” folder which holds the extracted initialization information:

- gsc_G_YY_tTTT_init_param.txt = initialization parameters

Validate Initialization Tab:

This tab starts Match-IT Graphical User Interface (GUI) to supervise the orientation planes extracted in step 2 and correct them if necessary.

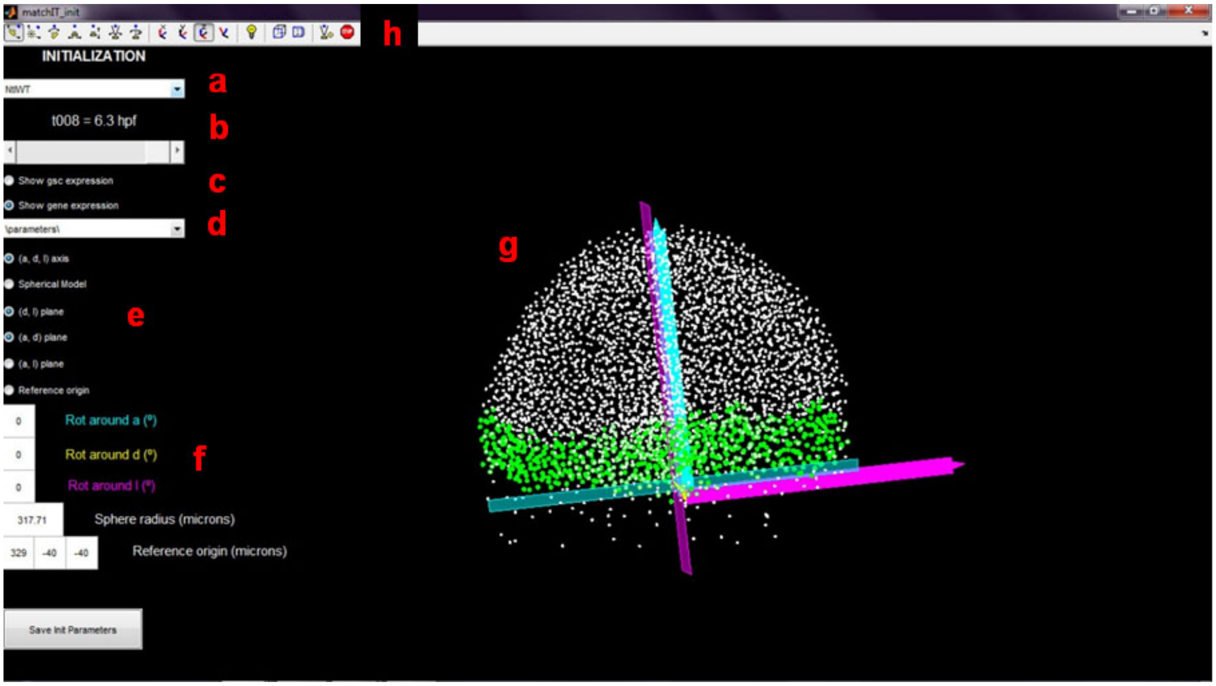

The components of the GUI are:

(a) Dataset selector: It pops up a menu to choose which analyzed embryo to visualize. It requires reading the corresponding analyzed embryo nuclei coordinates from the local ”data” folder:

gsc_G_YY_tTTT_ch00.emb = nuclei coordinates

(b) Time selector: It allows sliding through different developmental stages of the same dataset (i.e. of the same gene expression G)

(c) Gene selectors: These two radio buttons permit to highlight the selection of cells expressing either gsc or gene G for a particular analyzed embryo. They consequently require analyzed embryo expressions

to have undergone processing step 1 since they need to read cell selections stored in the local "data" folder:

gsc_G_YY_tTTT_ch01.csv = selection of cells expressing gsc

gsc_G_YY_tTTT_ch02.csv = selection of cells expressing G

(d) Parameter selector: It pops up a dialog to choose the folder where to find the initialization parameters text file (by default this folder is set to the local "parameters" directory):

gsc_G_YY_tTTT_init_param.txt = initialization parameters

(e) Visualization controls: These radio buttons permit to visualize the spherical model, the referential axes (a, d, l), the 3 orientation planes and the coordinates origin extracted in processing step 2.

(f) Hand-correction controls: These editable text fields allow to manually correct the rotation and offset of the initialization parameters extracted in processing step 2. These corrections can be saved by pressing the "Save init parameters" button.

(g) Visualization of the chosen analyzed embryo together with the options specified in the menu on the left.

(h) Camera toolbar: It allows to zoom in and out, orbit around the object and/or translate the camera point of view.

Registration Tab:

This tab performs the image registration between analyzed embryos and templates to further refine the initial alignment extracted in processing step 2.

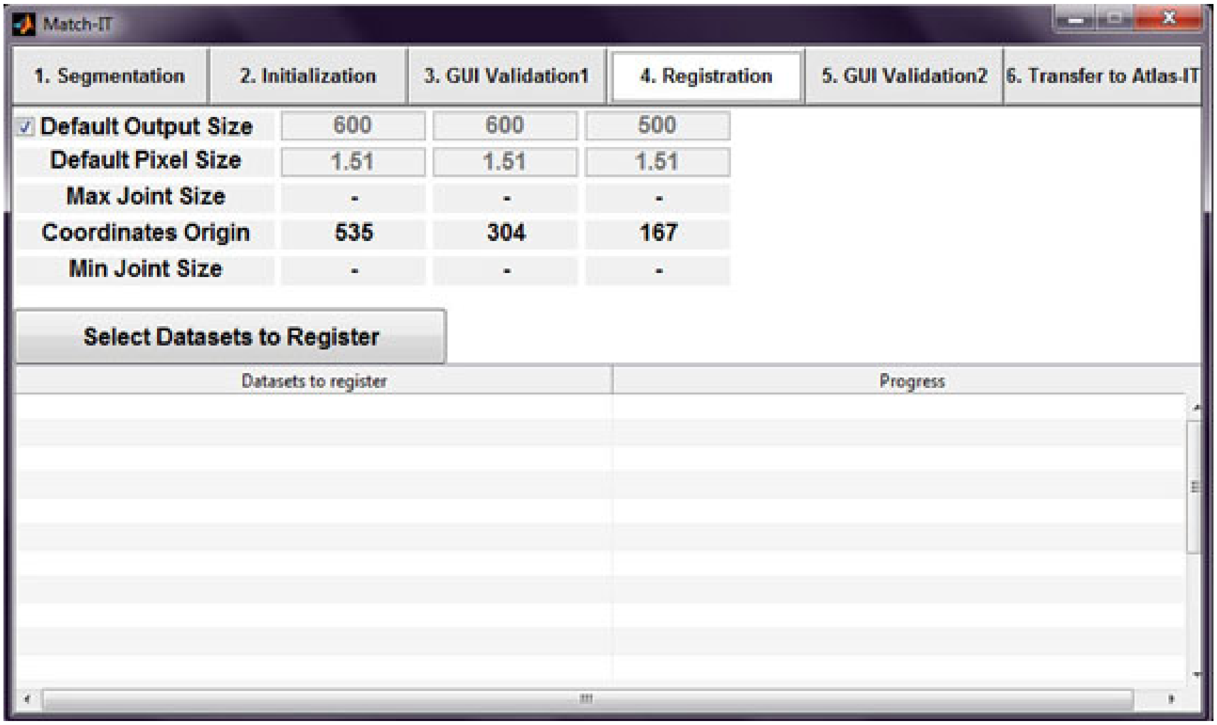

Registering an analyzed embryo onto the template requires the previous extraction of the common orientation planes in processing step 2. Consequently, pressing the "Select Datasets to Register" button will only let you choose among those datasets having already an initialization parameters text file:

- gsc_G_YY_tTTT_init_param.txt = initialization parameters

in the local "parameters" folder. Note that this file can have been corrected by the user in step 3. Apart from the initialization parameters, this registration step also requires both the corresponding analyzed embryo nuclei coordinates and the nuclei shape segmentation image present in the local "data" folder:

- gsc_G_YY_tTTT_ch00.emb = nuclei coordinates
- gsc_G_YY_tTTT_ch03.vtk = nuclei shape segmentation

Then, this registration step maps all analyzed embryos and templates images into a common space having the following default dimensions:

- 600x600x500 pixels
- 1.51x1.51x1.51 microns/pixel

Users can modify these default values by unchecking the box placed at the top left corner. This action will also display the recommended dimensions to specify according to 3 factors: 1) the pixel size specified by the user, 2) the initialization parameters of all the analyzed embryos processed so far and 3) the coordinates origin of the atlas (defined by default as the coordinates origin for the template of the latest temporal stage available). Finally, the output of this fourth processing step is a text file in the local "parameters" folder which holds the extracted registration information:

- gsc_G_YY_tTTT_reg_param.txt = registration parameters

Validate Registration Tab:

This tab starts the Match-IT Graphical User Interface (GUI) to supervise the result of the mapping procedure and correct it if necessary.

The components of the GUI are:

(a) Dataset selector: It pops up a menu to choose which analyzed embryo to visualize. It requires to read the corresponding nuclei coordinates of both the analyzed embryo (displayed in white) and the template (displayed in blue) from the files placed at the local "data" folder:

gsc_G_YY_tTTT_ch00.emb = nuclei coordinates

gsc_tem_YY_tTTT_ch00.emb = nuclei coordinates (template)

(b) Time selector: It allows to slide through different developmental stages of the same dataset (i.e. of the same gene expression G)

(c) Parameter selector: It pops up a dialog to choose the folder where to find the initialization and registration parameters text files (by default this folder is set to the local "parameters" directory):

gsc_G_YY_tTTT_init_param.txt = initialization parameters

gsc_G_YY_tTTT_reg_param.txt = registration parameters

(d) Mapping selector: This radio button permits to visualize the analyzed embryo mapping into the template before and after the registration procedure in step 4.

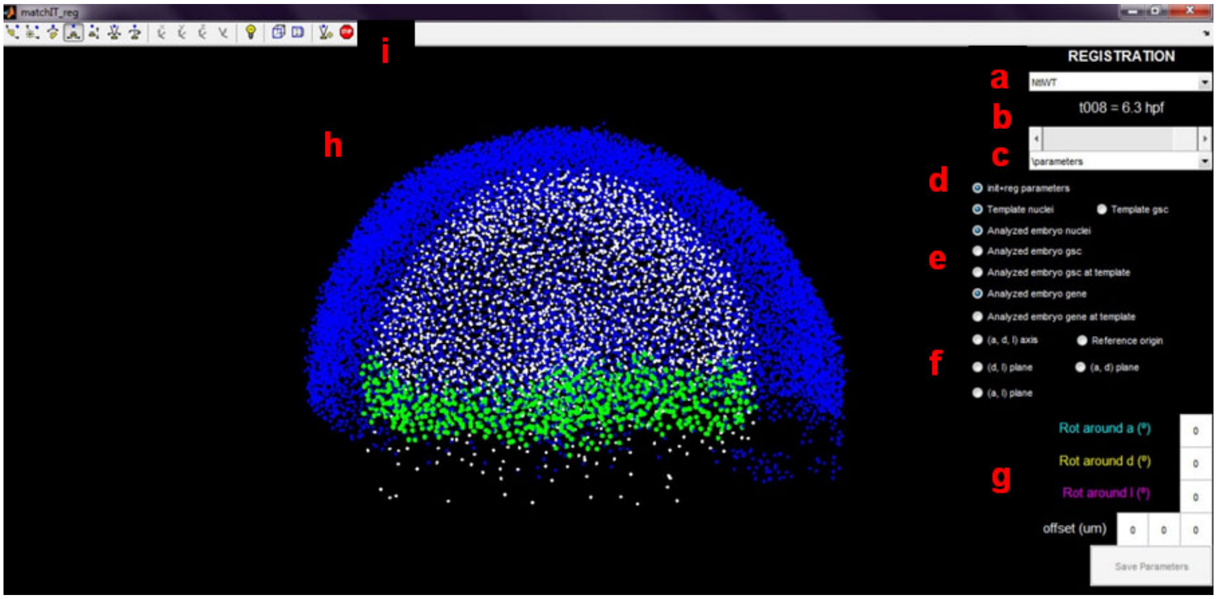

(e) Cell selectors: These radio buttons permit to display/hide several different selections of cells: 1) Template nuclei (in blue), 2) Template gsc expression (in red), 3) Analyzed embryo nuclei (in white), 4) Analyzed embryo gsc expression (in red), 5) Template nuclei determined to express the superimposed analyzed embryo gsc (in red) , 6) Analyzed embryo G expression (in green) and 7) Template nuclei determined to express the superimposed G expression (in green). Consequently, these radio buttons make use of the following files stored in the local "data" folder (in addition to the aforementioned nuclei coordinates files):

gsc_G_YY_tTTT_ch01.csv = selection of cells expressing gsc

gsc_G_YY_tTTT_ch02.csv = selection of cells expressing G

gsc_tem_YY_tTTT_ch01.csv = cells expressing gsc (template)

(f) Visualization controls: These radio buttons permit to visualize the spherical model, the referential axes (a, d, l), the 3 orientation planes and the coordinates origin extracted in processing steps 2-4.

(g) Hand-correction controls: These editable text fields allow to manually correct the rotation and offset of the mapping parameters extracted in processing step 2-4. These corrections can be saved by pressing the "Save parameters" button.

(h) Visualization of the chosen analyzed embryo and/or template together with the options specified in the menu on the right.

(i) Camera toolbar: It allows to zoom in and out, orbit around the object and/or translate the camera point of view.

Transfer to Atlas-IT Tab:

This tab creates the final 3D atlas model that can be visualized in our Atlas-IT software: It determines which template nuclei express each of the mapped gene expressions coming from the analyzed embryos and it also transforms the original raw files and their segmentations into their newly shared "atlas" space.

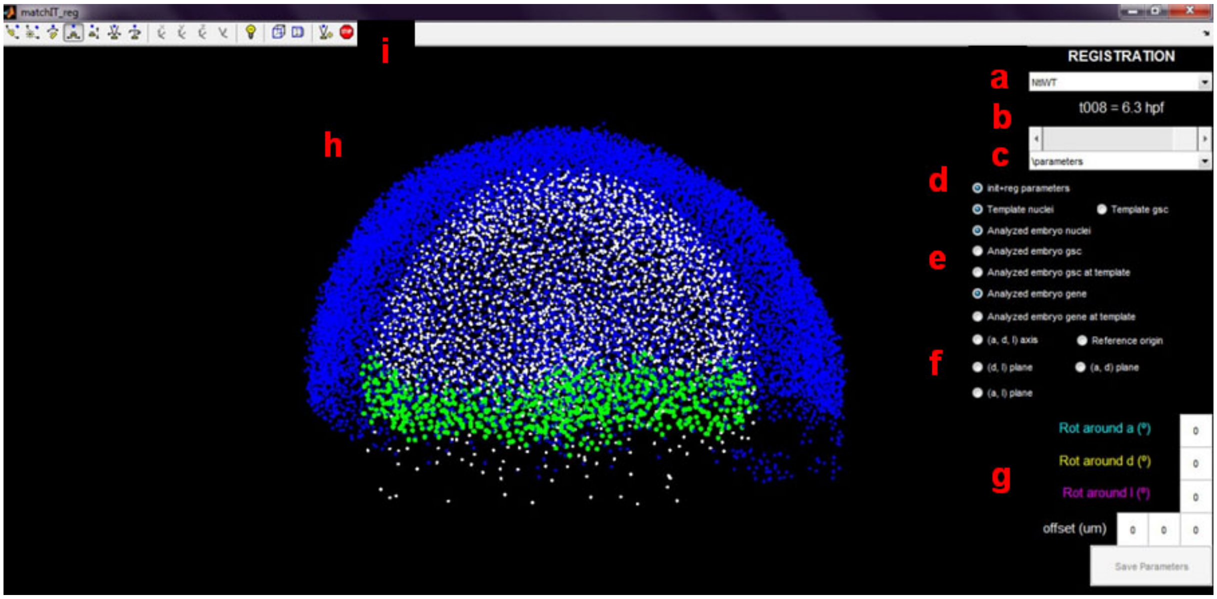

Transferring an analyzed embryo into the final atlas model requires at least having finished the registration process in step 4. Consequently, pressing the "Transfer Results to Atlas-IT" button will only let you choose among those datasets having already a registration parameters text file:

- gsc_G_YY_tTTT_reg_param.txt = registration parameters

in the local "parameters" folder. Note that this file can have been corrected by the user in step 5. Apart from the registration parameters, this final step also requires both the corresponding template nuclei coordinates and the selection of analyzed embryo cells expressing gsc and G:

- gsc_G_YY_tTTT_ch01.csv = selection of cells expressing gsc
- gsc_G_YY_tTTT_ch02.csv = selection of cells expressing gene G

The result of this final step is a text file that indicates, for each of the template nuclei constituting the final model, whether they express or not each of the mapped gsc and G expressions coming from the cohort of analyzed embryos:

- 3DAtlas_Localn0t1.emb = final atlas model

which is stored in the local "cache" folder. This file can be read by our Atlas-IT software in order to display the resulting 3D atlas together with all the mapped gene expressions. Apart from that, this final step also transforms all the analyzed embryos raw files and their segmentations to the new common "atlas" space:

- gsc_G_YY_tTTT_ch00.vtk = raw nuclei channel
- gsc_G_YY_tTTT_ch01.vtk = raw gsc channel
- gsc_G_YY_tTTT_ch02.vtk = raw gene channel
- gsc_G_YY_tTTT_ch03.vtk = segmented nuclei shape
- gsc_G_YY_tTTT_ch04.vtk = gsc segmentation

- gsc_G_YY_tTTT_ch05.vtk = gene segmentation

which are all stored in the local "data" folder. The results are the following .vtk files:

- gsc_G_YY_tTTT_ch00_trans.vtk = mapped raw nuclei channel
- gsc_G_YY_tTTT_ch01_trans.vtk = mapped raw gsc channel
- gsc_G_YY_tTTT_ch02_trans.vtk = mapped raw gene channel
- gsc_G_YY_tTTT_ch03_trans.vtk = mapped nuclei shape
- gsc_G_YY_tTTT_ch04_trans.vtk = gsc segmentation
- gsc_G_YY_tTTT_ch04_trans.vtk = mapped gsc segmentation
- gsc_G_YY_tTTT_ch05_trans.vtk = mapped gene segmentation

which are saved in the local "cache" folder and can be directly visualized in Atlas-IT together with the final atlas model.

File formats

A. **The "config.txt" file:**

```
# Configuration file for Match-IT application
DATASET_LIST tem_wt sox32_wt tbx16_wt oep_wt snaila_wt foxa2_wt ntla_wt flh_wt egfp_wt
TIME_LABELS 0hpf 0hpf 0hpf 4hpf 4.3hpf 4.7hpf 5.3hpf 6hpf 6.3hpf
THS_TEM_GSC 0 0 0 50 80 100 60 60 100
THS_NTLA_GSC 0 0 0 80 110 180 80 80 50
THS NTLA GENE 0 0 0 90 170 150 155 160 60
```

B. **The ".vtk" files:**

```
# vtk DataFile Version 3.0
created by writeVTK
BINARY
DATASET STRUCTURED_POINTS
DIMENSIONS 1024 1024 196
ORIGIN 0.000000 0.000000 0.000000
SPACING 0.586000 0.586000 1.167000
POINT_DATA 205520896
SCALARS image_data unsigned_char
LOOKUP_TABLE default
NULNULNULNULNULNULNULNULNULNUL
ACKEOTNULSOHNULNULNULNULNULNUL
```

C. **The ".csv" files:**

```
14325;0;8;315;180;196;0;-1
14326;0;8;347;180;196;0;-1
14327;0;8;298;187;196;0;-1
14328;0;8;281;191;196;0;-1
14329;0;8;715;191;196;0;-1
14330;0;8;717;192;196;0;-1
14331;0;8;402;193;196;0;-1
14332;0;8;347;203;196;0;-1
14333;0;8;697;206;196;0;-1
14334;0;8;362;213;196;0;-1
14335;0;8;758;214;196;0;-1
14336;0;8;654;215;196;0;-1
```

D. **The "3DAtlas_Localn0t1.emb" file:**

```
#id;id_t;x;y;z;t;0;-1;;tem_wt_ch01;ntla_wt_ch01;ntla_wt_ch02
1;1;238;316;273;3;0;-1;0;0;0
2;2;220;297;263;3;0;-1;0;0;0
3;3;252;298;277;3;0;-1;0;0;0
4;4;251;312;277;3;0;-1;0;0;0
5;5;240;332;271;3;0;-1;0;0;0
6;6;237;299;269;3;0;-1;0;1;0
7;7;261;323;279;3;0;-1;0;0;0
8;8;294;323;292;3;0;-1;0;0;0
9;9;232;285;266;3;0;-1;0;0;0
```

Troubleshooting

1. Read this manual carefully.
2. Check that the "config.txt" values have been properly set and verify that the input files to each of the processing steps (highlighted in bold throughout this manual) have been properly created and hold the right format.
3. Inspect the "C:/matchit.log" file to find out the cause of the problem.
4. Check the Match-IT web page for updated versions, data or video examples: bioemergences.iscpif.fr
5. Contact ccastro at die.upm.es
