# Supplementary material for: A Digital Framework to Build, Visualize and Analyze a Gene Expression Atlas with Cellular Resolution in Zebrafish Early Embryogenesis
Source: PLoS Comput Biol. 2014 Jun 19;10(6):e1003670. doi: 10.1371/journal.pcbi.1003670 (PMC4063669; doi:10.1371/journal.pcbi.1003670)
Supplement: User Guide S2 — Atlas-IT User Guide: A step-by-step protocol. This user guide is available as Supplementary Material to this paper and at http://bioemergences.iscpif.fr/documents/AtlasIT.zip. (PDF) [file pcbi.1003670.s035.pdf]

A digital framework to build, visualize and analyze a gene expression atlas with cellular resolution in zebrafish early embryogenesis

Carlos Castro-González[1,2,3], Miguel A. Luengo-Oroz[1,2], Louise Duloquin[4,5,6], Thierry Savy[4,5,6], Barbara Rizzi[4,5,6], Sophie Desnoulez[4,6], René Doursat[5,6,7], Yannick L. Kergosien[4,6,8], María J. Ledesma-Carbayo[1,2], Paul Bourgine[4,5,6], Nadine Peyriéras[4,5,6,*,†], Andrés Santos[1,2,*,†]

2 User Guide S2 - Atlas-IT User Guide: A step-by-step protocol

What is Atlas-IT?

Atlas-IT is a software package to visualize and validate cellular-level atlases of gene expression in early embryogenesis. Atlas-IT was delivered as part of the following publication: C. Castro-Gonzlez, M.A. Luengo-Oroz, L. Duloquin, T. Savy, B. Rizzi, S. Desnoulez, R. Doursat, Y. Kergosien, M.J. Ledesma-Carbayo, P. Bourgine, N. Peyriras and A. Santos. "A digital framework to build, visualize and analyze gene expression atlases with cellular resolution in zebrafish early embryogenesis".

A public release of Atlas-IT together with the present tutorial and representative datasets are available as Supplementary Material to this paper:

http://bioemergences.iscpif.fr/documents/AtlasIT.zip

System requirements

- Windows or MacOSX 64 bits.
- A 64-bit version of Java is also required. Make sure it is included in the system path. In case you don't have Java installed, you can get it here: https://www.java.com/en/download/
- 8GB RAM (minimum)
- The disk space needed to load the provided example data and to run Atlas-It on it is approximately 250MB

Installing Atlas-IT

To run the program, simply unzip the software package and double-click on "Atlas-IT.exe". Representative datasets can be found within the "dataset" folder of the Atlas-IT package.

Using Atlas-IT

Creating a new atlas experiment:

In order to create a new atlas experiment, go to the "dataset" directory of the Atlas-IT package and create a new folder named:

YYMMDDa_Local = experiment folder

where:

- "YY" is a 2-letter code designating the experiment year.
- "MM" is a 2-letter code designating the experiment month.
- "DD" is a 2-letter code designating the experiment day.

- "a" is a 1-letter code which can take any free value.

Inside the newly created directory, one subfolder and two files must be created:

- YYMMDDa_Local/vtk = raw data folder
- YYMMDDa_Localnt01.emb = atlas text file
- parameters.txt = parameters file

The raw data folder contains the transformed raw data of those embryos which have been aligned to the common atlas model. The atlas text file contains the cellular-level information about where and when the different genes in the atlas model express. The parameters file contains information about the experiment name, size and temporal and spatial resolution (see the "File formats" section in this tutorial for more information).

After these files and folders have been created, a new experiment becomes available in the Atlas-IT starting menu:

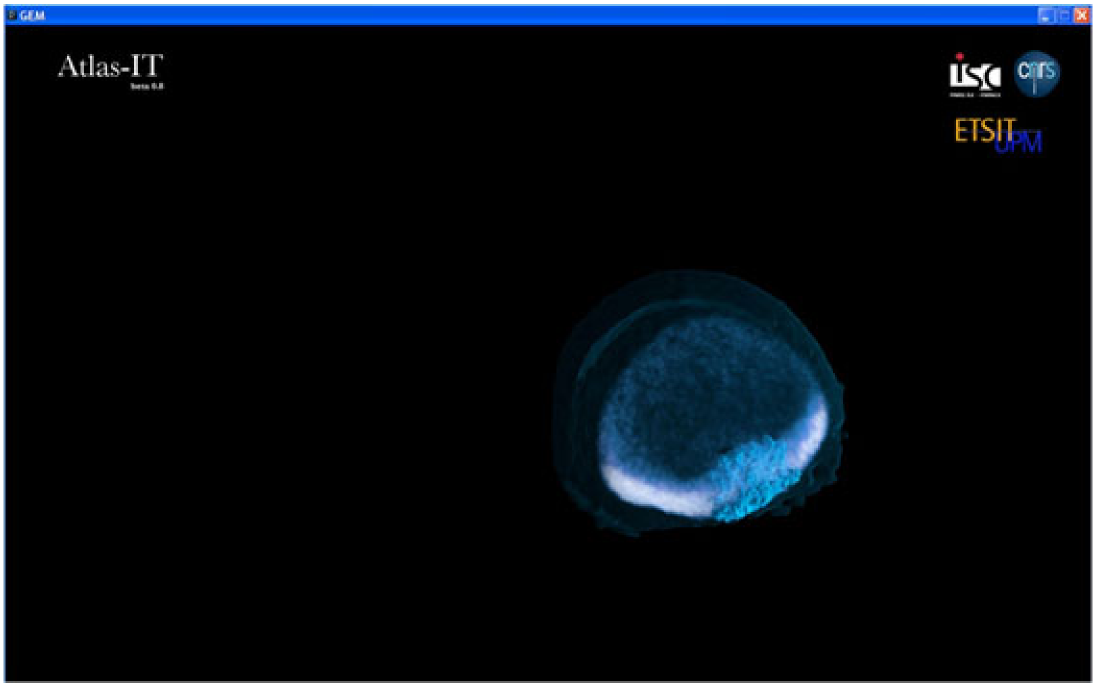

Select one experiment from this starting menu to start exploring the atlas model.

Exploring the atlas model:

Atlas-IT presents the embryo nuclei positions in space and time and can highlight the nucleus population expressing a certain gene and simultaneously display the original raw data from which this information was incorporated to the atlas.

Here there is a list of some of the controls to explore the atlas model:

- mouse pressed = turn the atlas around
- mouse + shift = translate the atlas

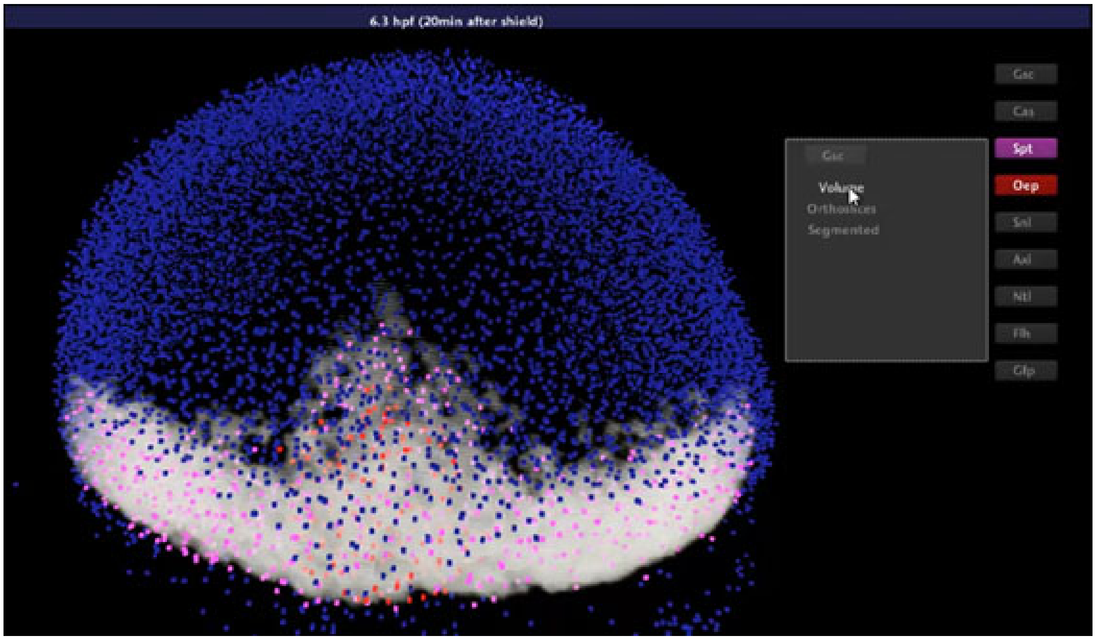

- mouse + alt = zoom on the atlas
- right click = focus on one cell
- up arrow = move forward in time
- down arrow = move backward in time
- c = change the atlas color for better contrast
- r-f = moves XY orthoslice
- g-v = moves YZ orthoslice
- t-y = moves ZX orthoslice

The top bar indicates the time step. Specific developmental stage nomenclature can be defined by editing the "times.txt" file in the "dataset" folder.

The right panel contains one button for each of the gene products present at the "YYMMDDa_Localnt01.emb" atlas text file (see File Formats). Clicking on one of these buttons displays a menu where we can select to visualize the volume and/or orthoslices of the analyzed embryo expressions mapped to the atlas model. We can also choose to highlight nuclei populations expressing any of the two gene expressions for which every analyzed embryo was labeled. One of these two expressions, named goosecoid (gsc), is present at all analyzed embryos as it was employed as reference during the mapping procedure.

File Formats:

A. **The "parameters.txt" file:**

```
120611a_Local:Name
3D Atlas and gene expression:Comments
8:Timesteps
512:X,Y pixels
250:Z pixel
1.51:X,Y pixel size
1.51:Z pixel size
1200.0:Delta T
```

B. **The "YYMMDDa_Localn0t1.emb" atlas file:**

```
#id;id_t;x;y;z;t;0;-1;;tem_wt_ch01;ntla_wt_ch01;ntla_wt_ch02
1;1;238;316;273;3;0;-1;0;0;0
2;2;220;297;263;3;0;-1;0;0;0
3;3;252;298;277;3;0;-1;0;0;0
4;4;251;312;277;3;0;-1;0;0;0
5;5;240;332;271;3;0;-1;0;0;0
6;6;237;299;269;3;0;-1;0;1;0
7;7;261;323;279;3;0;-1;0;0;0
8;8;294;323;292;3;0;-1;0;0;0
9;9;232;285;266;3;0;-1;0;0;0
```

Troubleshooting:

1. Read this manual carefully.
2. Press "h" to access the program help.
3. Check the Atlas-IT web page for updated versions, data or video examples: bioemergences.iscpif.fr
